# Shining light on the mechanism of photochemical alkene formation in vitamin B_12_

**DOI:** 10.1039/d5sc07054f

**Published:** 2026-01-23

**Authors:** Alivia Mukherjee, Summer Y. Wu, David J. Cooper, Rachel Hendrickson, Roseanne J. Sension, Nicolai Lehnert, James E. Penner-Hahn

**Affiliations:** a Department of Chemistry, University of Michigan Ann Arbor Michigan 48109-1055 USA lehnertn@umich.edu jeph@umich.edu; b Department of Biophysics, University of Michigan Ann Arbor Michigan 48109-1055 USA; c Department of Physics, University of Michigan Ann Arbor Michigan 48109-1040 USA

## Abstract

A wide range of proteins and enzymes depend on alkylcobalamins (alkylCbl or vitamin B_12_) for their activities, owing to the unique, biologically relevant Co–C bond. CarH, a regulatory protein in the bacterial carotenoid biosynthetic pathway, utilizes the photosensitivity of the Co–C bond in adenosylcobalamin (AdoCbl) for gene regulation. B_12_-dependent reductive dehalogenases rely on chemical Co–C bond cleavage to form a Co(iii)–halide bond during catalysis. Ultrafast spectroscopy demonstrates that photolytic Co–C bond cleavage in cobalamins begins with the generation of a Co(ii) species and an alkyl radical. Interestingly, both CarH and reductive dehalogenases are thought to generate a highly reactive Co(i) species as part of their reactivity. We have used time-resolved measurements of alkylCbls under single turnover conditions to better characterize this reactivity. We demonstrate that Co(i) can be generated in nearly quantitative yield during anaerobic photolysis of alkylCbls in aqueous solution. Remarkably, the addition of alkyl halide to this Co(i) species does not produce quantitative yield of Co(iii)-alkylCbl species as would be expected given the “supernucleophilic” nature of the Co(i) center. Instead, we find a branching pathway which has significant implications in Cbl-dependent enzymes and vitamin B_12_ based organometallic photochemistry. Finally, we demonstrate that both the final oxidation state of the cobalamin product and the fate of the organic radical formed are solvent-dependent, an observation that has implications for CarH photochemistry.

## Abstract 2

Initial Co(ii) formation followed by solvent-dependent Co(i) formation.

## Graphical abstract

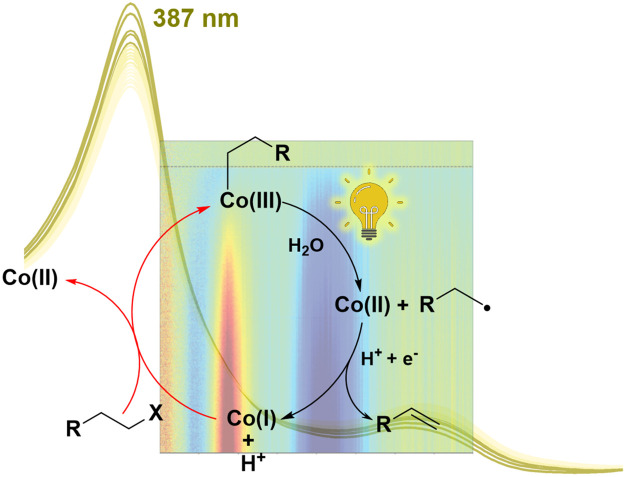

## 1 Introduction

**Fig. 1:**
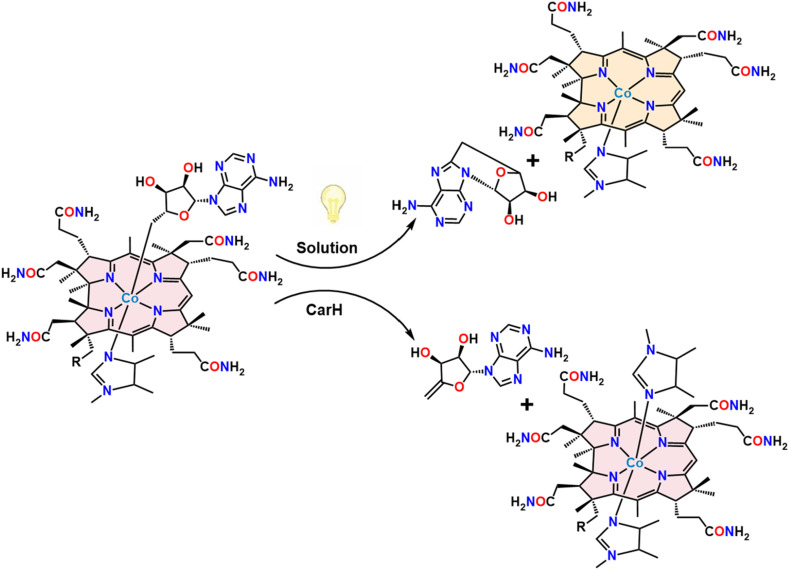
Products obtained by photolysis from free AdoCbl in solution and from AdoCbl bound in the CarH protein. The structures have been truncated for clarity.

Cobalamin (vitamin B_12_) is essential in various biological processes, ranging from gene regulation to metabolic transformations.^1^ Known to be the most structurally intricate vitamin, it is involved in a myriad of different chemical reactions, including carbon skeleton isomerization, dehalogenation, halide coupling, hydrogenation, transmethylation, and cyclopropanation.^2^ Cobalamin derivatives, particularly alkylcobalamins (alkylCbls), have also garnered significant attention due to their pivotal roles as coenzymes in various biological processes including gene regulatory and dehalogenation pathways.^3–7^ Among their versatile functions, alkylCbls have been explored for their behavior upon exposure to light.^8^ Although the photosensitivity of alkyl derivatives of vitamin B_12_ has long been observed, it has recently been found to be biologically relevant in the regulation of the carotenoid biosynthetic pathway in certain bacteria.^1^ In the past decade, it has been shown that the photosensitivity of adenosylcobalamin (AdoCbl) plays a crucial role in gene regulation for certain non-photosynthetic bacteria *via* the transcription regulatory protein, CarH.^7,9^ The underlying mechanism behind this intriguing photochemistry is not fully understood, making it compelling for further investigation. Upon photolysis, the bond between the central cobalt atom and the adenosyl group cleaves, where the light induced cleavage generates Co(i) and 4′,5′-anhydroadenosine (alkene product). This outcome is contrary to that observed upon AdoCbl photolysis in solution, which typically produces 5′-deoxyadenosyl radicals that cyclize to form 5′,8-cycloadenosine (cyclic product) alongside a Co(ii) species (Fig. 1).^10–18^ This divergence highlights fundamental gaps in our understanding of how protein environments and even experimental reaction conditions like solvents dictate cobalamin reactivity and product selectivity. The alkene product, 4′,5′-anhydroadenosine, has also been observed in adocobinamide (coenzyme B_12_ with the axial base removed) thermolysis in ethylene glycol. It was found that thermolysis of adocobinamide generates 5′-deoxyadenosyl radicals that are trapped within a solvent cage before undergoing β-H elimination.^19,20^ Base-mediated Co–C bond cleavage in 5′-deoxyadenosylcobaloxime,^21^ and AdoCbl bound to the enzymes ethanolamine deaminase and d,l-propanediol hydrolase^22^ were all reported to give the alkene as the observed product. Hoffman and co-workers have suggested an explanation for these differences. They trapped the 5′-deoxyadenosyl radical that was formed in a radical S-adenosyl-l-methionine enzyme and showed that it had the C5′–H antiperiplanar to the ribose-ring oxygen, potentially stabilizing the radical against elimination of the 4′−H; DFT calculations suggested that this might be an intrinsic feature of AdoCbl.^23^ In this model, the typical behavior of the 5′-deoxyadenosyl radical is intramolecular cyclization. While this explains the behavior of AdoCbl in solution, it makes the production of the alkene during photolysis of AdoCbl in CarH all the more surprising. Drennan and co-workers favor a homolytic pathway for Co–C bond cleavage, although this would require a different geometry for the 5′-deoxyadenosyl radical in order to explain the alkene product.^10,19,24,25^ Kutta and co-workers have suggested a heterolytic bond cleavage pathway for the Co–C bond in CarH, leading to the formation of ionic intermediates in the microsecond to millisecond timescales.^26^ A third possible pathway would be concerted β-elimination where a hydride ion directly forms hydridocobalamin and 4′,5′-anhydroadenosine, bypassing a radical pair intermediate. However, this was deemed unlikely as it would typically require a vacant coordination site on the metal and lacks prior examples in organocobalamins.^27,28^ The final piece of the puzzle involves the cobalt center. Sension and co-workers showed that with 550 nm excitation, the cobalamin in CarH forms an excited state with an unusually long lifetime (up to 3.8 ns), perhaps implicating the Co directly in the preference for alkene formation.^29^

Although alkene formation following AdoCbl photolysis was suggested to be unique to CarH-type proteins, the generation of alkene products through the photolytic cleavage of simple alkylCbls is not a novel phenomenon. Over 50 years ago, alkenes were shown to be the product in the photolysis of alkylCbls such as *n*-ethylcobalamin.^21,30–34^ It has been proposed that alkene formation occurs *via* a Co(iii)-hydride intermediate (which could be alternatively formulated as a protonated Co(i)); however, definitive evidence for such a species has yet to be reported.^10,35–37^ Recently, West and co-workers expanded on this proposal by demonstrating the photocatalytic synthesis of a range of primary alkenes from cobalamin and alkyl bromides in organic solvents, noting that the weak base HCO_3_^−^ was necessary in order to favor alkene over alkane formation.^38^

In addition to uncertainty over the mechanism of alkene formation, there is also uncertainty over the oxidation state(s) of the Co center. As noted above, ultrafast photochemistry has shown that on the picosecond timescale, photolysis of alkylCbls results in the formation of a geminate pair of Co(ii) + alkyl radical, followed by either recombination or cage-escape to form the Co(ii) ground state, with no evidence for Co(i), at least not on the nanosecond time scale.^39^ In contrast, West and co-workers, in their work mentioned earlier, suggested a scheme similar to one of those proposed for CarH, with the Co cycling between Co(iii)-alkyl and Co(i). In this mechanism, the reductant sodium borohydride was required, but only to initiate the cycle. This too echoes early observations of the photochemical formation of a Co(i) species alongside Co(ii) in an aqueous solution of *n*-amylcobalamin under anaerobic conditions.^8^ This earlier work, substantiated by a single UV-Vis absorption spectrum, has not been widely appreciated within the scientific community. However, very recently CarH has been shown to form Co(i) on the millisecond time scale following photolysis.^40^ Furthermore, the reactivity of cobalamin extends to its crucial role in reductive dehalogenases (RDases), enzymes that play significant roles in enabling organohalide-respiration by catalyzing the removal of halogens from organic substrates. Understanding the mechanism involved in cobalamin-dependent reductive dehalogenation is essential, as this process underpins microbial strategies for detoxifying halogenated environmental pollutants. While several mechanistic pathways have been proposed, all center on Cob(i)alamin as the key reactive species. One proposed pathway involves Co(i) acting as a nucleophile, to form an organocobalt adduct; here, cobalt directly attacks the carbon backbone of the organohalide leading to alkylation of cobalt *via* the formation of a Co–C bond.^6^ This intermediate is then thought to be reduced further *via* electron transfer from an iron-sulfur cluster. Although organocobalt intermediates have been identified in solution studies, their existence during enzymatic catalysis has not been experimentally confirmed.^41–43^ Alternatively, studies have suggested a long range electron transfer, wherein Cob(I)alamin donates an electron to a substrate generating a radical intermediate, followed by the elimination of the halogen.^44–48^ A third mechanism proposes that Cob(I)alamin initiates nucleophilic attack on the halogenated substrates, abstracting a halide and forming a Co(iii)-halide intermediate.^49,50^ This mechanism is also suggested by Leys and co-workers, whereby they postulate that carbon–halogen bond cleavage (either homolytically or heterolytically) and subsequent electron and proton transfers yield the dehalogenated product, with the pathway likely depending on the structure of the substrate.^51^ In all these cases, the active nucleophilic Cob(I)alamin form must be regenerated to sustain the catalytic cycle. Although structural analyses have helped to characterize these enzymes and suggest potential mechanisms, persistent gaps remain in our understanding of the dynamic steps in enzymatic dehalogenation.^51^ Critical uncertainties remain, such as the real-time sequence of bond-breaking and bond-forming events, and the precise nature of electron transfers involved, which can only be resolved through application of time-resolved spectroscopies or dynamic structural methodologies. Importantly, the presence of external electron donors in experimental setups often complicates the interpretation of Co(i) reactivity, as they can continuously regenerate the active form, masking potentially relevant intermediates.

To better understand the mechanism by which alkylCbl favors alkene formation following photolysis, and how this depends on experimental conditions, we have studied the millisecond to second reactivity of three alkylCbls: the well-studied propylcobalamin (PrCbl) along with two novel alkylCbl derivatives, phenylhexylcobalamin (PhHxCbl) and naphthylethylcobalamin (NapEtCbl). The latter two derivatives were selected for comparatively straightforward product identification since the aromatic groups in these compounds facilitate easy product analysis using gas chromatography coupled with mass spectrometry (GC-MS). Our study investigates the chemically reactive species that participate in the photochemistry of alkene formation from alkylCbls and aims to determine the extent to which the nature of the organic product correlates with the final Co oxidation state under anaerobic conditions. Our approach leverages the use of different solvent conditions to understand how experimental conditions affect the formation of the organic product(s). Our observations indicate that in water, a Co(i) species rapidly forms within seconds after the initiation of photolysis of alkylCbls, whereas Co(ii) forms and remains unchanged in organic solvents including dimethyl sulfoxide (DMSO), ethanol (EtOH), and methanol (MeOH). We demonstrate that organic radical chemistry plays a crucial role in determining product selectivity under single turnover conditions. Finally, since we were able to generate a clean sample of cob(I)alamin by photolysis without the need for excess reductant, we investigated the reaction of this species with alkyl halides to obtain insight into the properties of Co(i) as a supernucleophile. Strikingly, we were able to observe a divergent reaction pathway rather than the typical S_N_2 reaction mechanism exhibited by the Co(i) nucleophile. Our mechanistic study has significant implications in not just cobalamin photochemistry but also its dehalogenase activity.

## 2 Experimental methods

### 2.1 Synthesis of alkylcobalamins

The synthesis and workup for these alkylCbls were performed in a dark room under red light. Synthesis and purification of phenylhexylcobalamin (PhHxCbl), naphthylethylcobalamin (NapEtCbl), and *n*-propylcobalamin (PrCbl) were carried out using a combination of different literature methods.^33,34,52–55^

### 2.2 Static and time-resolved spectral measurements

Static UV-Vis spectra were measured using a dual beam Shimadzu UV-Visible spectrophotometer (Shimadzu UV-2600 spectrometer) at room temperature. Time-resolved spectra were measured using an Avantes diode array (AvaSpec-2048-USB2-UA) coupled to the cuvette *via* a fiber optic cable. The ultraviolet and visual continua used for the photolysis measurements were generated with a Xenon arc lamp and a combination of various lenses and filters; the lamp was used both as our pump and probe. Using a beam splitter, 96% of the light was directed through a glass UV filter to photolyze the samples (∼1.2 mW at 532 nm), the remaining unfiltered 4% of the beam was used for absorption measurements. The 4% probe beam did not cause any measurable photolysis. Absorbance was calculated as Absorbance = −log_10_ [(*I*(*t*) − *I*_dark_ − *I*_scatter_)/(*I*_lamp_ − *I*_dark_)] for *t* > 0 and as Absorbance = −log_10_ [(*I*(*t*) − *I*_dark_)/(*I*_lamp_ − *I*_dark_)] for *t* < 0, where *I*_dark_ is the dark current of the diode array and *I*_lamp_ is the signal in the absence of sample. *I*_scatter_ represents the scatter from the photolysis beam and was measured as the difference in *I*(*t*) averaged 1 s after and 1 s before *t* = 0.

To show the spectral evolution as a function of time, time-resolved difference spectra were generated by calculating the initial absorbance spectrum of the sample prior to exposure to the photolysis beam. This was then subtracted from the absorbance spectra obtained during photolysis.

To investigate the mechanism of alkylCbl synthesis, we used photolytically generated Co(i) and conducted the reactions with 1-bromopropane as the alkyl halide, due to its relatively high solubility in water (∼20 mM). For these experiments, a PrCbl sample (typically between 0.03 to 0.06 mM) was photolyzed in water for 3 minutes to generate the Co(i) species.

### 2.3 GC-MS Analysis

Samples were prepared and photolyzed under anaerobic conditions as described above. After photolysis, a small-scale liquid–liquid extraction was performed to extract the organic photoproduct into hexanes. Samples photolyzed in EtOH or MeOH were either passed through a small silica column to collect the organic product or extracted into hexane. Gas chromatography-mass spectrometry (GC-MS) analyses were performed using a Shimadzu QP-2010 GC-MS equipped with a 30 meter-long DB-5 column with 0.25 mm ID (see the Supplemental Information, SI, for separation method). Fragmentation patterns were used to identify the peaks for 6-phenyl-1-hexene and phenylhexane. Chromatograms with total ion counts and extracted ion counts at specific *m*/*z* values are available in the SI.

## 3 Results and analysis

### 3.1 Photolysis of alkylCbl in water under anaerobic conditions rapidly converts alkylCbl into a Co(i) species and the alkene product

**Fig. 2:**
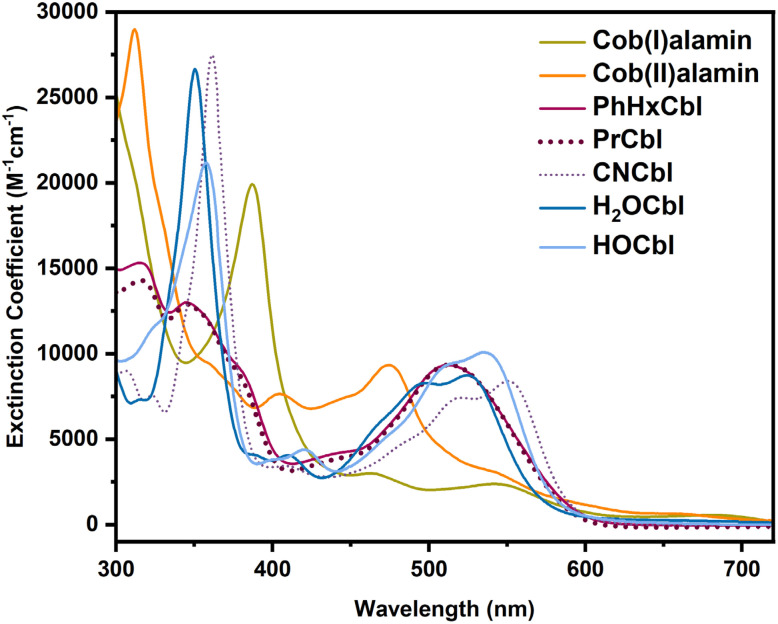
UV-Vis absorption reference spectra for cob(I)alamin, cob(II)alamin, PhHxCbl, PrCbl, CNCbl, H_2_OCbl, and HOCbl in water.

**Fig. 3:**
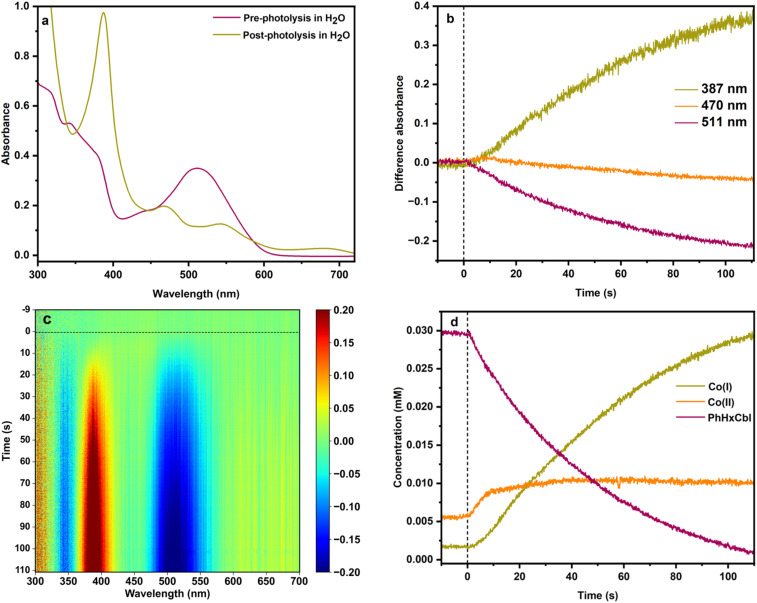
(a) Steady-state UV-Vis absorption spectra for PhHxCbl before and after photolysis in water. (b) Change in difference absorbance over time at specific wavelengths corresponding to different species, 387 nm (Co(i)), 470 nm (Co(ii)), and 511 nm (Co(iii)). (c) Heat map depicting time-resolved (ms to s) UV-Vis difference absorption spectra for PhHxCbl in water. The growth of the yellow-red region shows the generation of Co(i) while the bleach for Co(iii) is represented by the blue region. (d) Fitted concentrations of Co(i), Co(ii), and PhHxCbl shown over 2 minutes of photolysis in water.

The different oxidation states of cobalamin have distinct UV-Vis spectra that can be used to distinguish between these forms. Representative spectra for Co(i), Co(ii), PhHxCbl, PrCbl, cyanocobalamin (CNCbl), aquocobalamin (H_2_OCbl), and hydroxocobalamin (HOCbl) are shown in Fig. 2. We observed that alkylCbl converts to Co(i) within 2 minutes of photolysis in water under anaerobic conditions. For example, upon illuminating PhHxCbl in water at room temperature under strictly anaerobic conditions, we observed an almost immediate change of the sample color from red to very faint yellow. The UV-Vis absorption spectrum of the photolyzed alkylCbl has an intense peak at 387 nm after 2 minutes of illumination, consistent with Co(i) formation.^56^Fig. 3 shows the formation of the Co(i) species (indicated by the absorption maxima at 387 nm, 470 nm, 545 nm, and 680 nm) as the PhHxCbl sample is photolyzed in water. This highly reactive Co(i) species slowly oxidizes to a Co(ii) species (see Fig. S9). Similarly, photolysis of NapEtCbl and PrCbl in water leads to the generation of cob(I)alamin (Fig. S13 and S15, respectively). In contrast to the rapid formation of Co(i), the oxidation of Co(i) to Co(ii) is quite slow, with only ∼17% oxidation to Co(ii) over 90 minutes, as obtained from fitting the data in Fig. S9 using references from Fig. 2.

To better understand the formation of cob(I)alamin, we explored the kinetics of the Co species. Differences in absorption at selected wavelengths that are characteristic of the different oxidation states are shown in Fig. 3b for PhHxCbl in water: Co(i) at 387 nm, Co(ii) at 470 nm, and alkylcobalamin Co(iii) at 511 nm. The time-resolved difference spectra of PhHxCbl photolyzed in water are shown in Fig. 3c.

The loss of the Co(iii) PhHxCbl species starts instantaneously as illustrated by the prompt bleach at 511 nm and 350 nm. In contrast, the growth in Co(i), which gives rise to the peak at 387 nm, is delayed by ∼10 seconds. The same results are observed for the other two alkylCbls we studied (Fig. S24 and S28). There is, however, an increase in Co(ii) concentration in the beginning which slows down over time as Co(i) starts forming (Fig. 3d). To quantitate these changes, the spectra were fitted using the Co(i), Co(ii), and PhHxCbl reference spectra in Fig. 2 (refer to the Supplemental Information for details on the fitting procedure). As shown in Fig. 3d, these fits confirm that there is an initial increase then a plateau in the Co(ii) concentration, and a lag in the formation of the Co(i) species which is the ultimate product of alkylCbl photolysis in water. The rate of Co(iii) loss during the photolysis of alkylCbls in water is well described by first order kinetics, consistent with previous studies.^57,58^ If these experiments are performed in the presence of the radical trap 5,5-dimethyl-1-pyrroline N-oxide (DMPO), Co(i) is not formed and instead the final product is cob(ii)alamin (Fig. S57 and Table S7), demonstrating that the alkyl radical is critical for the formation of Co(i). One possible mechanism for this would be proton-coupled electron transfer (PCET) involving the β-hydrogen of the radical. If this happened, a proton release should accompany Co(i) formation. To test for this, we carried out the photolysis in the presence of phenol-red and observed the expected decrease in pH (Fig. S58–S60), suggesting that a PCET mechanism is operable.^59^

### 3.2 Photolysis of alkylCbl in organic solvents under anaerobic conditions results in its predominant conversion to a Co(ii) species

**Fig. 4:**
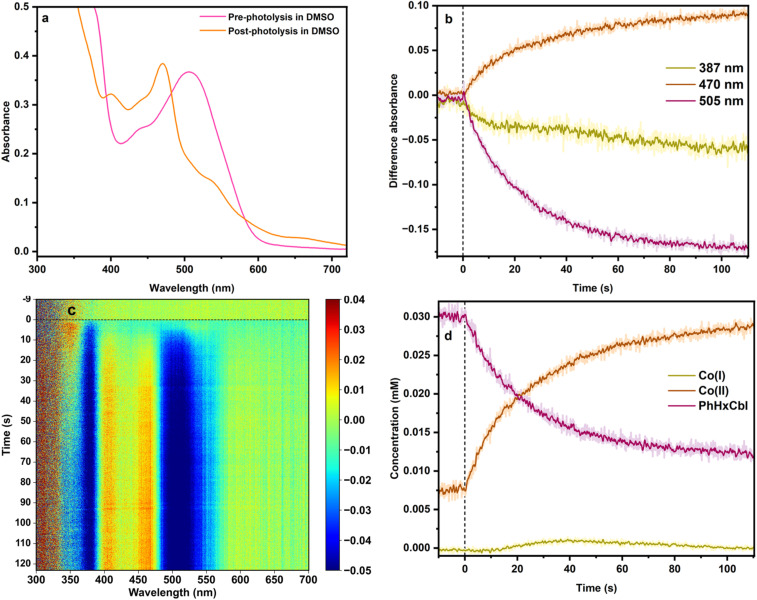
(a) Steady-state UV-Vis absorption spectra for PhHxCbl before and after photolysis in DMSO. (b) Change in difference absorbance over time at specific wavelengths corresponding to different species, 387 nm (Co(i)), 470 nm (Co(ii)), and 505 nm (Co(iii)). (c) Heat map depicting time-resolved (ms to s) UV-Vis difference absorption spectra for PhHxCbl in DMSO. The growth of the yellow-red region shows the generation of Co(ii) while the bleach for Co(iii) is represented by the blue region. (d) Fitted concentrations of Co(i), Co(ii) and Co(iii) over 2 minutes of photolysis in DMSO.

In contrast to the behavior in water, photolysis of alkylCbl in the aprotic solvent DMSO or the protic solvents MeOH and EtOH primarily generates cob(II)alamin (Fig. 4, S17–S21, S25–S27, S30 and S31). As before, there is a prompt loss in absorbance at 505 nm and 360 nm, characteristic of Co(iii) spectra in DMSO (Fig. 4c). However, unlike in water, this spectral change is accompanied by an increase in absorbance at ∼400–470 nm, characteristic of cob(II)alamins.^26,29,35,60^ Analogous results were observed for the other alkylCbls. Quantitative analysis of these data confirms that they reflect primarily a conversion of Co(iii) to Co(ii), with negligible Co(i) formation.

### 3.3 Solvent effects on cobalamin photochemistry

**Fig. 5:**
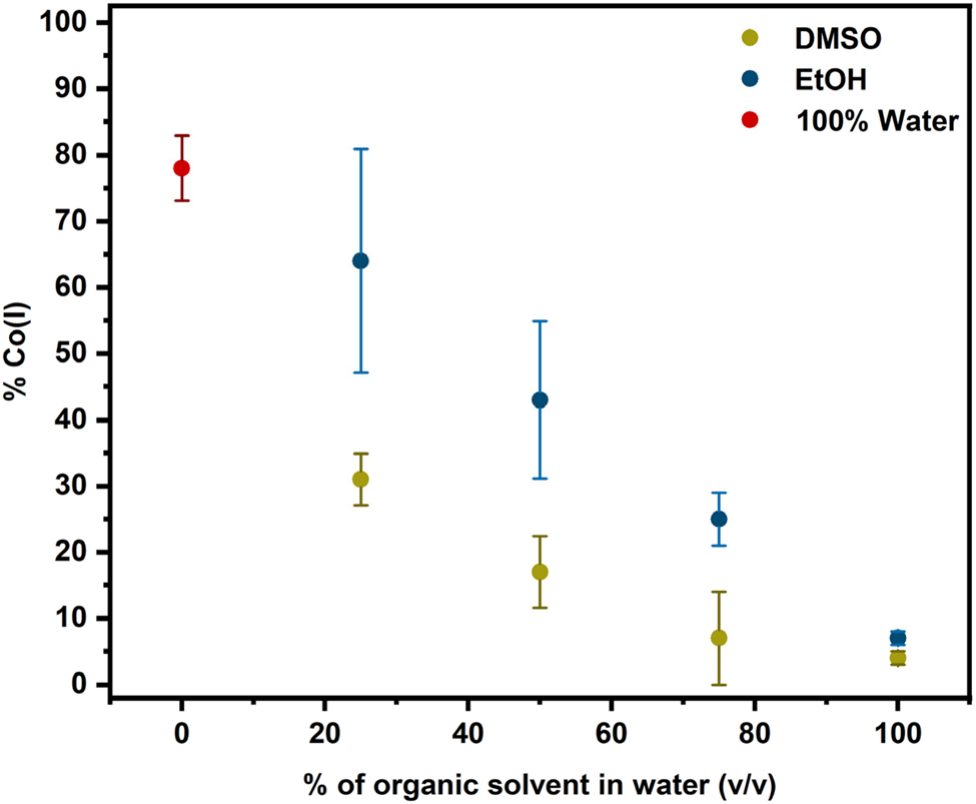
Decrease in percent concentration of Co(i) with increasing percentage (v/v) of DMSO and EtOH in water during photolysis of PhHxCbl. The estimated error is within 10%. All data points were collected in triplicate, except for the 25% EtOH sample, which was analyzed in duplicate.

To investigate the solvent dependence further, we conducted photolysis experiments with DMSO/water mixtures. Since our data in Fig. 3 and also ultrafast spectroscopy have shown that Co(ii) is the immediate first product of photolysis of alkylCbls, we wondered whether DMSO might be simply acting as a radical quencher—preventing formation of Co(i) from the initially formed [Co(ii) + R˙]. To test this hypothesis, we investigated product formation after photolyzing alkylCbls in the presence of increasing amounts of DMSO. Photolysis in DMSO/water mixtures, or in the presence of known radical traps such as TEMPO and DMPO in water, causes a change from Co(i) to the Co(ii) product (Fig. 5, S11, and S32–S33). The percentage of the Co(i) species decreases as the percentage of DMSO increases. We observe a similar trend with EtOH, although EtOH seems to be slightly less effective than DMSO at shifting the product towards Co(ii) (Fig. S22 and S23). EPR data provides independent confirmation that Co(ii) is formed when alkylCbls are photolyzed in DMSO and 50 : 50 ethanol/water and matches well with previously reported data (Fig. S50).^61,62^

### 3.4 Solvent choice influences the organic product, with water favoring alkene formation and ethanol favoring alkane formation

Aside from the reduced cobalamin, the other product of photolysis of alkylCbls is an organic fragment derived from the alkyl ligand. In addition to reducing Co(ii), this radical could potentially dimerize or undergo disproportionation producing an alkene and an alkane.^63,64^ Alternatively, the radical could react with the solvent either donating an H atom, yielding alkene or accepting an H atom, generating an alkane. For photolysis at our low concentrations, bimolecular reactions of two radicals are comparatively less probable, and we have found no evidence for dimer formation. In order to explore how solvents affect the alkyl radical species, we investigated and compared the organic products that were formed during photolysis of alkylCbls in solvents including water, DMSO, EtOH, and MeOH.

**Fig. 6:**
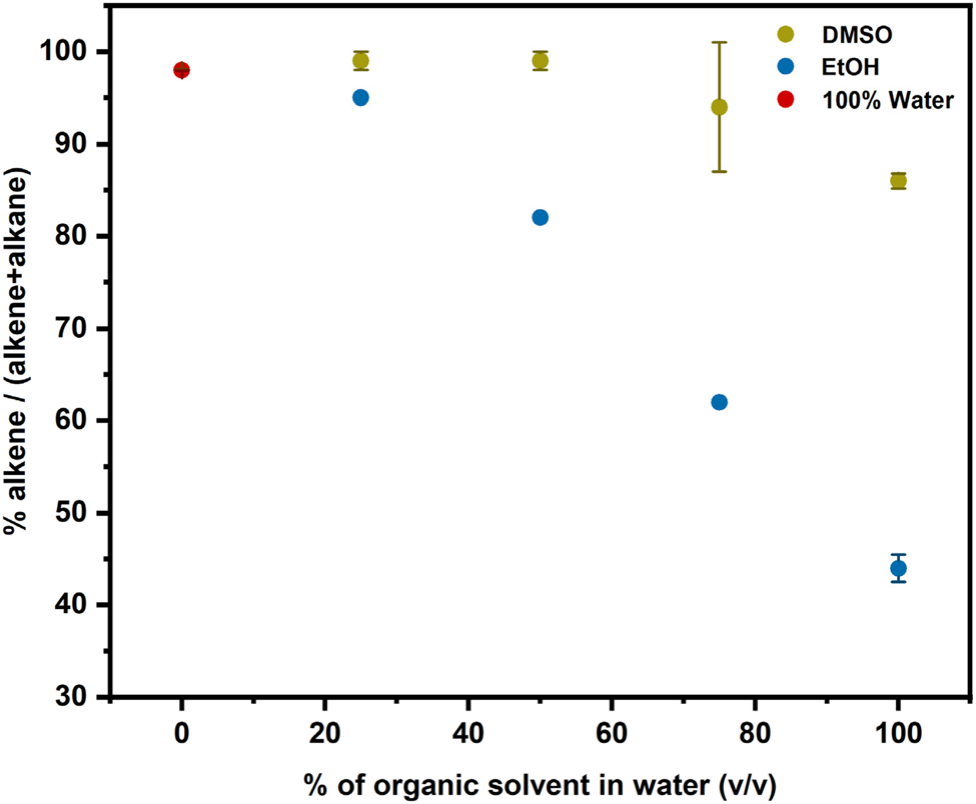
Ratio of alkene to total organic product for the photolysis of PhHxCbl in different solvent mixtures as determined by GC-MS.

When PhHxCbl is photolyzed in 100% water, the predominant product is the alkene (∼98%) (see Table S4). When the photolysis is carried out in water/DMSO mixtures, there is a small decrease in the fraction of alkene, but it still remains the major product (Fig. 6 and Table S4). Ethanol shows a gradual decrease in the alkene fraction, and 100% EtOH gives majority conversion to the alkane (Fig. 6, and Table S5).

Across the spectrum of different solvents, the trend in alkene yield is as follows: water > DMSO > MeOH > EtOH (representative data shown in Fig. S54, and Tables S4, S5). Notably, both EtOH and DMSO prevent Co(i) formation, but increasing EtOH content favors alkane production while increasing DMSO content does not, indicating that the transition from Co(i) to Co(ii) does not correlate with increased alkane formation. Both EtOH and MeOH are known to be strong hydrogen atom donors, so in principle they could quench the alkyl radical. Photolysis in ethanol-*d*_1_ showed no evidence for incorporation of deuterium into the alkane product. However, we do observe deuterium incorporation into the alkane product when the photolysis is carried out in either ethanol-*d*_6_ or methanol-*d*_4_. The fraction of the alkane product decreases in deuterated solvent (Table S8) perhaps due to a kinetic isotope effect.

It has been reported that the presence of sodium bicarbonate (NaHCO_3_) in acetonitrile is essential for the selective formation of alkene products rather than alkane products *via* photochemistry in a CNCbl based system.^38^ The insolubility of our alkylCbls and also CNCbl in acetonitrile prevented us from studying single turnover conditions for this reactivity in our system. However, in our tested solvents, we find that under single turnover conditions, NaHCO_3_ does not have any detectable effect on either the reaction rate, as measured by the loss of the Co(iii) reactant, or product selectivity (Fig. S10, S14, S18–S19, S25, S27, and Table S6). It may be that in acetonitrile with excess NaBH_4_ present, the NaHCO_3_ is important not for deprotonating a putative H–Co(iii) species as previously suggested,^38^ but rather for removing the β-H from the alkyl radical.

### 3.5 Mechanistic studies into the formation of alkylcobalamins

The common way to generate alkylCbls is by reaction of a cob(III)alamin, for example CNCbl or HOCbl, with an alkyl halide in the presence of a large excess of NaBH_4_ as the reductant.^39,52,65^ Here, it is believed that the reductant first generates the Co(i) form of cobalamin, which is considered a supernucleophile, and which is presumed to then react with the alkyl halide by oxidative addition to generate the alkylCbl. Since alkylCbls are not reducible by NaBH_4_, this leads to quantitative product formation. However, actual mechanistic information about this reaction is sparse, since the cob(I)alamin is typically formed by using a large excess of the chemical reductant^38,66^ or *via* electrochemistry.^56^ As shown above, we have been able to use single-turnover photolysis of alkylCbls in water to cleanly generate cob(I)alamin without the need for an external reductant. This provides a unique opportunity for us to investigate the reaction of cob(I)alamin with alkyl halides in single turnover experiments and to obtain mechanistic insight into this reaction. When PrCbl is photolyzed in a saturated stock solution of 1-bromopropane (PrBr) in water (∼20 mM) to generate Co(i) *in situ*, we obtained primarily a mixture of HOCbl and H_2_OCbl (Fig. S40, S41b, and Table S3). Photolysis of the longer alkyl chain PhHxCbl in the presence of excess alkyl halide yielded the same observations as seen for PrCbl (Fig. S44). These observations led us to further investigate the mechanism of the reaction between Co(i) and an alkyl halide by adding PrBr to pre-formed Co(i) rather than photolyzing in the presence of PrBr.

**Fig. 7:**
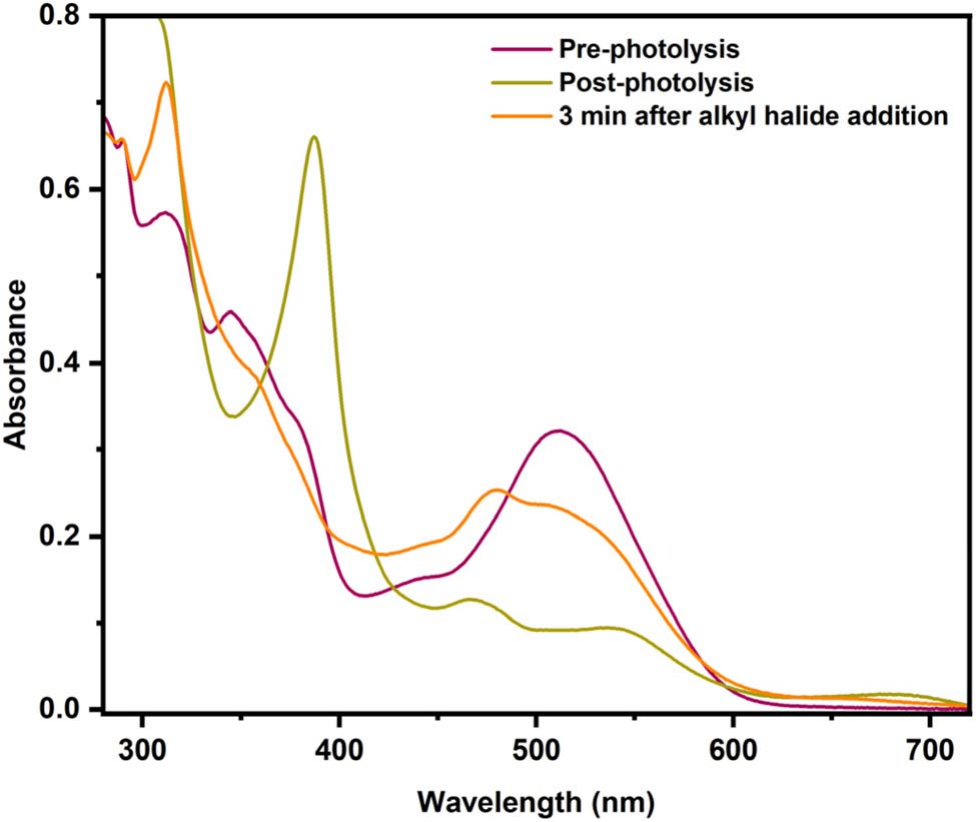
UV-Vis absorption spectra showing formation of the Co(i) species for a PrCbl sample in water followed by the addition of excess 1-bromopropane (making a saturated solution of the alkyl halide, ∼20 mM), showing formation of a Co(ii) species and PrCbl after reacting for 3 min in the dark.

Since 1-bromopropane has limited solubility in water, the addition of excess (*ca.* 100 µL) PrBr to water gives a saturated solution, with a PrBr concentration of ∼20 mM. When 100 µL of PrBr is added to photolytically pre-formed Co(i) in the dark, we obtain an ∼1 : 1 mixture of Co(ii) and the corresponding alkylCbl complex (PrCbl) (Fig. 7). A minor percentage of H_2_OCbl/HOCbl (∼5%) was also observed. The fact that the addition of RBr to the photogenerated Co(i) species gives a mixture of Co(ii) and RCbl suggests a branching pathway.

To understand the mechanism behind formation of the Co(ii) product upon adding the alkyl halide to the Co(i) species (generated by photolysis), we performed experiments using different concentrations of 1-bromopropane. One explanation for the formation of Co(ii) is that Co(i) can react with RBr to form either RCbl + Br^−^ or Co(ii) + (R˙ + Br^−^).^67^ In this case, the ratio of RCbl : Co(ii) should be independent of the concentration of RCbl. Alternatively, if the branching was between formation of RCbl + Br^−^ and formation of R^−^ + BrCbl(III) (which would likely undergo ligand exchange to give H_2_OCbl(III)), the resulting Co(iii) species would be expected to undergo rapid comproportionation with Co(i) to generate the observed Co(ii) product.^67,68^ In this case, the ratio of PrCbl to Co(ii) should increase as the concentration of RBr increases, since this will increase the likelihood that Co(i) reacts with RBr rather than with Co(iii). As shown in Table 1, we added to a 0.03 mM PrCbl solution (a) 100 µL of degassed 1-bromopropane (making the solution saturated with alkyl halide, ∼20 mM) (Fig. 7 and S41d), (b) 100 µL of a 30 mM stock solution of bromopropane (final PrBr concentration: 1.5 mM; 50 equivalents) (Fig. S42), and (c) 50 µL of a 30 mM stock solution of bromopropane (final PrBr concentration: 0.75 mM; 25 equivalents) (Fig. S43). We found that the ratio PrCbl : Co(ii) decreases at lower RBr concentrations, consistent with the Co(ii) that we observe having been formed through comproportionation. We confirmed the feasibility of comproportionation by photolyzing PrCbl in the presence of an equivalent amount of H_2_OCbl; the only observed product was Co(ii) with no indication of Co(i). This demonstrates that comproportionation occurs rapidly, as illustrated in Fig. S48.

**Table 1:** Fits obtained for PrCbl photolysis in water followed by addition of large excess (saturated solution; ∼20 mM), 50 equivalents, and 25 equivalents of 1-bromopropane (equivalents are relative to PrCbl). The standard deviation in ΔPrCbl/|ΔCo(i)| for ∼20 mM is 2%, 1.5 mM is 2%, and for 0.75% mM it is 2.8%

Sample	% Co(i)	% Co(ii)	% PrCbl	% H_2_OCbl	% HOCbl	|ΔCo(i)|	ΔCo(ii)	ΔPrCbl	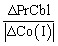
**∼20 mM**									
Pre-photolysis	4	7	89	0	0				
Post-photolysis	89	7	4	0	0				
+ RBr + 3 minutes	1	48	46	0	5	88	41	42	49%

**1.5 mM (50 eq.)**									
Pre-photolysis	5	8	87	0	0				
Post-photolysis	80	14	6	0	0				
+ RBr + 3 minutes	5	65	29	0	0	75	51	23	33%

**0.75 mM (25 eq.)**									
Pre-photolysis	3	5	92	0	0				
Post-photolysis	93	3	3	0	0				
+ RBr + 3 minutes	40	44	16	0	0	53	41	13	26%

**Fig. 8:**
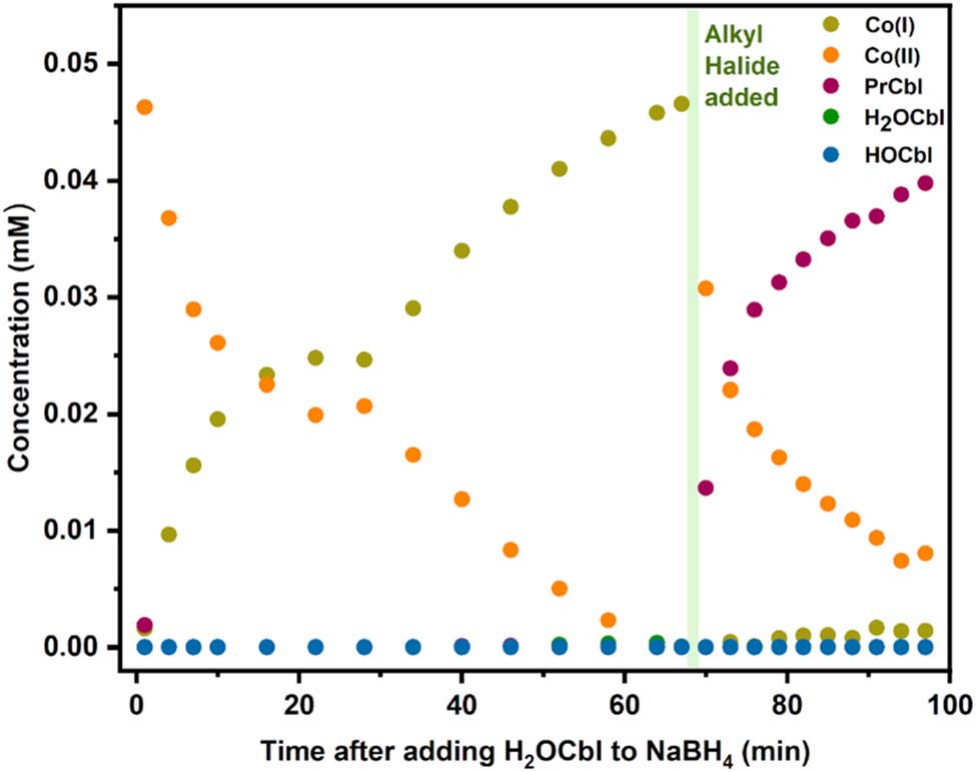
Fitted concentrations of different cobalamin species over time showing immediate formation of Co(ii) upon adding H_2_OCbl to NaBH_4_, followed by the slow reduction to Co(i), and alkyl halide addition, finally generating PrCbl.

We also investigated the standard reaction conditions applied for the synthesis of alkylCbls, where cob(III)alamin is reduced with excess NaBH_4_ to generate Co(i), followed by the addition of alkyl halide. We observe that CNCbl initially reacts with NaBH_4_ to form Co(ii), followed by gradual formation of Co(i) (Fig. S49). When excess 1-bromopropane is added, giving a saturated solution of alkyl halide, we initially observe Co(ii) formation along with some alkylCbl, followed by gradual consumption of Co(ii) and complete formation of alkylCbl (Fig. S49). We confirmed that Co(ii) is inert towards 1-bromopropane by photolyzing PrCbl solution in water in the presence of both a radical scavenger, DMPO, and 1-bromopropane. We also photolyzed the alkylCbl independently with DMPO present followed by addition of alkyl halide afterwards. In all cases, only Co(ii) was formed, confirming that alkyl halides do not react directly with Co(ii) (Fig. S45–S47). For further clarification as to why, under synthetic conditions, cobalamin predominantly shows up as Co(ii) immediately upon adding the alkyl halide, we then conducted an experiment adding NaBH_4_ to an aqueous solution of H_2_OCbl, which is the other plausible product of the reaction between Co(i) and alkyl halide alongside RCbl, due to the branching pathway. This led to the rapid formation of Co(ii) (within the mixing time of the addition), followed by a slower reduction to Co(i) (Fig. 8). This indicates that NaBH_4_ initially leads to the rapid reduction of H_2_OCbl to Co(ii) through a one-electron reduction process. This is followed by a slower one-electron reduction to Co(i), which in turn reacts rapidly with RBr. Therefore, in the presence of excess NaBH_4_, one would expect to observe significant cob(II)alamin which would gradually convert to the non-reducible alkylCbl (*via* Co(i)) as seen in Fig. 8. Despite the possibility of a two-electron reduction reaction, the fast first step and the slower second step explain the prevalence of Co(ii) during alkylCbl synthesis.

## 4 Discussion

In this study, we have explored the photochemistry of alkylcobalamins on the milliseconds to seconds time scale, identifying both the cobalt species and the corresponding organic products that are formed in different solvents. Ultrafast UV-visible transient absorption spectroscopy has shown that on the picosecond time scale, photolysis of the Co(iii)–R bond leads to the formation of cob(II)alamin and the organic radical, R˙, which may either recombine to reform the starting alkylcobalamin or undergo cage escape.^11,13,14^ Our work shows that when the photolysis is performed in water, the ultimate products are Co(i) and a primary alkene, consistent with loss of the β-hydrogen from the radical. This is reminiscent of work by Leys and co-workers showing the formation of Co(i) and an alkene product following photolysis of AdoCbl bound to the cobalamin binding domain of *Thermus thermophilus* CarH protein.^40^ However, our data demonstrate that the protein scaffold is not necessarily required for the generation of Co(i) and that, depending on the alkyl group, the protein is also not crucial for generating an alkene product. Rather, both seem to be fundamental properties of alkylCbls when photolyzed in water.

**Fig. 9:**
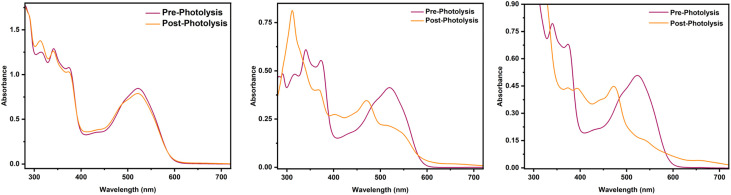
Photolysis of MeCbl (from left to right) in water, DMSO, and EtOH under anaerobic conditions.

We envision the mechanism of the process in water as follows: the initial photolysis generates Co(ii) and an alkyl radical. If there is nothing available to quench the radical, it can readily lose the weak β-H to give the alkene product. In water, which is not a good H-atom acceptor, the Co(ii) itself can act as a β-H acceptor, ultimately resulting in reduction of the cobalamin to Co(i). This could occur *via* a proton-coupled electron-transfer (PCET) mechanism, where the Co(ii) center is reduced, and a proton is released into the solvent. Similar chemistry has been observed in Cu(ii) complexes.^69^ Consistent with this model, methylcobalamin, which lacks the β-H, shows only limited photochemistry in water under anaerobic conditions, even after extensive photolysis (Fig. 9). This is similar behavior as observed for neopentylcobalamin, where the neopentyl radical, which also lacks a β-H, shows slow photochemistry in water under anaerobic conditions.^70^ Our finding that protons are released concomitant with Co(i) formation and that a radical trap prevents Co(i) formation are both consistent with a model in which a proton and an electron are both released during the formation of Co(i) following photolysis of alkylCbls in aqueous solutions.

In contrast to the behavior in water, photolysis of alkylCbls in the organic solvents DMSO, EtOH and MeOH generate Co(ii) rather than Co(i). It may be that the radical reacts with the solvent before it is able to donate the β-H to Co(ii). In DMSO, the alkene remains the main organic product. This is consistent with a study by DiLabio and coworkers,^71^ finding that DMSO behaves as a hydrogen atom acceptor in reactions with the benzyloxyl radical (C_6_H_5_CH_2_O˙) in a process that involves proton-coupled electron transfer and ultimately generates DMS and a hydroxyl radical. Similar reactivity following the photolysis of alkylcobalamins would give alkene and a DMSO radical while leaving the cobalamin in the Co(ii) oxidation state. It has been suggested previously that alcohols could serve as H-atom donors to the alkyl radical^8,72^ and our deuterium isotope experiments support this notion. Not only do we see deuterium incorporation into the alkane, but in addition we see a decrease in alkane formation, consistent with a kinetic isotope effect.

**Fig. 10:**
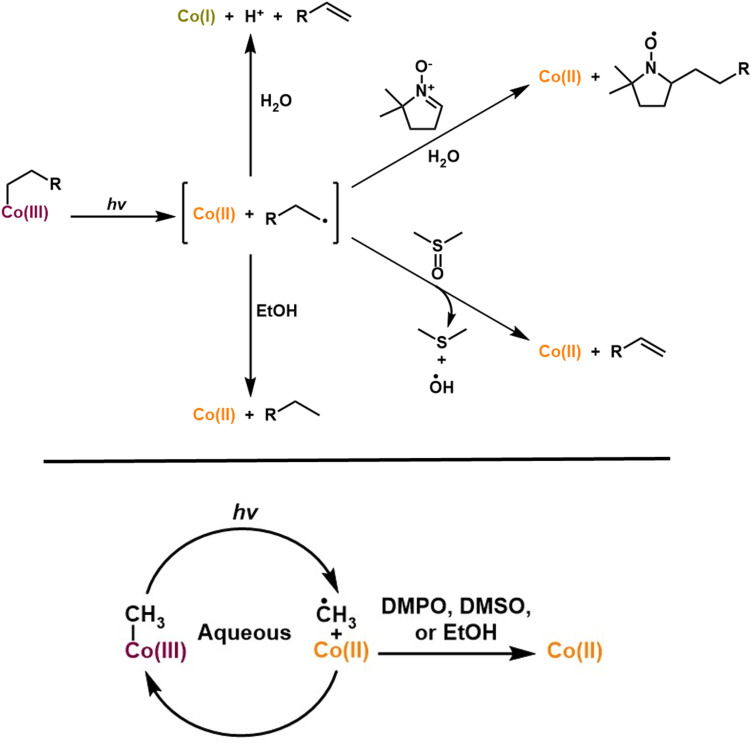
Proposed mechanism for photolysis of alkylCbls under different conditions.

This proposal is consistent with the observation that MeCbl, which is stable to photolysis in water, can be made to form Co(ii) either by adding a known radical trap (TEMPO) (Fig. S33) or by switching to DMSO or EtOH as the solvent (Fig. 9). Neopentylcobalamin similarly shows formation of Co(ii) in the presence of 2-propanol,^70^ and early studies of MeCbl photolysis showed that longer-chain alcohols, which are more sensitive to radical mediated attack, promote the photolysis of MeCbl.^8^ In this model, if there is a pathway to quench the transient methyl radical Co(ii) will form; only in water, where such quenching is not possible, is MeCbl stable to photolysis. This trend was further corroborated in subsequent studies involving ethylcobalamin and *n*-alkylcobalamin, where increasing the proportion of alcohol in aqueous solution similarly enhanced the photolysis rate and stabilized Co(ii).^72^ The proposed mechanism for alkylCbl photolysis in various solvents is summarized in Fig. 10.

Work by West and coworkers previously found that photochemical alkene formation from alkylCbls was only seen in the presence of sodium bicarbonate.^38^ In contrast, we find that the organic product that is formed after photolyzing alkylCbls is an alkene as long as (a) there is a β-H present in the substrate/alkyl radical, and (b) there is nothing in solution that can intercept the radical or the β-H prior to cobalt reduction. In our homogeneous solutions, sodium bicarbonate has no effect on the organic product, the cobalt oxidation state, or the reaction rate. It may be that the requirement for bicarbonate in West's reaction is related to the solvent (acetonitrile) since alkylCbls are insoluble in acetonitrile, thus giving a heterogeneous mixture, unlike our homogenous system. It has been speculated that cobalamin reactivity involves a Co(iii)-hydride species.^10,38^ We do not see any evidence for such a species and believe that our data are better explained by the loss of an H-atom (or proton coupled electron transfer) from the organic radical in water. Consistent with this, Co(i) formation is not required for alkene formation if there is a solvent such as DMSO that can act as the H-atom acceptor. As a final note, our results show that alkylCbl photolysis to give alkenes is a fast process in contrast with the slow, multi-hour timescale found in West's system. It may be that what ultimately limits the turnover frequency in the latter is the very low solubility of cobalamin in acetonitrile, which greatly hinders the reformation of the alkylCbl starting material *via* the chemical reductant after a turnover is finished. In this way, our results not only provide insight into the biologically relevant photochemistry of alkylCbls but also provide mechanistic clues for the development of improved catalytic reactions in organic chemistry that are based on alkylCbl photochemistry.

**Fig. 11:**
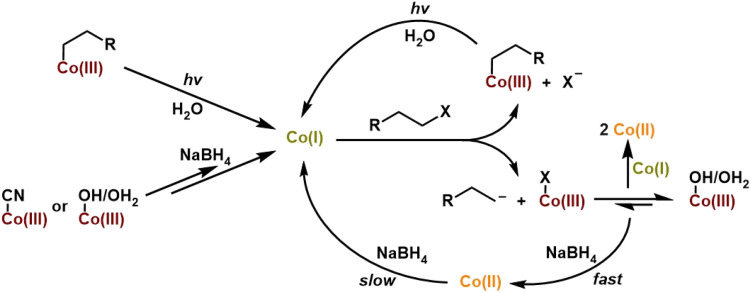
Proposed branching pathway for the reaction of Co(i) with alkyl halide.

Finally, we investigated the mechanism of alkylCbl formation from the Co(i) form of cobalamin and an alkyl halide. In the literature, the Co(i) form is often referred to as a supernucleophile, implying that this reaction is a simple oxidative addition, leading to the clean formation of the alkylCbl. However, we find that, at least in water, the reaction Co(i) + RBr → Co(iii)–R is not the only reaction. Instead, we observe that one of the major products is a Co(ii) species together with some alkylCbl, with the fraction of alkylCbl increasing to half of the cobalamin product at the highest RBr concentrations. This is consistent with the reaction of Co(i) with alkyl halide having a branching point: one path forms alkylCbl (RCbl) while the other gives Br–Co(iii) or H_2_OCbl, which undergoes rapid comproportionation in the presence of Co(i) (see Fig. 11). This quick reaction between excess Co(i) and Co(iii) produces Co(ii) and hinders complete formation of RCbl unless there is excess reductant present. The observation of an approximately 1 : 3 ratio of RCbl to Co(ii) at the lowest RBr concentrations (0.75 mM) suggests a branching ratio of 40% RCbl + Br^−^ and 60% BrCbl + R^−^, taking into consideration the stoichiometry involved (2 Co(i) + RBr → 2 Co(ii) in the latter scenario). If Co(i) is generated *in situ via* photolysis, the steady-state concentration of Co(i) is too low to give significant comproportionation, and we instead observe a mixture of H_2_OCbl and HOCbl as the only cobalamin products (any RCbl that is formed will be rephotolyzed and thus does not accumulate). In the presence of NaBH_4_, HOCbl/H_2_OCbl is rapidly reduced to Co(ii), followed by slower formation of Co(i). This Co(i) can then react with an additional alkyl halide, and over multiple such cycles, the alkylCbl product accumulates. Since NaBH_4_ cannot reduce the alkylCbl product, once it is formed it will accumulate. This reaction somewhat mirrors the previously reported dehalogenation reactions by Co(i) in vitamin B_12_ and similar complexes.^38,45,50,51,56,66,73,74^

It is intriguing that HOCbl/H_2_OCbl is formed as the major product through illumination of alkylCbl in the presence of saturated concentrations of alkyl halide, while we observe almost complete formation of alkylCbl in the dark reaction using chemically reduced CNCbl or H_2_OCbl with NaBH_4_. These processes represent two different facets of an intersecting network of cycles. In the illuminated scenario, a mixture of H_2_OCbl and HOCbl serves as a terminal state because these species are not photoactive—once formed, it remains trapped; any alkylCbl produced can undergo further photolysis, continuing to cycle until complete conversion is achieved. If Co(i) is generated solely through photolysis and RBr is present at a relatively high concentration, the steady-state concentration of Co(i) will remain low, thereby preventing significant comproportionation of Co(iii); hence primarily Co(iii) accumulates. Conversely, in the dark reaction using NaBH_4_, alkylCbl becomes the terminal state as it is not reducible by the chemical reductant. With excess reductant present, any Co(iii) other than the alkylCbl that is formed will be reduced back to Co(i), eventually leading to the accumulation of alkylCbl, since it cannot be further reduced. These observations align with the mechanism outlined in Fig. 11.

## 5 Conclusions

Our observations are important for understanding both the photochemical and enzymatic reactivity of cobalamins. Photolysis of alkylCbls in water rapidly generates a Co(i) species, accompanied by the transformation of the initially formed alkyl radical into the alkene. Although Co(ii) is the initial photolytic product, the final product of the reaction in water is Co(i), presumably as a result of H-atom transfer. Notably, this occurs independent of a protein environment. Time-resolved spectroscopy and mass spectrometry reveal that both the final cobalt oxidation state and the organic product distribution are strongly influenced by the solvent environment. Although the nature of the organic product depends on the hydrogen atom donor or acceptor properties of the organic solvent, it is not directly linked to the final Co oxidation state and can be better rationalized by radical chemistry occurring upon photolysis. With a system for generating cob(I)alamin photochemically, without the need for chemical reductants, we were able to investigate its reactivity with alkyl bromides, directly observing two mechanistic pathways: formation of Co(iii)-alkylcobalamin and a Co(ii) product, likely through comproportionation between Co(i) and Co(iii)-halide. This mechanistic branching, typically masked by excess reductant, not only broadens our understanding of cobalamin photochemistry but also has implications for the mechanisms of B_12_-dependent dehalogenases utilized in bacterial bioremediation. Overall, our results highlight the versatility of light-mediated cobalamin chemistry, expanding its relevance beyond bacterial physiology to inform new directions in gene regulation, biocatalysis, synthetic organic chemistry, and the remediation of halogenated environmental contaminants.

## Author contributions

The manuscript was written through contributions of all authors, who all have given approval to the final version of the manuscript.

## Conflicts of interest

There are no conflicts to declare.

## Supplementary Material

SC-017-D5SC07054F-s001

## Data Availability

The data supporting this article have been included as part of the supplementary information (SI). Supplementary information is available. See DOI: https://doi.org/10.1039/d5sc07054f.
