# Supplementary material for: Shining light on the mechanism of photochemical alkene formation in vitamin B12
Source: Chem Sci. 2026 Jan 23;17(11):5518–31. doi: 10.1039/d5sc07054f (PMC12828988; doi:10.1039/d5sc07054f)
Supplement: SC-017-D5SC07054F-s001 [file SC-017-D5SC07054F-s001.pdf]

Supplementary Information (SI) for Chemical Science.
This journal is © The Royal Society of Chemistry 2026

**Supporting Information**

# Shining Light on the Mechanism of Photochemical Alkene Formation in Vitamin $B_{12}$

Alivia Mukherjee[a], Summer Y. Wu[a], David J. Cooper[a,b], Rachel Hendrickson[b], Roseanne J. Sension[a,c], Nicolai Lehnert[a,b]*, James E. Penner-Hahn[a,b]*

a. Department of Chemistry, University of Michigan, Ann Arbor, Michigan 48109-1055, United States.

b. Department of Biophysics, University of Michigan, Ann Arbor, Michigan 48109-1055, United States.

c. Department of Physics, University of Michigan, Ann Arbor, Michigan 48109-1055, United States.

Correspondence:

jeph@umich.edu

lehnertn@umich.edu

**Table of Contents**

**Shining Light on the Mechanism of Photochemical Alkene Formation in Vitamin $B_{12}$ .................. 1**

**1. General Considerations: .................................................................................................. 3**

**2. Synthesis of Phenylhexylcobalamin, Naphthylethylcobalamin and Propylcobalamin: ......... 5**

Figures S1-S3

**3. Setup for Photolysis Experiments: ................................................................................. 8**

Figure S4

**4. Fitting Procedure: .......................................................................................................... 9**

Figures S5-S8

**5. Steady State and Time-Resolved Spectral Measurements: ................................................ 12**

Figures S9-S38

**6. Mechanistic studies into the formation of alkylcobalamins: ............................................. 42**

Figures S39-S49

Tables S1-S3

**7. EPR Measurements: ....................................................................................................... 52**

Figures S50

**8. GC-MS Analysis: ............................................................................................................ 53**

Figures S51-S56

Tables S4-S6

**9. Reduction mechanism: ................................................................................................... 62**

Figures S57-S60

Table S7-S8

**10. References: ................................................................................................................. 65**

## 1. General Considerations:

All solvents and commercially available reagents were purchased from commercial sources and used as received without further purification unless noted otherwise. Methyl sulfoxide (DMSO) was either purchased as ACS grade from Fisher and sparged with dinitrogen ($N_2$) overnight to remove dioxygen ($O_2$) or was purchased as extra dry, 99.7%, in an AcroSeal bottle and used as received. Extra dry methanol (MeOH) (Acroseal$^{TM}$) was obtained from Fisher. Ethanol (99.5%, anhydrous) was purchased from Sigma-Aldrich in a Sure-Seal bottle and used as received. Ethanol-$d_6$ (D, 99%) and Methanol-$d_4$ (D, 99.8%), anhydrous, was purchased from Cambridge Isotope Laboratories and sparged with $N_2$ before use. Milli-Q water was sparged overnight with $N_2$ before use and/or freeze-pump-thawed through multiple vacuum-freeze cycles.

Cyanocobalamin (CNCbl) was obtained from Santa Cruz Biotechnology. Sodium borohydride ($NaBH_4$), sodium bicarbonate ($NaHCO_3$), 1-bromopropane, and 1-bromo-6-phenylhexane were purchased from Fisher, and 1-(2-bromoethyl)naphthalene was purchased from Sigma-Aldrich. The standards for GC-MS experiments, 6-phenyl-1-hexene (98%, stabilized with tert-butyl catechol) and 1-vinylnaphthalene (95%, stabilized with tert-butyl catechol), were obtained from Ambeed and Fisher Scientific, respectively. 5,5-dimethyl-1-pyrroline-N-oxide (DMPO) and 2,2,6,6-tetramethyl-piperidin-1yl-oxyl (TEMPO) were from Sigma-Aldrich. Methylcobalamin (MeCbl) and hydroxocobalamin hydrochloride (HOCbl•HCl) were obtained from Ambeed and Sigma-Aldrich respectively.

### Sample preparation for photolysis

Photolysis experiments were performed at room temperature in a modified anaerobic quartz cuvette with a sealable Teflon valve connected to a side arm opening. The side arm with a 14/20 neck was further secured with a rubber septum with electrical tape/copper wire to prevent air from entering. Unless stated otherwise, all samples (UV-Vis: between 0.015 to 0.06 mM; GC-MS: between 0.15 mM to 0.6 mM) were prepared under $N_2$ either using standard Schlenk techniques or inside a glove box under red light to prevent inadvertent photoconversion of the samples. Stock solutions were sparged thoroughly with $N_2$ then transferred to the cuvette and sparged further. A positive-pressure $N_2$ atmosphere was maintained in the cuvette to prevent $O_2$ contamination. Steady state UV-Vis absorption spectra were measured using a dual beam Shimadzu UV-Visible spectrophotometer (Shimadzu UV-2600 spectrometer) using a 1 cm path length quartz cuvette. The spectrum of pure solvent was measured and background subtracted.

Time-resolved spectra were measured using an Avantes fiber optic system (AvaSpec-2048-USB2-UA) collecting the transmittance data from millisecond to second timescale.

Samples were prepared and photolyzed under anaerobic conditions as described above. After photolysis, a small-scale liquid-liquid extraction was performed to extract the organic photoproduct(s) into hexanes. Samples photolyzed in ethanol or methanol were passed through a small silica column to collect the organic product. The extracted product solution samples were then analyzed using a quadrupole mass spectrometer capable of unit mass resolution coupled with a gas chromatograph. Gas chromatography-mass spectrometry (GC-MS) analyses were performed using a Shimadzu QP-2010 GC-MS equipped with a 30 m long DB-5 column with 0.25 mm ID. Separation method: 2 μL injection, injection temperature: 200°C, detector temperature: 325°C. Gradient: column oven temperature set to 65°C for 3 min, then to 250°C at 20°C $min^{-1}$ and held at 250°C for 2.5 min. As the alkene and alkane peaks are not completely resolved, relative concentrations were determined by integrating the parent peak, and scaling this to the total ion count for each compound.

**GC program with separation method:** Injection Volume: 20 μL; Start time: 4.00 min End time: 14.75 min; Pressure: 6.6 psi; Total flow: 20.5 mL/min; Column flow: 0.83 mL/min; Linear velocity: 33.4 cm/s; Purge flow: 3.0 mL/min; Split ratio: 20.0; Scan speed: 769; Start m/z: 40.00; End m/z: 400.00; Ion Source Temp: 200.00°C; Interface Temp: 250.00°C; Solvent Cut Time: 2.50 min; Detector Gain Mode: Absolute; Detector Gain :1.50 kV; Threshold: 1000; Column Oven Temp: 65.0°C; Injection Temp: 200.00°C; Injection Mode: Split less; Sampling Time: 2.00 min; Flow Control Mode: Pressure; High Pressure Injection: OFF; Carrier Gas Saver: OFF

**Oven Temp. Program**

| Rate | Temperature (°C) | Hold Time (min) |
|---|---|---|
| - | 65.0 | 3.00 |
| 20.00 | 250.0 | 2.50 |
| 40.00 | 325.0 | 5.00 |
| | | |

## 2. *Synthesis of Phenylhexylcobalamin, Naphthylethylcobalamin and Propylcobalamin:*

The synthesis and work up procedure for these alkylcobalamins was performed in a dark room under red light. Cyanocobalamin (CNCbl) (50 mg, 37 μmol) in 5 mL of Milli-Q water was reduced with ca. 30 mg (0.8 mmol) of $NaBH_4$ under anaerobic conditions. After 90 minutes of stirring at room temperature under an $N_2$ atmosphere, ~75 μL (89 mg, 0.37 mmol) of 1-bromo-6-phenylhexane or 50 μL (83 mg, 0.35 mmol) of 1-(2-bromoethyl) naphthalene were dissolved in 500 μL of EtOH then added to the reaction mixture. For *n*-propylcobalamin (PrCbl), ~800 μL of 1-bromopropane was directly added. The reaction was terminated after overnight stirring by adding 5 mL of 30% (v/v) acetone. The quenched reaction mixture was separated by liquid-liquid extraction of the aqueous mixture with a 50/50 (w/v) solution of phenol/dichloromethane. Diethyl ether was then added to the organic solution to precipitate the product, and the solution was stored at −20°C overnight. The product was filtered and collected on a fine frit, washed with diethyl ether (100-200 mL) and dried with a stream of nitrogen. The absorption spectra of phenylhexylcobalamin (PhHxCbl) and naphthylethylcobalamin (NapEtCbl) are very similar to PrCbl. PhHxCbl: 41.5 mg (83%) yield; $\lambda_{\alpha\beta}$ = 511 nm in $H_2O$ (Figure S1); m/z = 1490.7 (Figure S2). NapEtCbl: 25 mg (50%) yield; $\lambda_{\alpha\beta}$ = 521 nm in $H_2O$ (Figure S1); m/z = 1484.6 (Figure S3). The reported yields represent the mean values calculated from no fewer than three separate batches. $\lambda_{\alpha\beta}$ represents the wavelength of maximum absorbance for the αβ band of the respective alkylcobalamins. Sample purity was determined by UV-visible spectroscopy. Successful synthesis of the alkylCbls is indicated by a blue shift of the CNCbl starting material αβ band and the loss in intensity of the γ band.

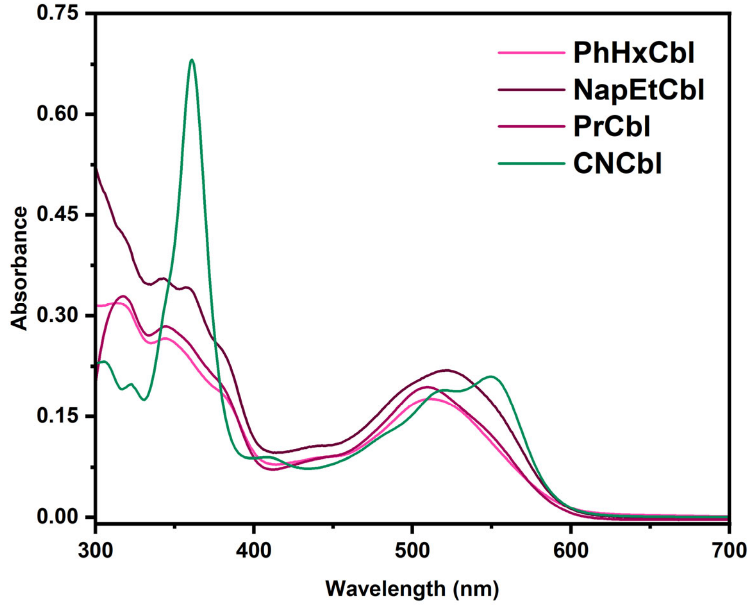


**Figure S1.** UV-Vis absorption spectra of PhHxCbl, NapEtCbl, PrCbl, and CNCbl in water.

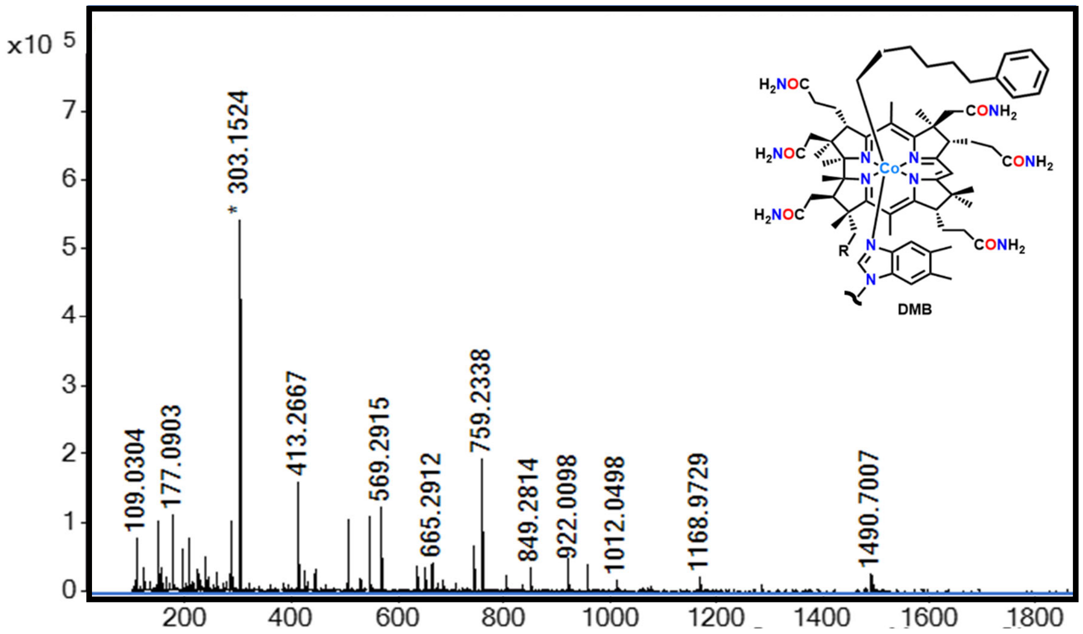


**Figure S2.** Mass spectrum of PhHxCbl with the parent peak represented at m/z of 1490.7.

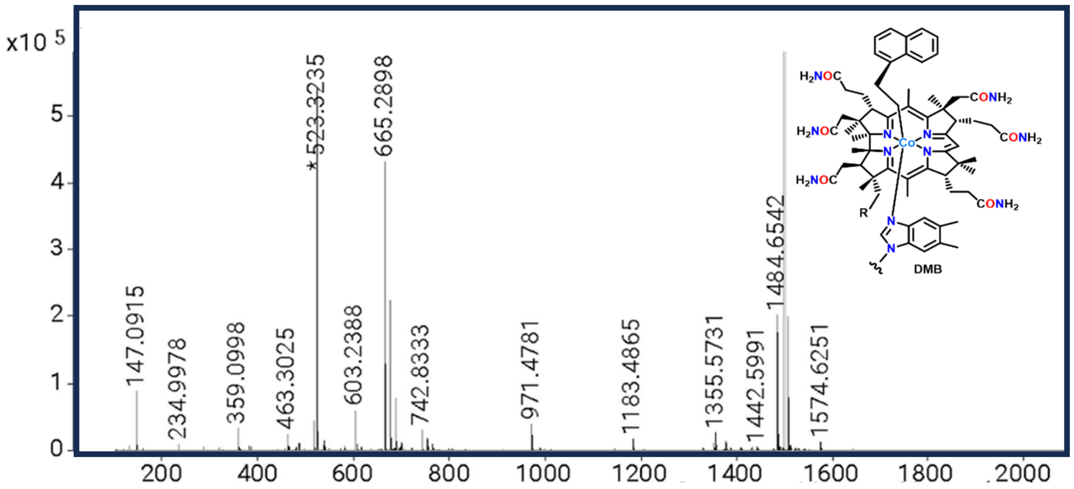


**Figure S3.** Mass spectrum of NapEtCbl with the parent peak represented at m/z of 1484.65.

### *3. Setup for Photolysis Experiments:*

Time-resolved spectra were measured using an Avantes fiber optic system to collect the data from the millisecond to second timescale. A white Xenon lamp was used to perform all the photolysis experiments. A glass UV filter was employed to restrict the light source to the visible range. This approach was necessary as cobalamin photochemistry relies on specific wavelengths.[1-4] A setup inspired by laser pump-probe technique was devised for these continuous wave experiments, utilizing the white light Xenon lamp for both photolytic excitation and probing purposes (Figure S4). A beam splitter was used to divide the white light beam into a 96:4 ratio. The probe light, a broadband continuum spanning 300 nm to 750 nm, was generated by focusing the 4% portion of the split light using focusing mirrors and lenses. Light intensity was regulated using a neutral density filter.

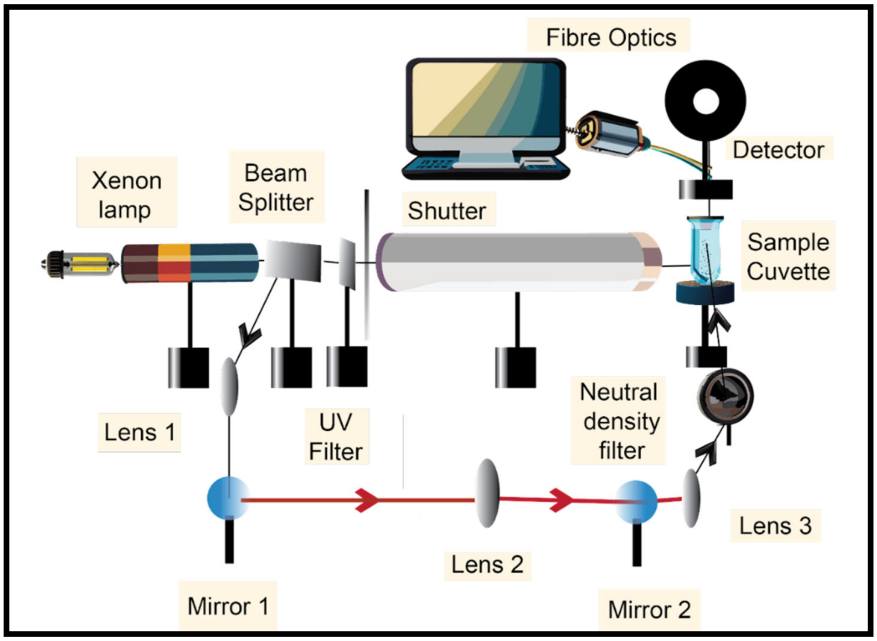


**Figure S4.** A representation of the setup inspired by pump-probe technique.

## *4. Fitting Procedure:*

All fits were calculated as a linear combination of selected reference spectra from those shown in Figure S5. The spectra for $H_2OCbl$ and HOCbl were obtained at pH 5 and 10, respectively, and were scaled in accordance with literature values.[5] The PhHxCbl spectrum was normalized to match the αβ band of PrCbl (ε ~9330 $M^{-1}cm^{-1}$).[4] The Co(I) component was obtained by complete conversion of a sample of PhHxCbl to cob(I)alamin via photolysis in the presence of excess $NaBH_4$. Similarly, PhHxCbl was completely photolyzed in the presence of excess 5,5-dimethyl-1-pyrroline *N*-oxide (DMPO), a radical trap, in order to obtain the cob(II)alamin component. These spectra were then normalized using the initial PhHxCbl concentration. Experimental spectra were fit across the wavelength range 340-650 nm.

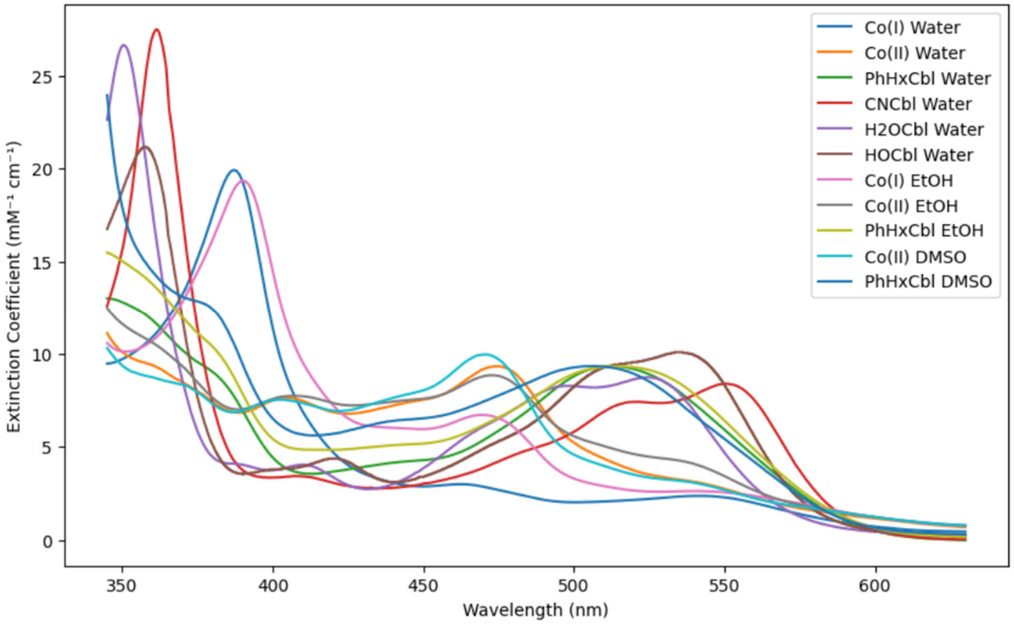


**Figure S5.** Full set of reference spectra used for fits.

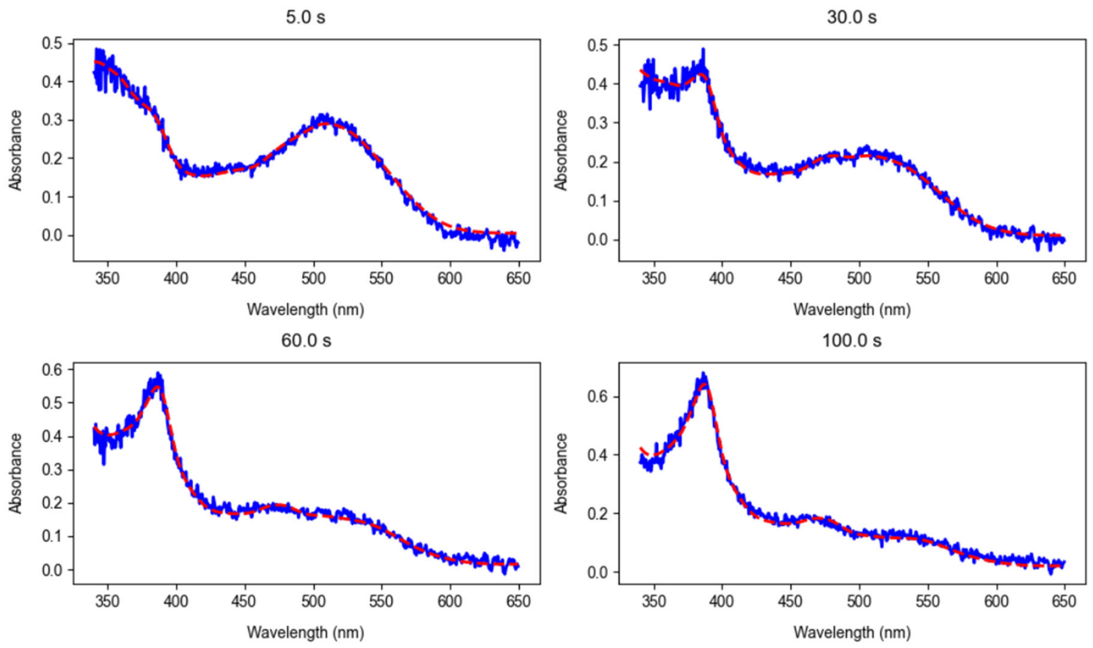


**Figure S6.** Experimental (blue) vs fitted (red) spectra corresponding to Figure 3d for time-resolved data of PhHxCbl in water at 5, 30, 60 and 100 seconds after photolysis.

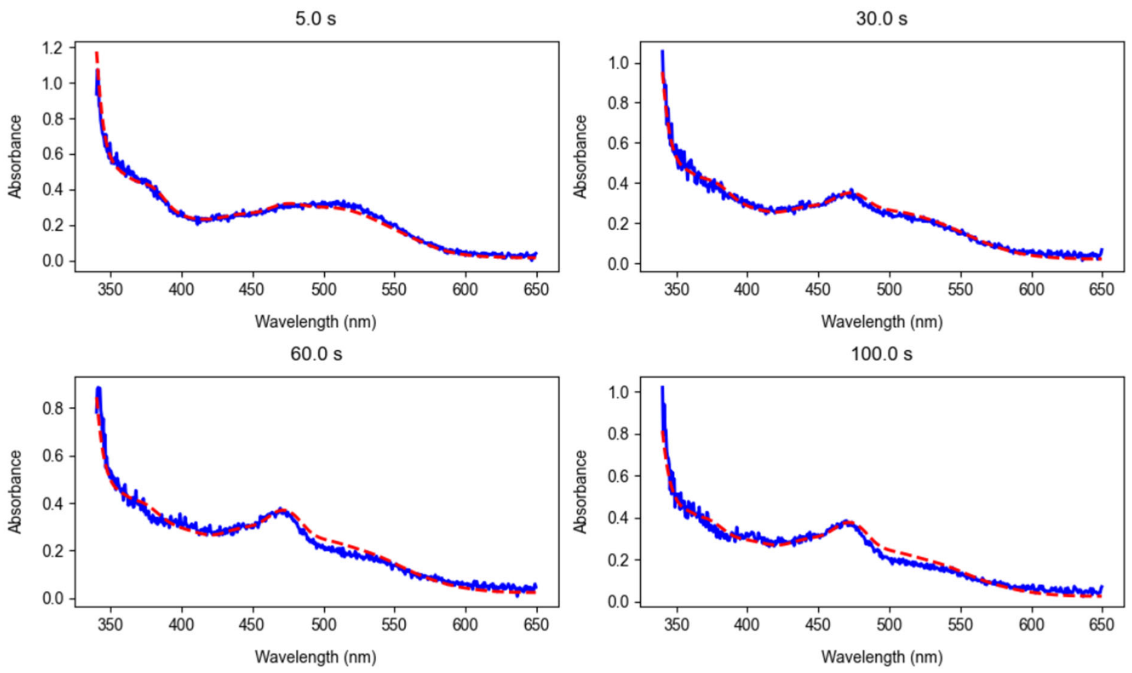


**Figure S7.** Experimental (blue) vs fitted (red) spectra corresponding to Figure 4d for time-resolved data of PhHxCbl in DMSO at 5, 30, 60 and 100 seconds after photolysis.

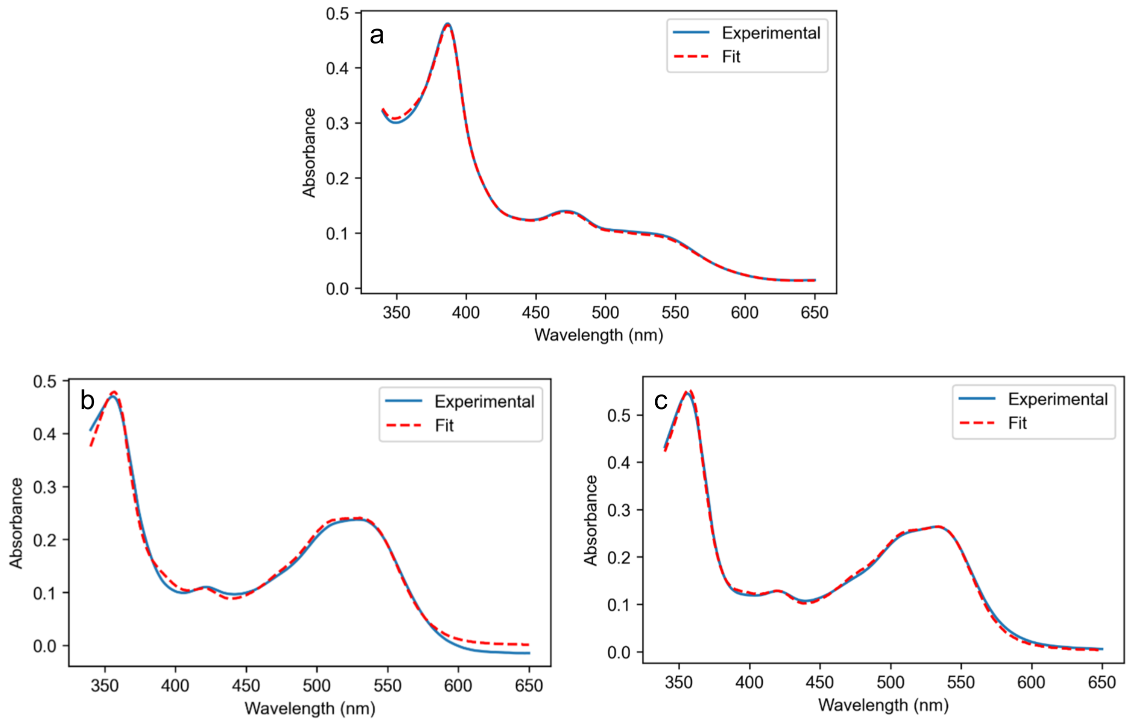


**Figure S8.** Experimental vs fitted spectra corresponding to (a) post-photolysis of PrCbl in water, (b) 12 minutes after adding excess 1-bromopropane to a photolyzed PrCbl sample that formed Co(I), kept in the dark, and (c) post-photolysis for the reaction of photolytically generated Co(I) in the presence of excess 1-bromopropane.

## *5. Steady State and Time-Resolved Spectral Measurements:*

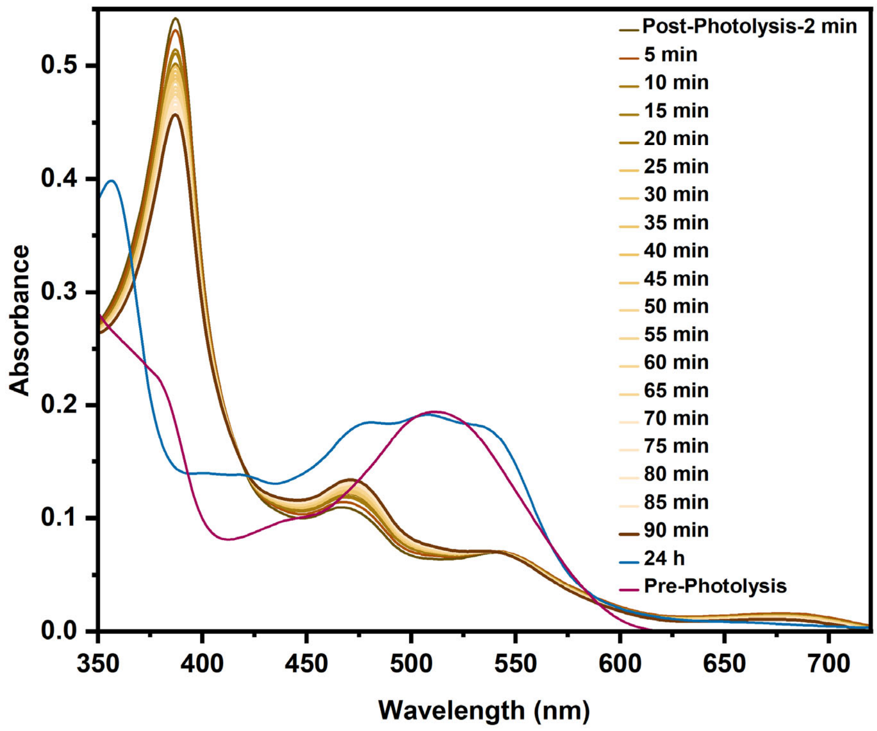


**Figure S9.** UV-Vis spectra showing the formation of Co(I) from the photolysis of PhHxCbl in water and the gradual decay over 90 minutes and after 24 hours in the dark.

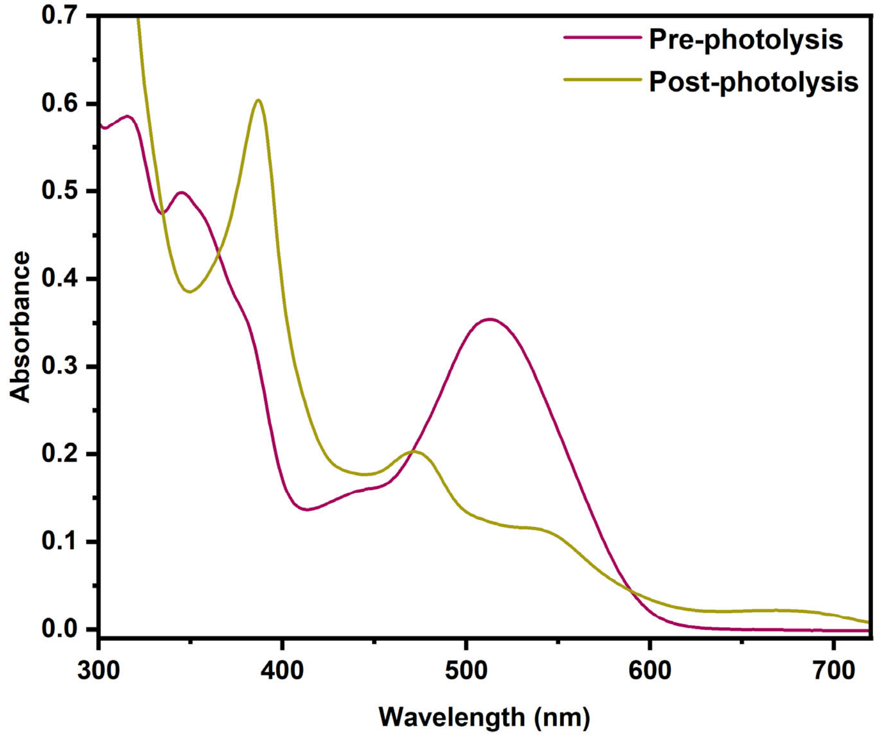


**Figure S10.** UV-Vis absorption spectra of PhHxCbl in water before and after photolysis for 2 minutes in the presence of sodium bicarbonate (30 equivalents).

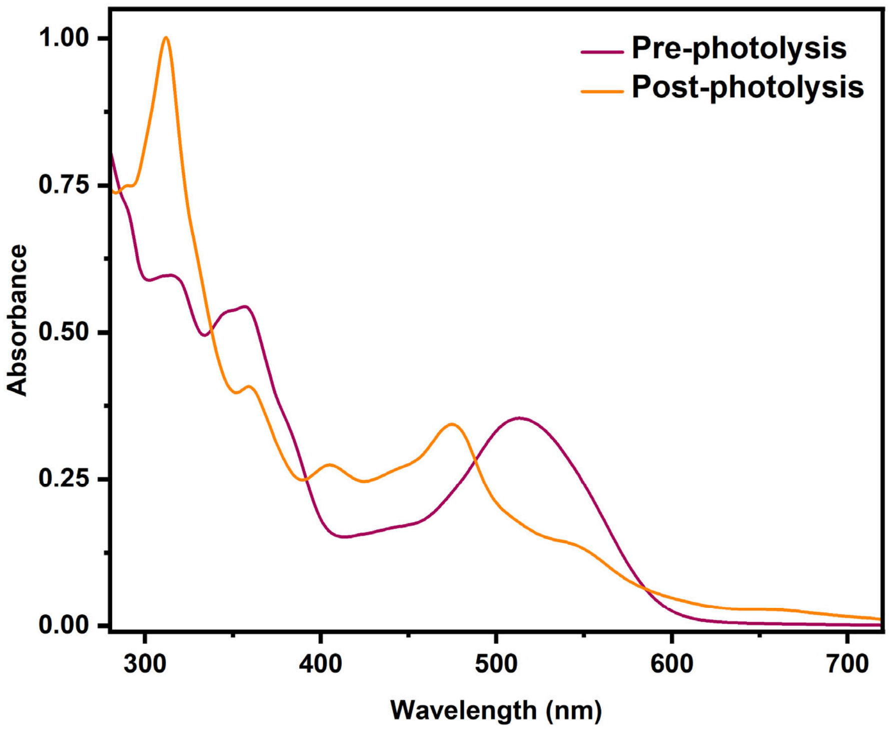


**Figure S11.** UV-Vis absorption spectra of PhHxCbl in water before and after photolysis for 2 minutes in the presence of DMPO.

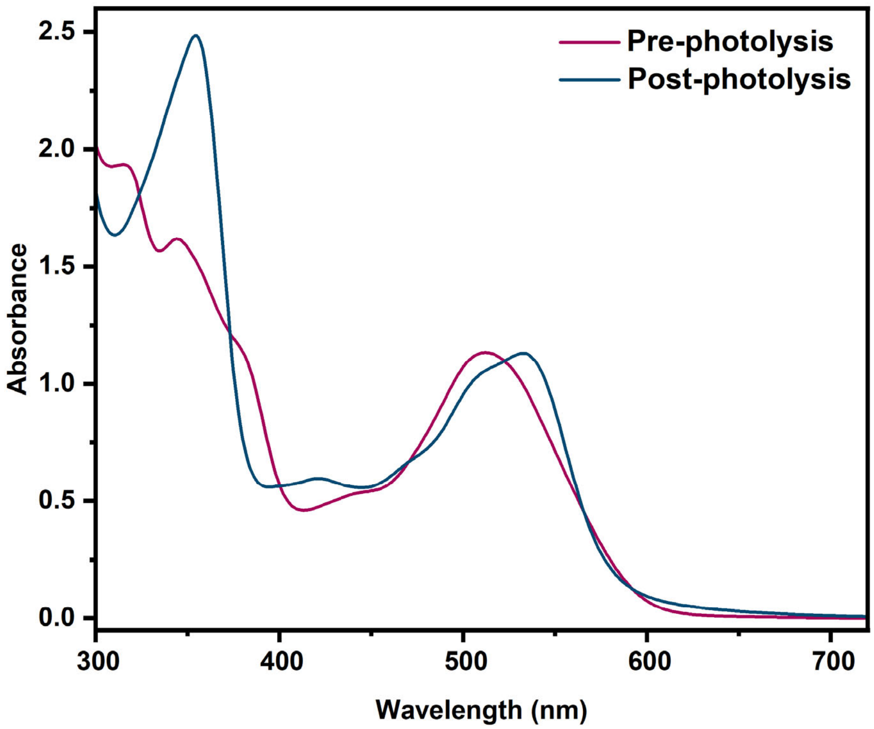


**Figure S12.** UV-Vis absorption spectra of PhHxCbl in water before and after photolysis for 5 minutes under aerobic conditions.

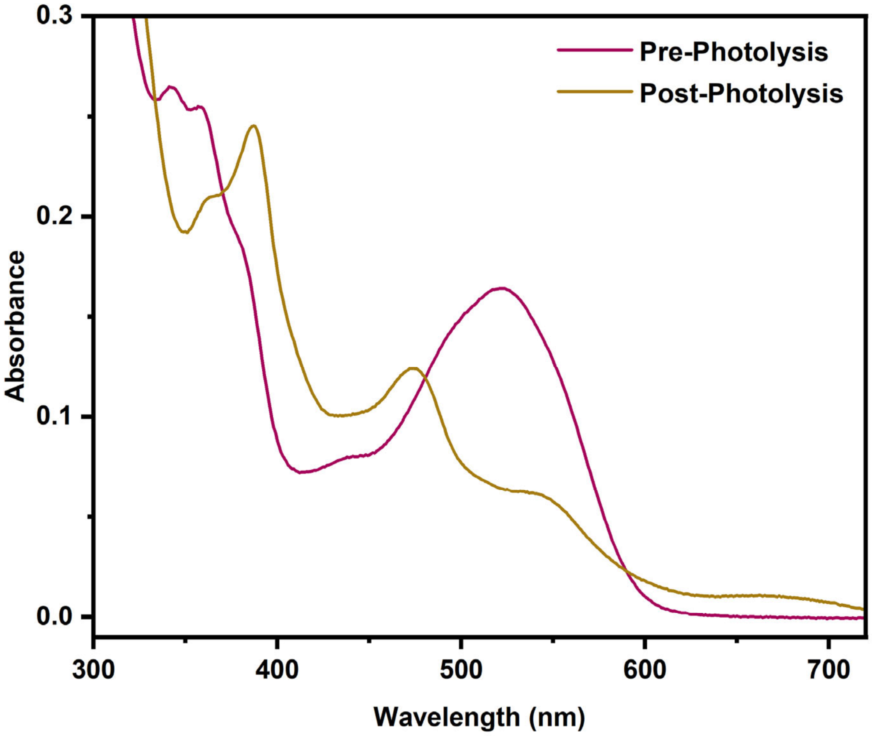


**Figure S13.** UV-Vis absorption spectra of NapEtCbl in water before and after photolysis for 2 minutes.

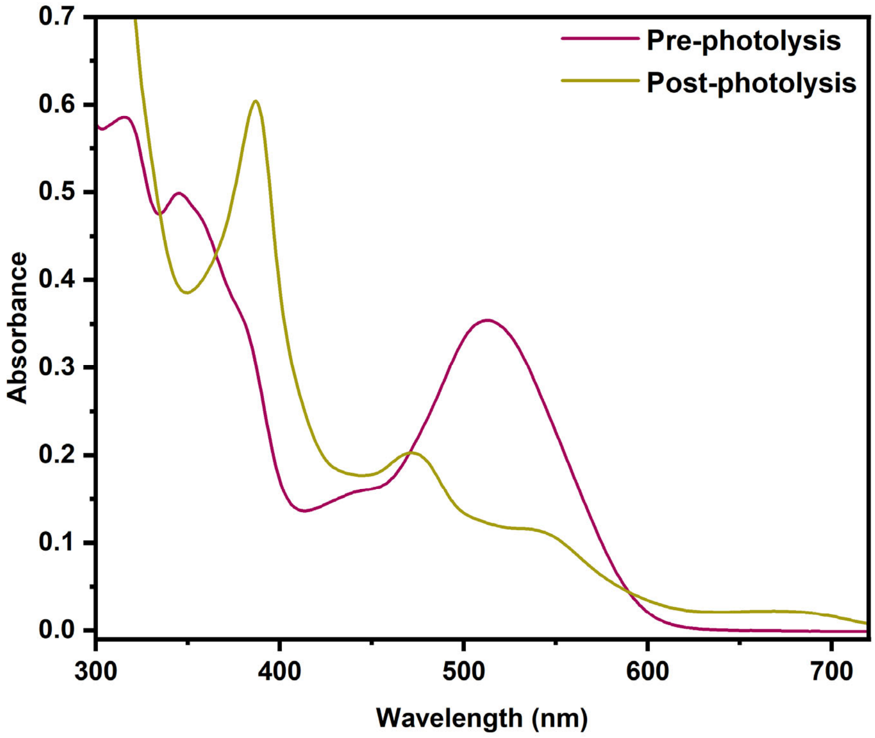


**Figure S14.** UV-Vis absorption spectra of NapEtCbl in water before and after photolysis for 2 minutes in the presence of sodium bicarbonate (30 equivalents).

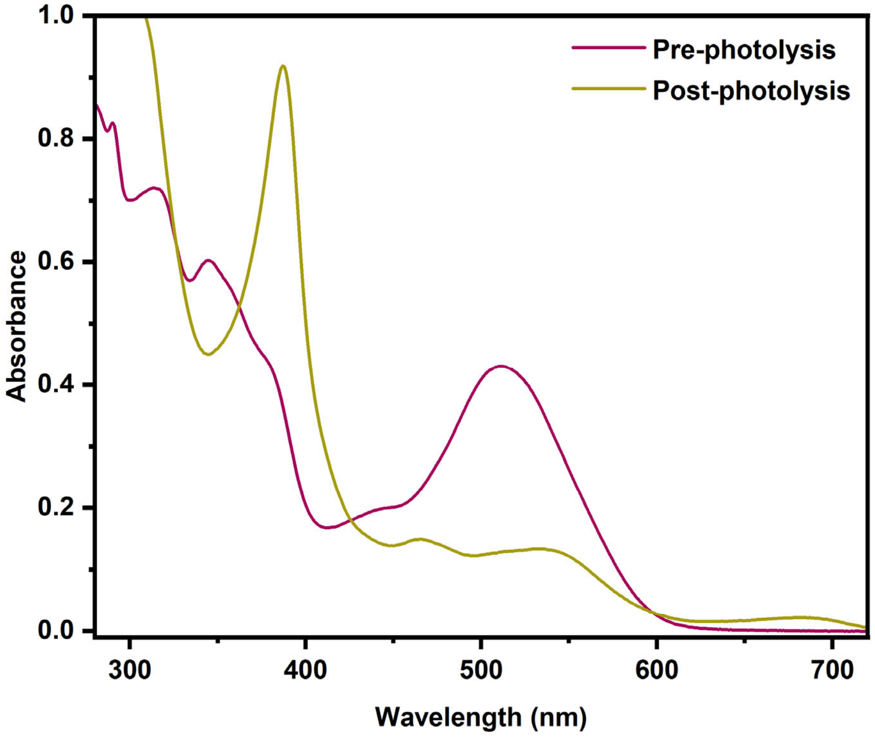


**Figure S15.** UV-Vis absorption spectra of PrCbl in water before and after photolysis for 2 minutes.

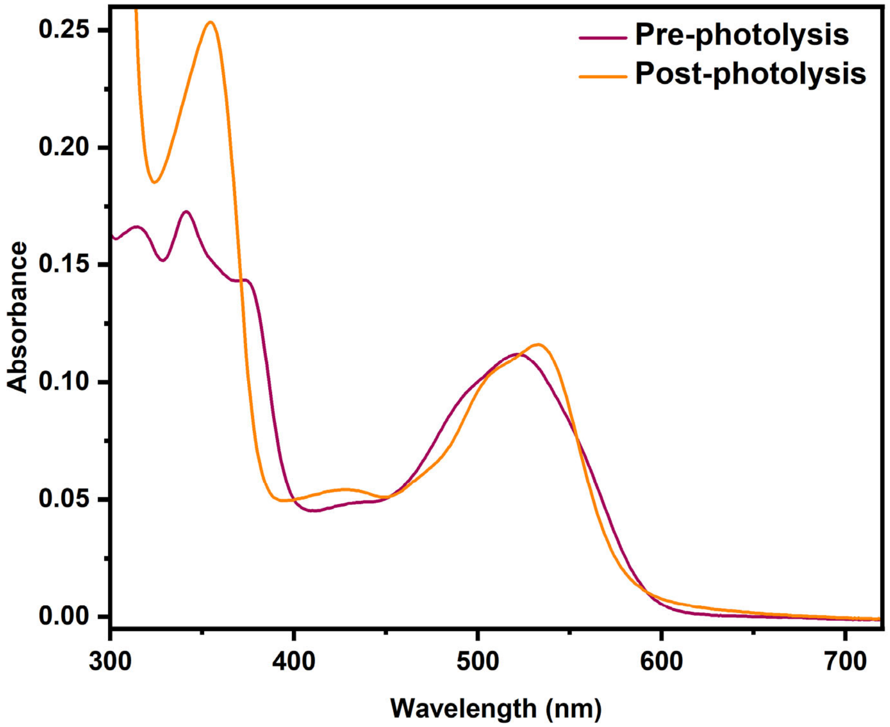


**Figure S16.** UV-Vis absorption spectra for MeCbl in water before and after photolysis for 2 minutes under aerobic conditions.

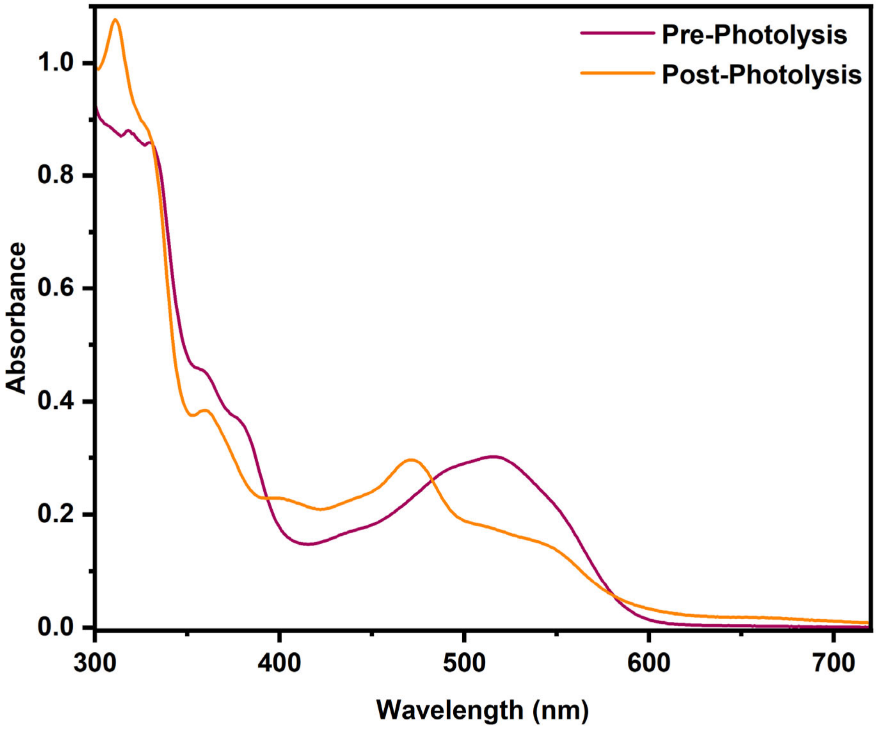


**Figure S17.** UV-Vis absorption spectra of NapEtCbl in DMSO before and after photolysis for 2 minutes.

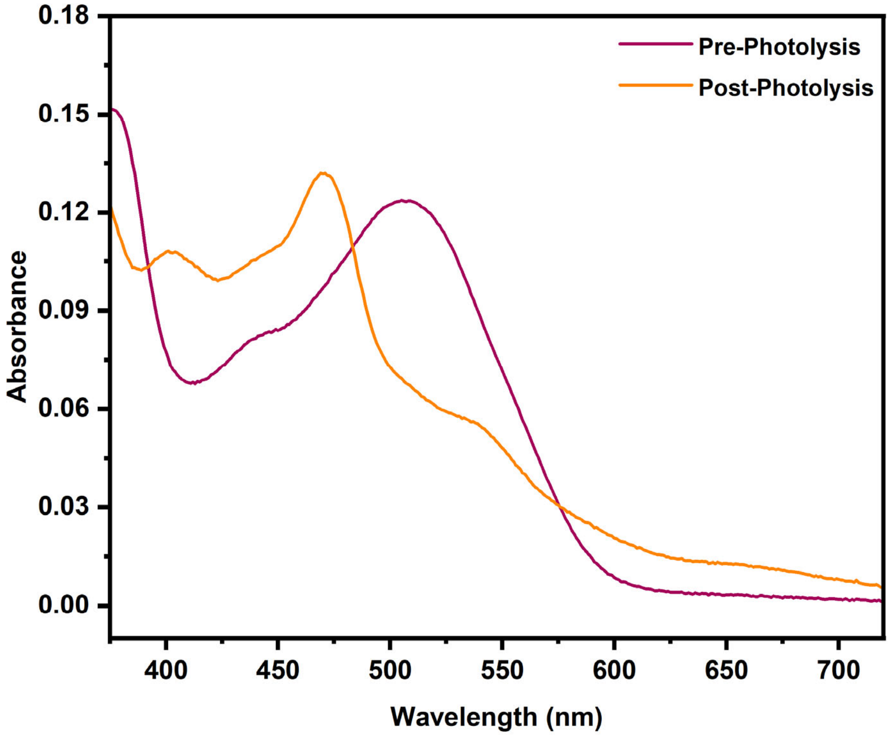


**Figure S18.** UV-Vis absorption spectra of PhHxCbl in DMSO before and after photolysis for 2 minutes in the presence of sodium bicarbonate (30 equivalents).

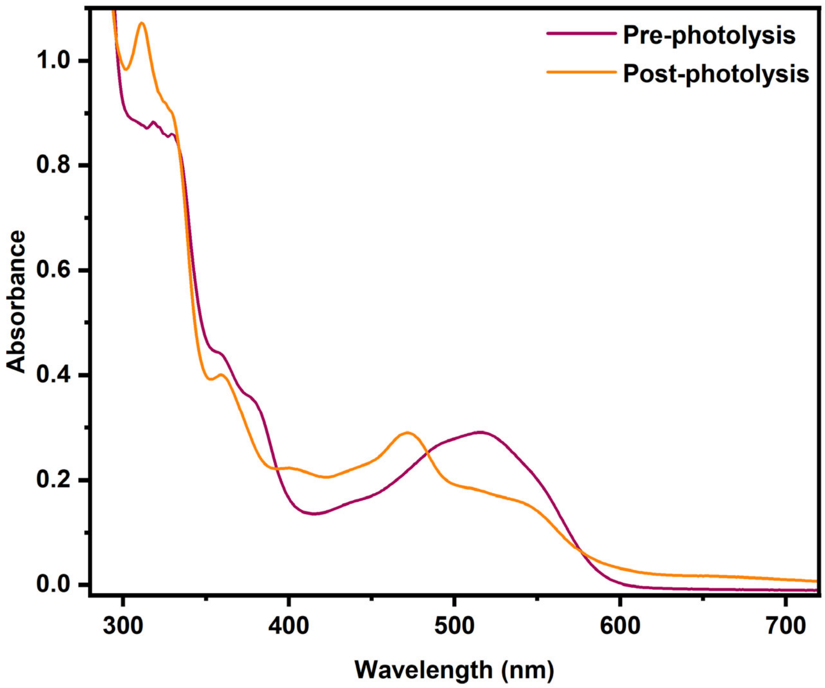


**Figure S19.** UV-Vis absorption spectra of NapEtCbl in DMSO before and after photolysis for 2 minutes in the presence of sodium bicarbonate (30 equivalents).

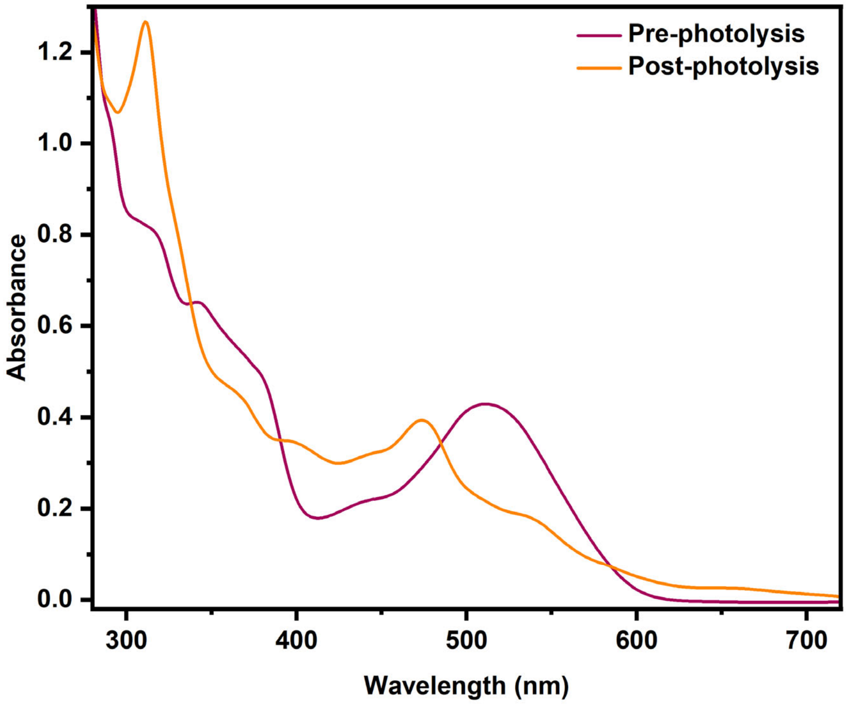


**Figure S20.** UV-Vis absorption spectra of PhHxCbl in methanol before and after photolysis for 2 minutes.

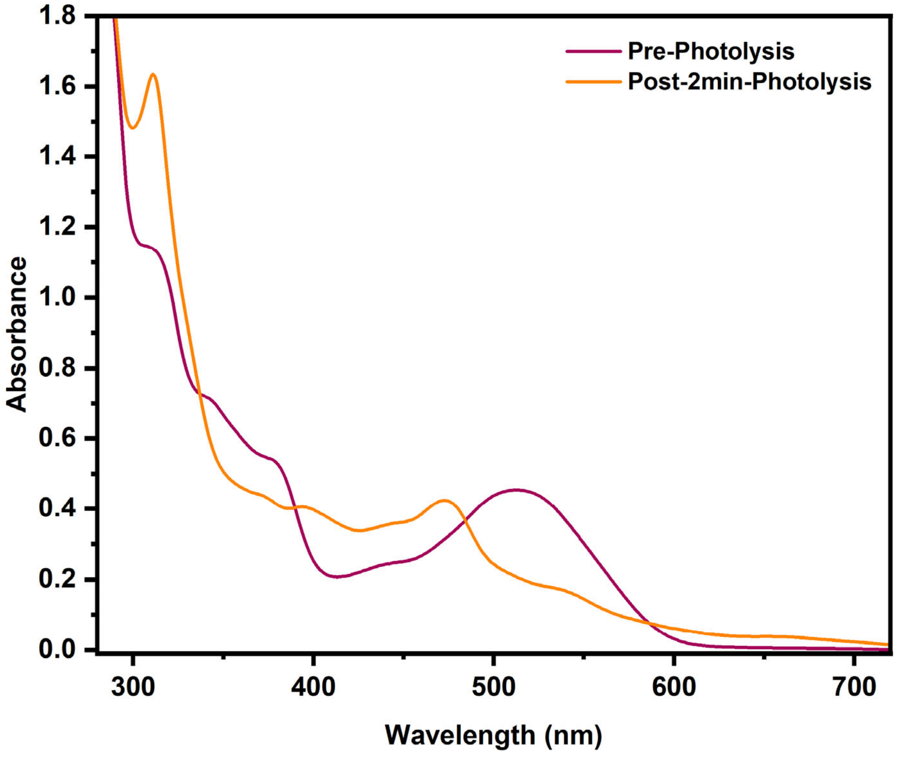


**Figure S21.** UV-Vis absorption spectra of PhHxCbl in ethanol before and after photolysis for 2 minutes.

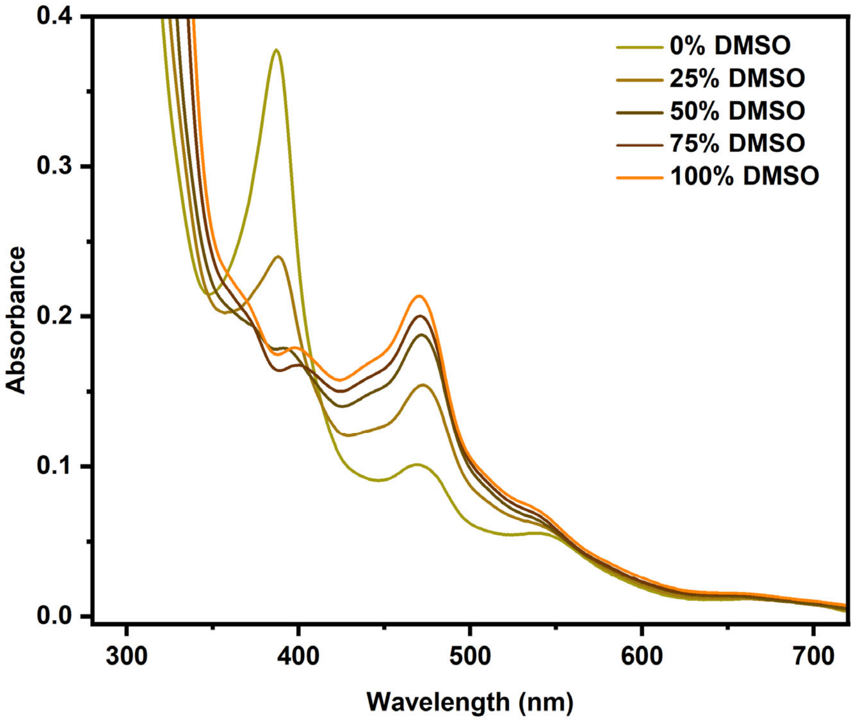


**Figure S22.** UV-Vis absorption spectra of PhHxCbl photolyzed for 2 minutes in varying ratios of DMSO in water (% v/v).

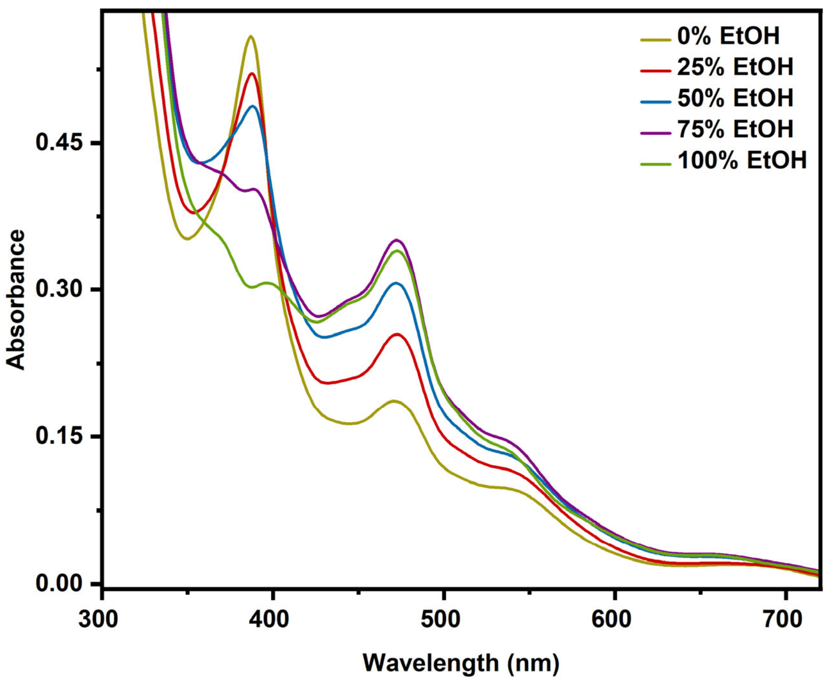


**Figure S23.** UV-Vis absorption spectra of PhHxCbl photolyzed for 2 minutes in varying ratios of ethanol in water (% v/v).

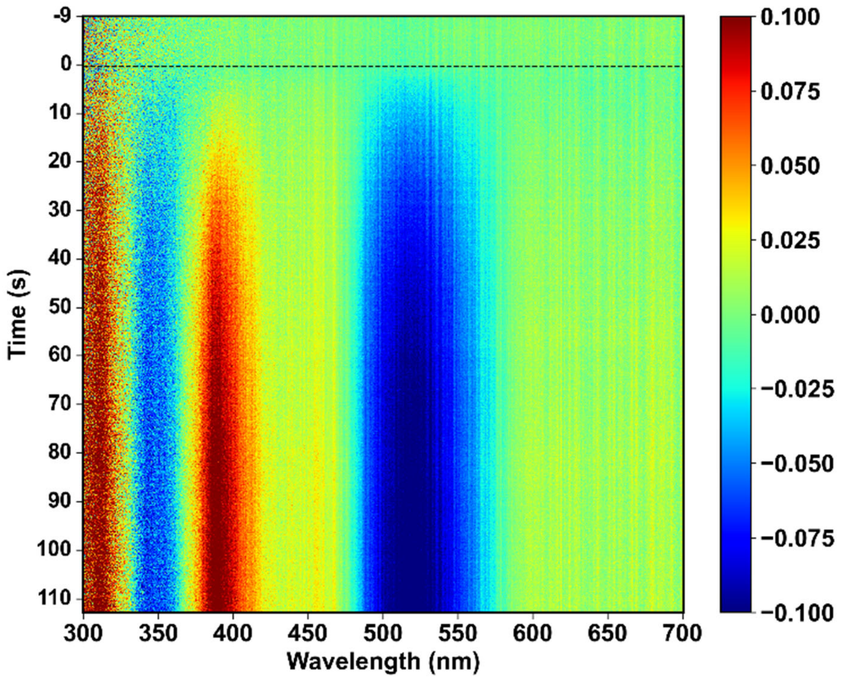


**Figure S24.** Time-resolved UV-Vis difference absorption spectra for NapEtCbl in water. The growth of the yellow-red region shows the generation of Co(I) while the bleach for Co(III) is represented by the blue region.

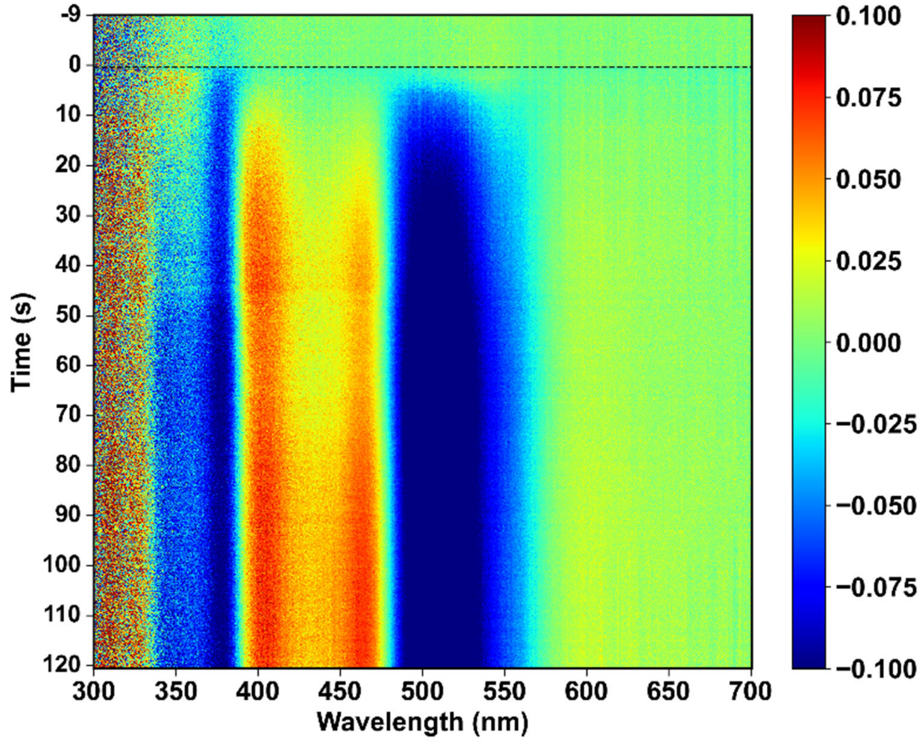


**Figure S25.** Time-resolved UV-Vis difference absorption spectra for PhHxCbl in DMSO in the presence of sodium bicarbonate. The growth of the yellow-red region shows the generation of Co(II) while the bleach for Co(III) is represented by the blue region.

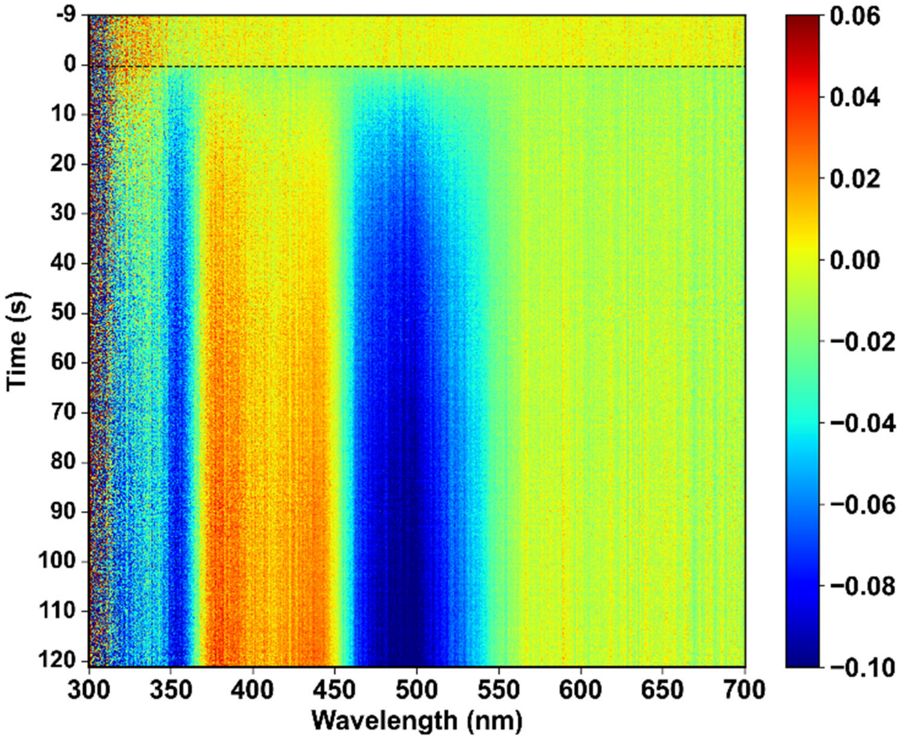


**Figure S26.** Time-resolved UV-Vis difference absorption spectra for NapEtCbl in DMSO.

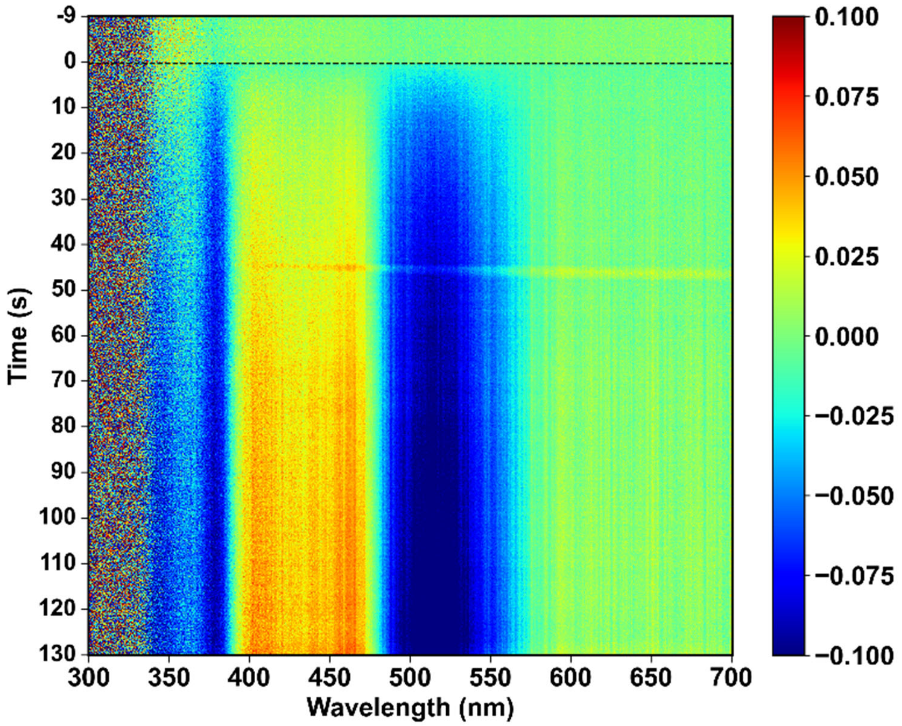


**Figure S27.** Time-resolved UV-Vis difference absorption spectra for NapEtCbl in DMSO in the presence of sodium bicarbonate.

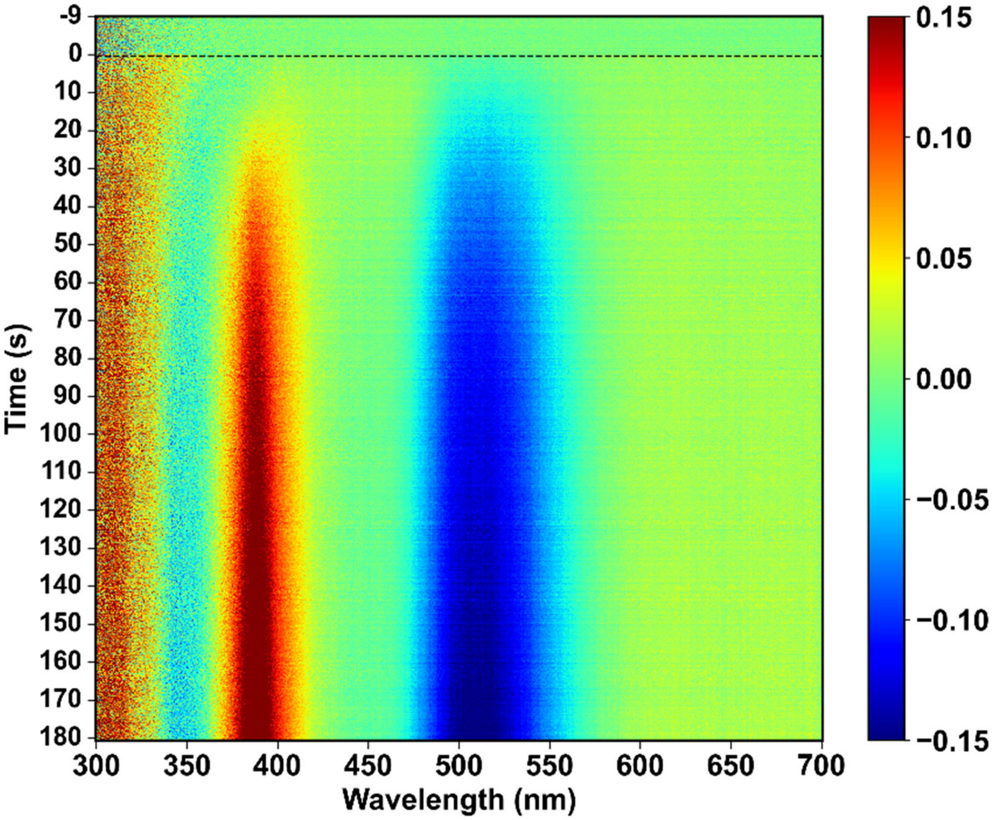


**Figure S28.** Time-resolved UV-Vis difference absorption spectra for PrCbl in water.

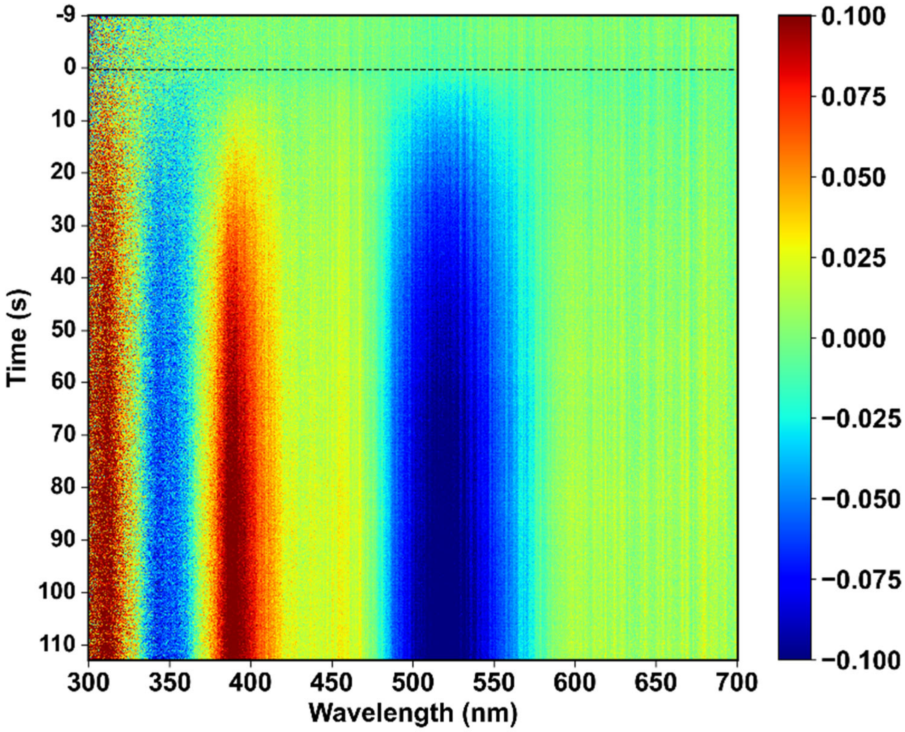


**Figure S29.** Time-resolved UV-Vis difference absorption spectra for PhHxCbl in 2:1 water-ethanol (v/v).

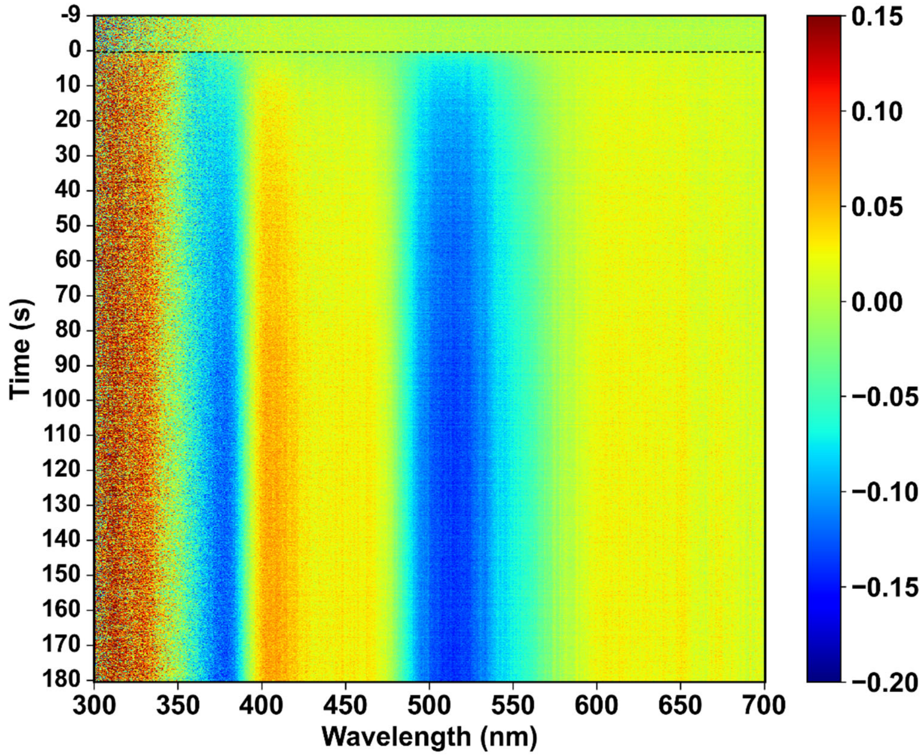


**Figure S30.** Time-resolved UV-Vis difference absorption spectra for PhHxCbl in methanol.

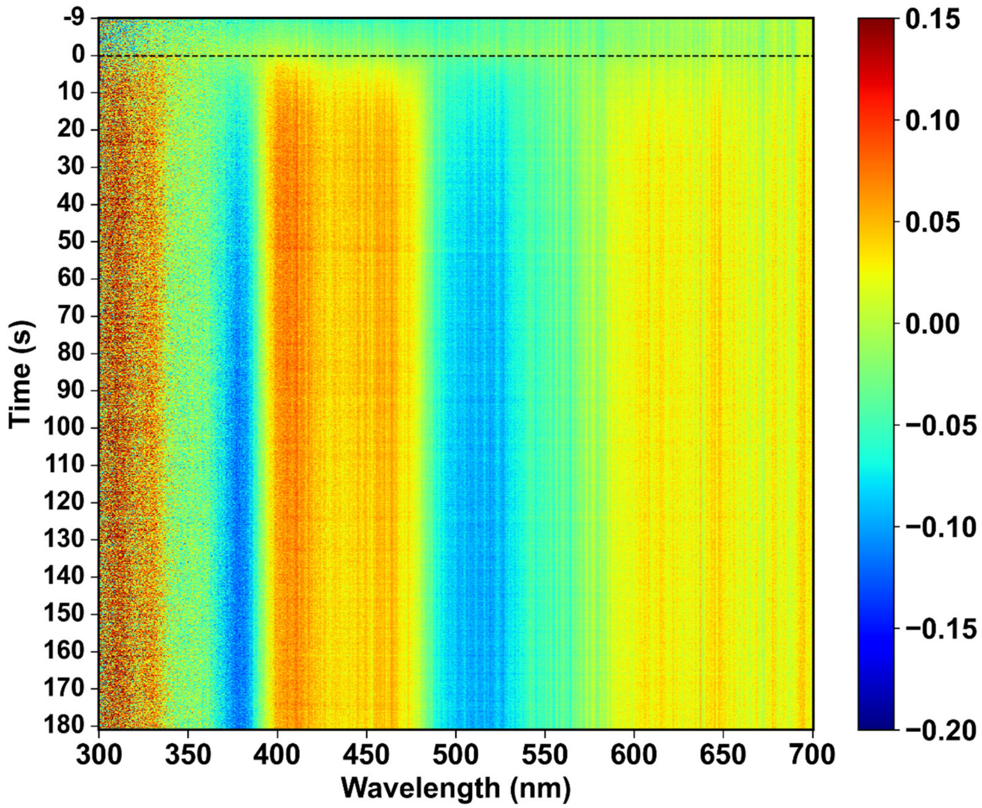


**Figure S31.** Time-resolved UV-Vis difference absorption spectra for PhHxCbl in ethanol.

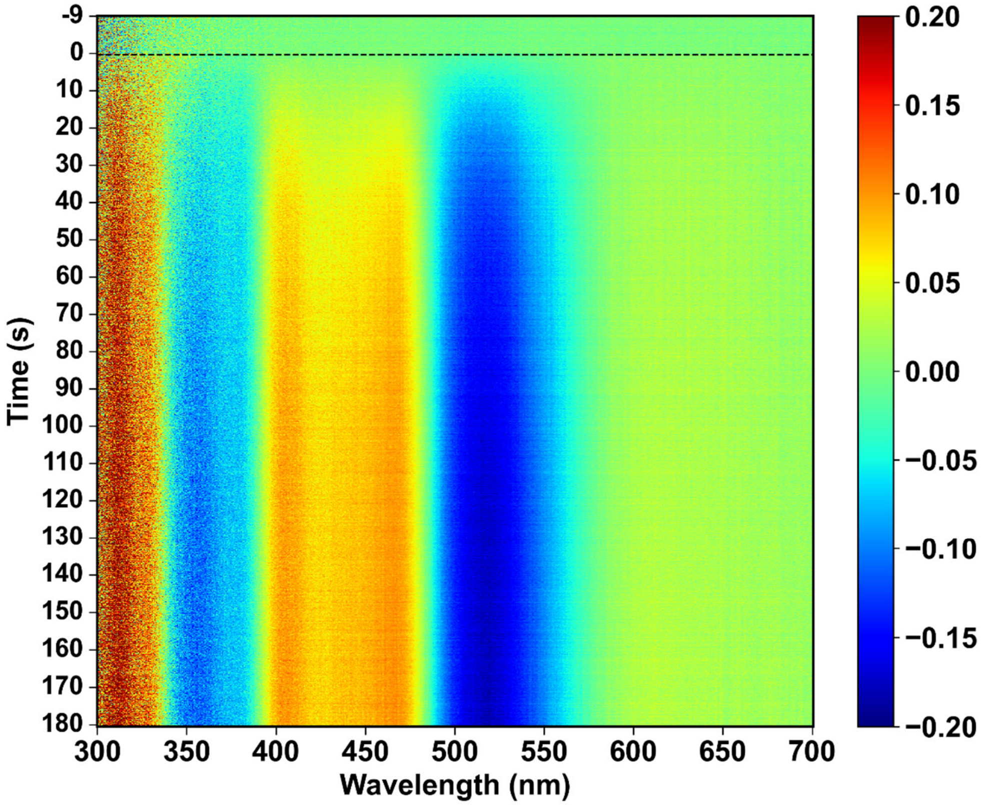


**Figure S32.** Time-resolved UV-Vis difference absorption spectra for PhHxCbl in water in the presence of DMPO.

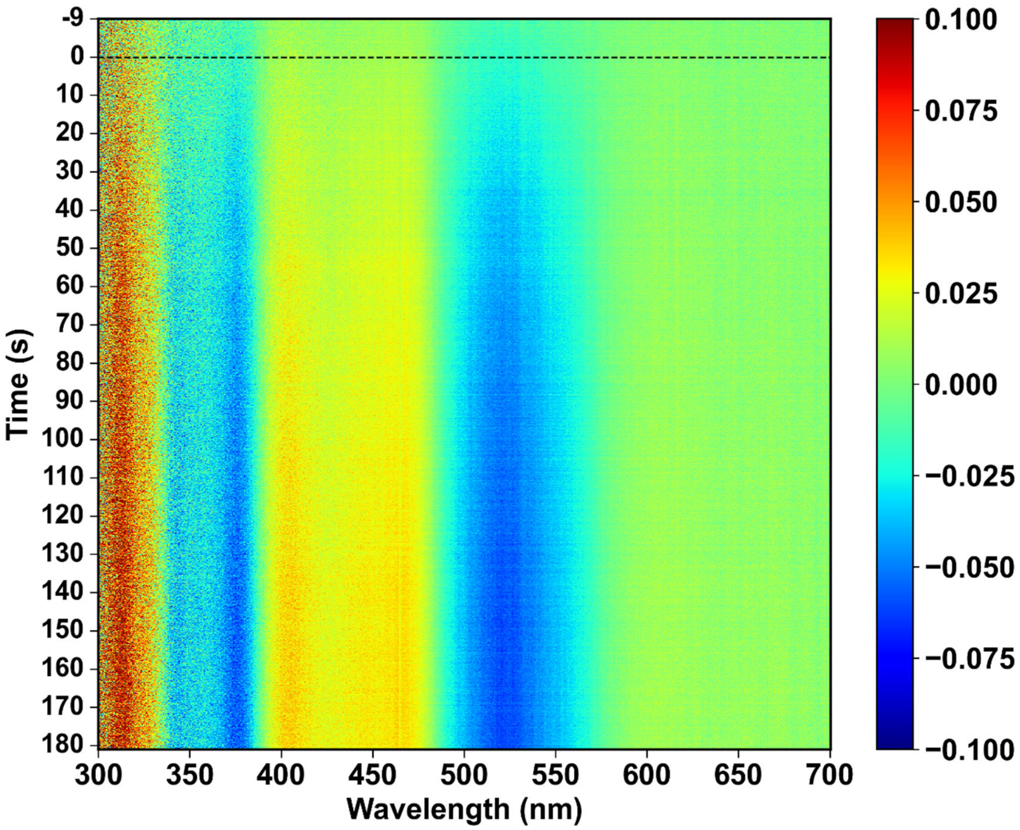


**Figure S33.** Time-resolved UV-Vis difference absorption spectra for MeCbl in water in the presence of TEMPO.

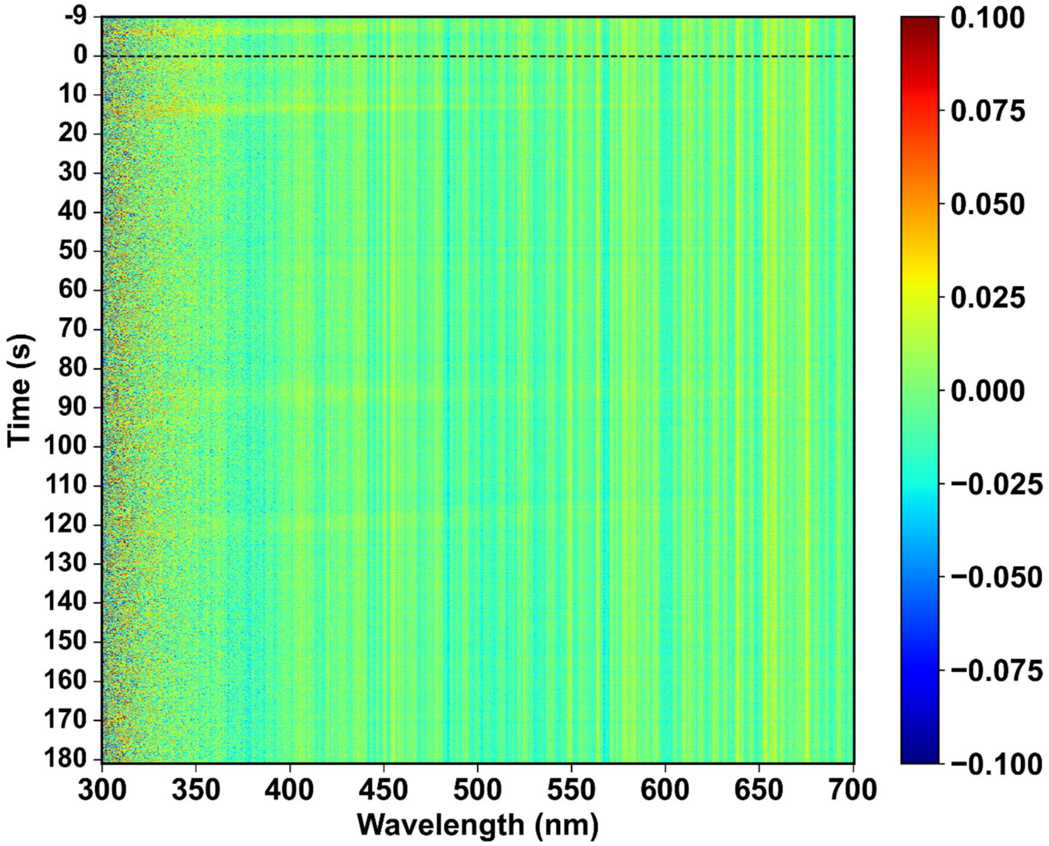


**Figure S34.** Time-resolved UV-Vis difference absorption spectra for photolysis of MeCbl in water under anaerobic conditions showing almost no change over 3 minutes.

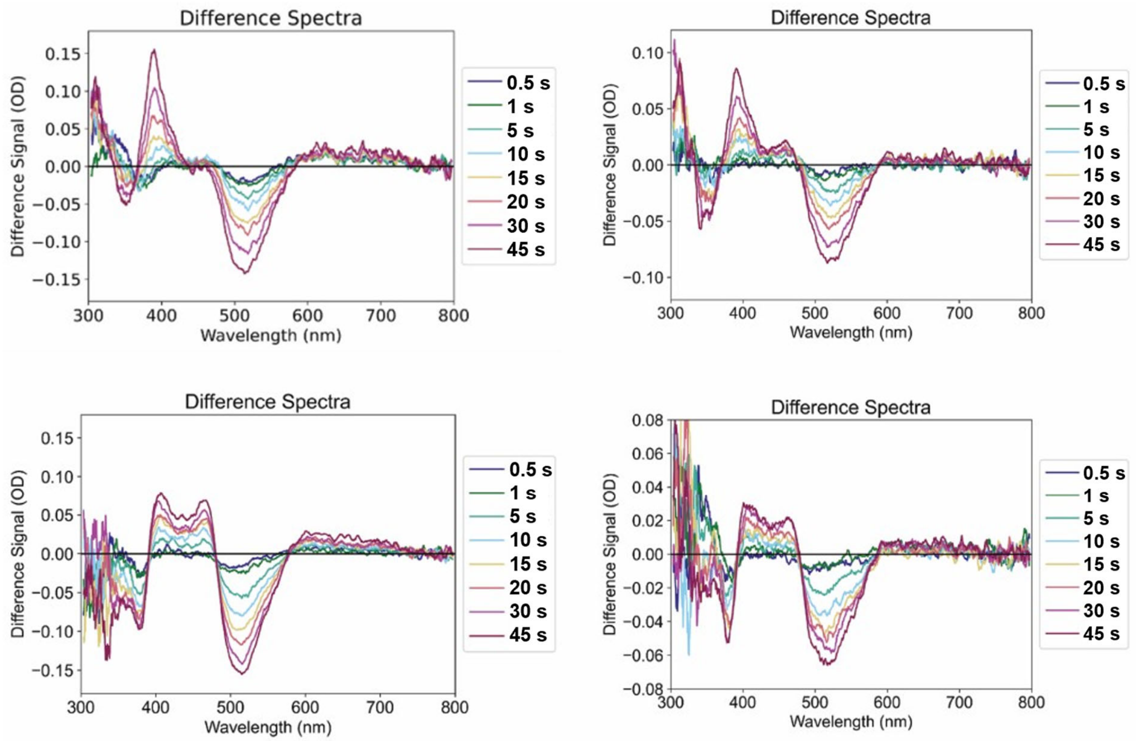


**Figure S35.** Difference absorbance spectra for photolysis of PhHxCbl in water (top-left) and DMSO (bottom-left), and NapEtCbl in water (top-right) and DMSO (bottom-right) over 45 seconds of photolysis.

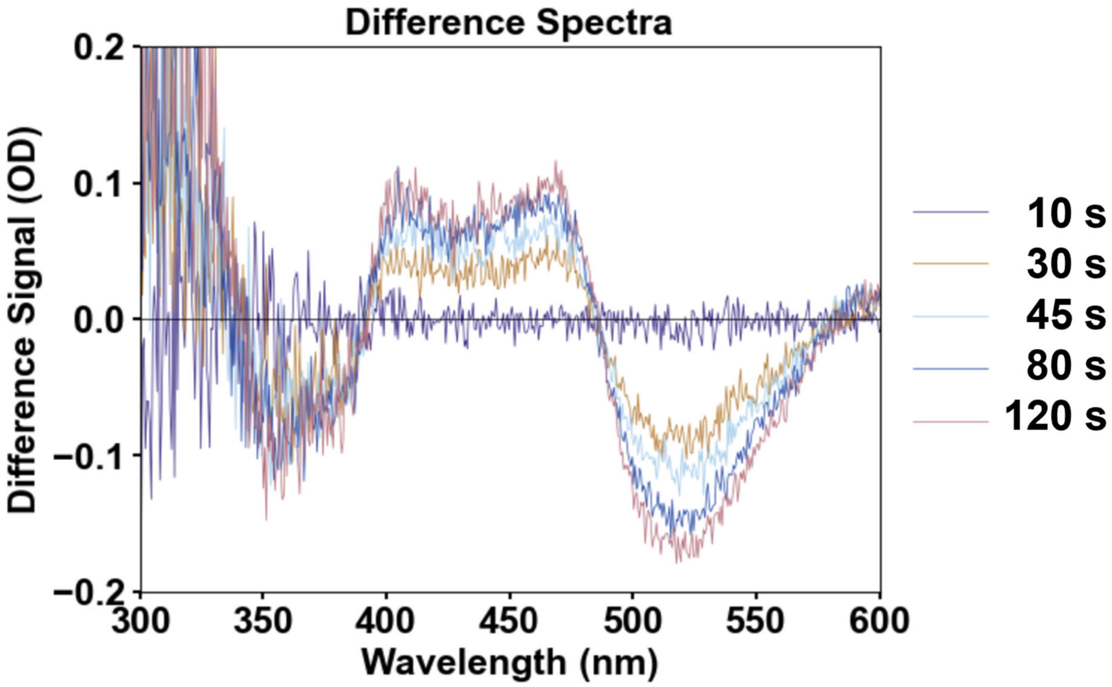


**Figure S36.** Difference absorbance spectra for photolysis of PhHxCbl in water in the presence of DMPO over 120 seconds.

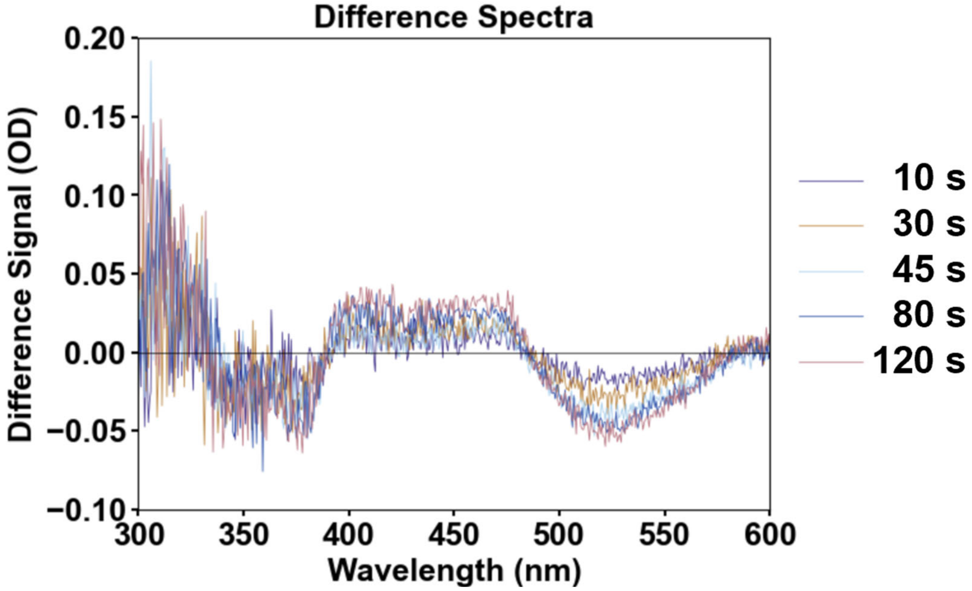


**Figure S37.** Difference absorbance spectra for photolysis of MeCbl in water in the presence of TEMPO over 120 seconds.

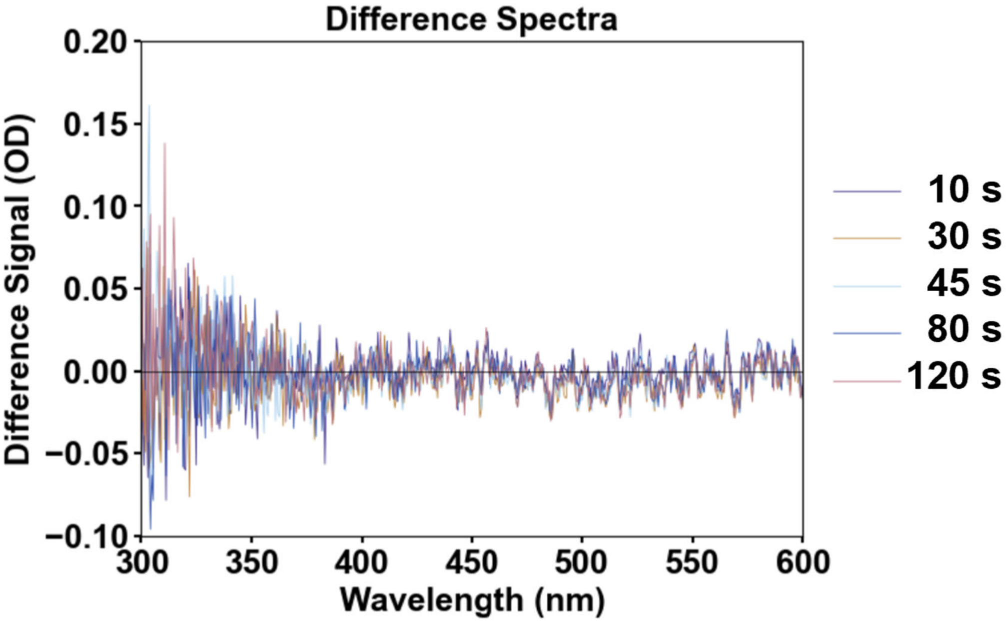


**Figure S38.** Difference absorbance spectra for photolysis of MeCbl in water over 120 seconds.

## *6. Mechanistic studies into the formation of alkylcobalamins:*

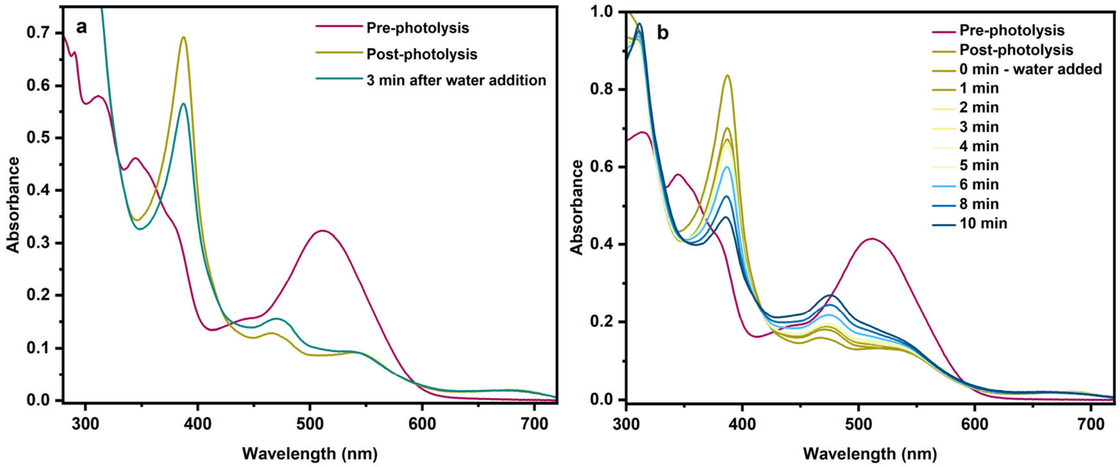


**Figure S39.** (a) Control experiment showing formation of the Co(I) species for a PrCbl sample in water followed by the addition of ~100 µl of degassed water showing only minimal oxidation of Co(I) to Co(II) over 3 minutes after addition while kept in the dark (Table S1). (b) Monitoring the change in the UV-Vis spectra after addition of the water followed by formation of some Co(II), most likely due to some oxygen contamination during addition. (Table S2). Some runs were collected after shaking in between to ensure proper mixing.

**Table S1.** Fits for spectra of a PrCbl sample in water, with water added after photolysis as a control for alkyl halide addition (data corresponding to Figure S39a). The standard deviation in ΔPrCbl/|ΔCo(I)| before and after water addition is zero.

| Entry | Sample | % Co(I) | % Co(II) | % PrCbl | % $H_2$OCbl | % HOCbl |
|---|---|---|---|---|---|---|
| 1 | pre-photolysis | 4 | 5 | 91 | 0 | 0 |
| 2 | post-photolysis before water addition | 77 | 13 | 9 | 0 | 0 |
| 3 | 3 minutes after water addition | 62 | 29 | 9 | 0 | 0 |

**Table S2.** Fits for spectra of a PrCbl sample in water, with water added after photolysis as a control for alkyl halide addition (data corresponding to Figure S39b).

| Entry | Sample | % Co(I) | % Co(II) | % PrCbl | % $H_2O$Cbl | % HOCbl |
|---|---|---|---|---|---|---|
| 1 | pre-photolysis | 3 | 5 | 92 | 0 | 0 |
| 2 | post-photolysis before water addition | 86 | 5 | 9 | 0 | 0 |
| 3 | 0 minutes after water addition | 73 | 18 | 9 | 0 | 0 |
| 4 | 1 minute after water addition | 68 | 21 | 11 | 0 | 0 |
| 5 | 2 minutes after water addition | 67 | 22 | 12 | 0 | 0 |
| 6 | 3 minutes after water addition | 66 | 22 | 12 | 0 | 0 |
| 7 | 4 minutes after water addition | 65 | 22 | 13 | 0 | 0 |
| 8 | 5 minutes after water addition | 64 | 23 | 13 | 0 | 0 |
| 9 | 6 minutes after water addition | 55 | 30 | 14 | 0 | 0 |
| 10 | 8 minutes after water addition | 43 | 41 | 16 | 0 | 0 |
| 11 | 10 minutes after water addition | 33 | 50 | 17 | 0 | 0 |

NB. As a control, to ensure that the Co(II) formation was not the result of $O_2$ contamination, we added an equivalent volume of degassed water to the cuvette after alkylCbl photolysis. Here, we did not observe any major change, only the oxidation of a small amount (~15%) of Co(I) to Co(II), significantly less than the observed 41% change in Co(II) upon RBr addition (Tables S1 and S2).

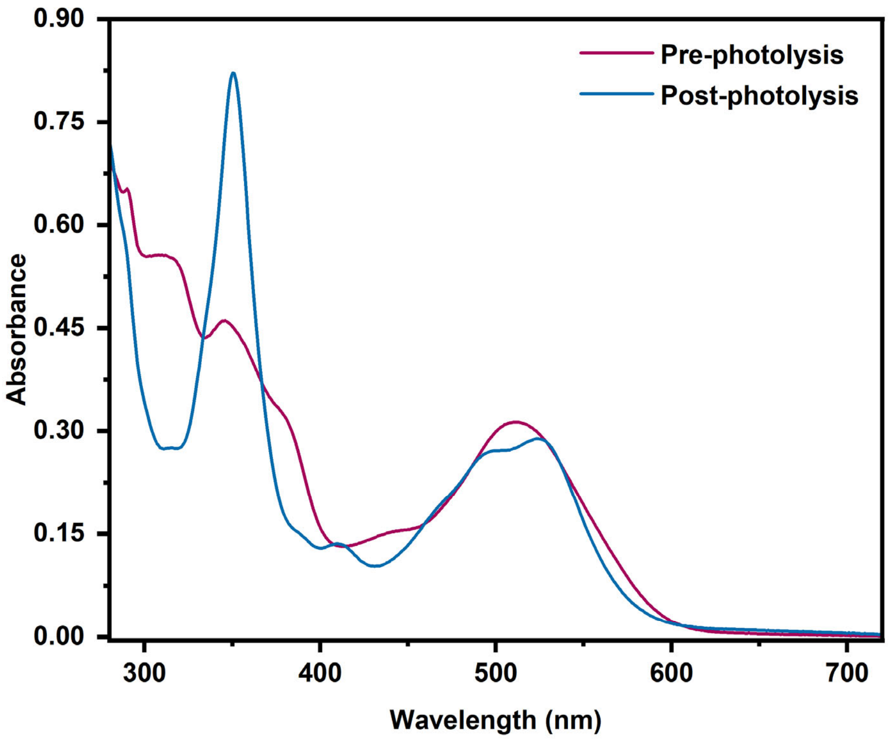


**Figure S40.** UV-Vis absorbance spectra showing the formation of the $H_2OCbl$/HOCbl species from photolysis of PrCbl in water in the presence of excess 1-bromopropane (a PrCbl stock solution in water saturated with the alkyl halide) (Table S3).

**Table S3.** Fits for spectra of the PrCbl sample photolyzed in water in the presence of ~100 µl of 1-bromopropane (data corresponding to the blue spectrum in Figure S40). The standard deviation in ΔPrCbl before and after photolysis in these experiments is 4.5%.

| Entry | Sample | % Co(I) | % Co(II) | % PrCbl | % $H_2OCbl$ | % HOCbl |
|---|---|---|---|---|---|---|
| 1 | pre-photolysis | 4 | 9 | 87 | 0 | 0 |
| 2 | post-photolysis – 0 minutes | 1 | 2 | 9 | 81 | 6 |

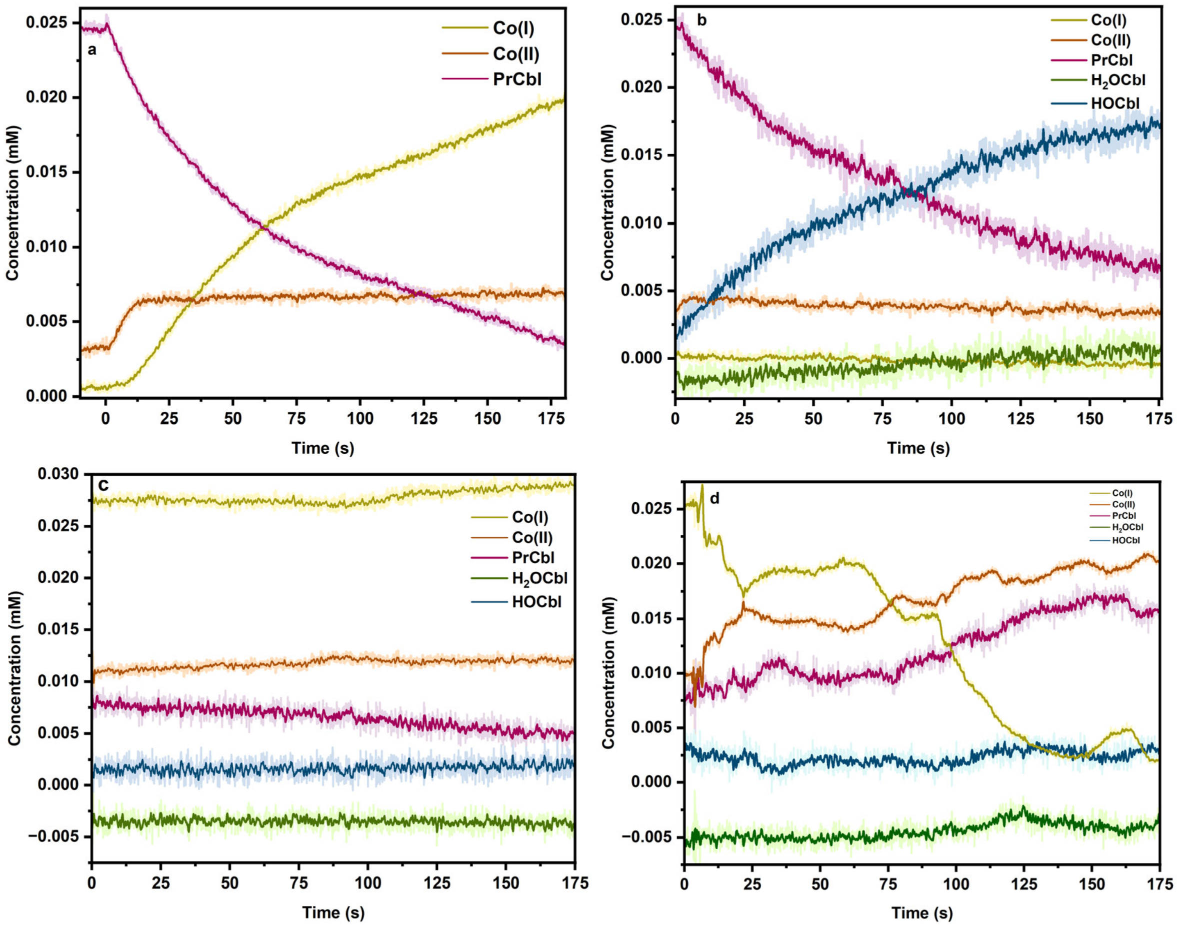


**Figure S41.** Fits for the time-resolved experiments to see potential turnover with alkyl halide addition: (a) Photolysis of PrCbl in water showing formation of Co(I). (b) Photolysis of PrCbl in water with ~20 mM 1-bromopropane (aqueous solution saturated with the alkyl halide) present. (c) Control experiment showing almost no change in Co(I) and Co(II) concentrations and no formation of $H_2OCbl$/HOCbl observed after addition of 100 µL water to a Co(I) sample at t = 0. (d) Increase in Co(II) and PrCbl concentration alongside decrease in Co(I) observed after addition of 100 µL of 1-bromopropane to a Co(I) sample at t = 0. Fits are obtained as a linear combination of various references across the wavelength range 340-650 nm.

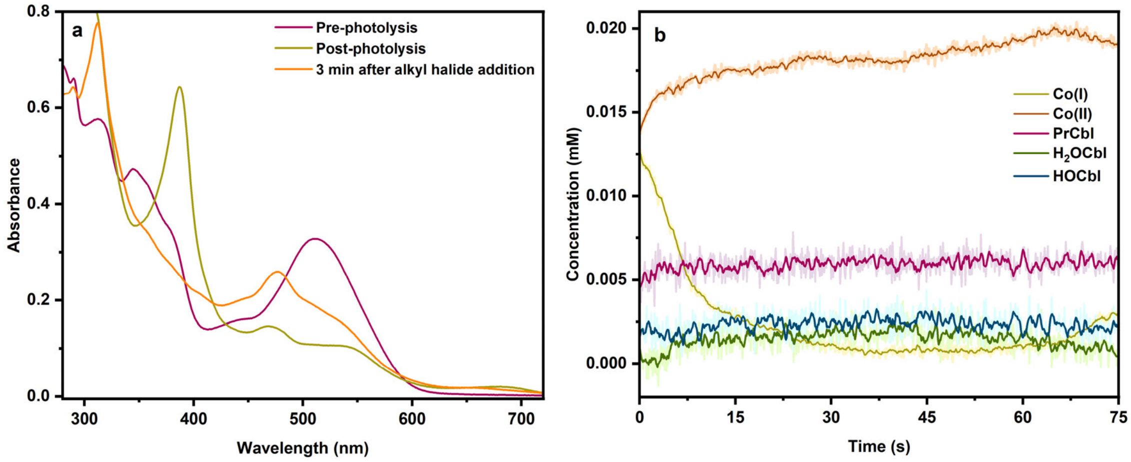


**Figure S42.** (a) Photolysis of PrCbl (0.03 mM) showing formation of Co(I) followed by addition of 50 equivalents of 1-bromopropane (1.5 mM), reacted for 3 minutes in the dark. (b) Fits of the first 75 seconds of reaction time. Fits are obtained as a linear combination of various references across the wavelength range 340-650 nm.

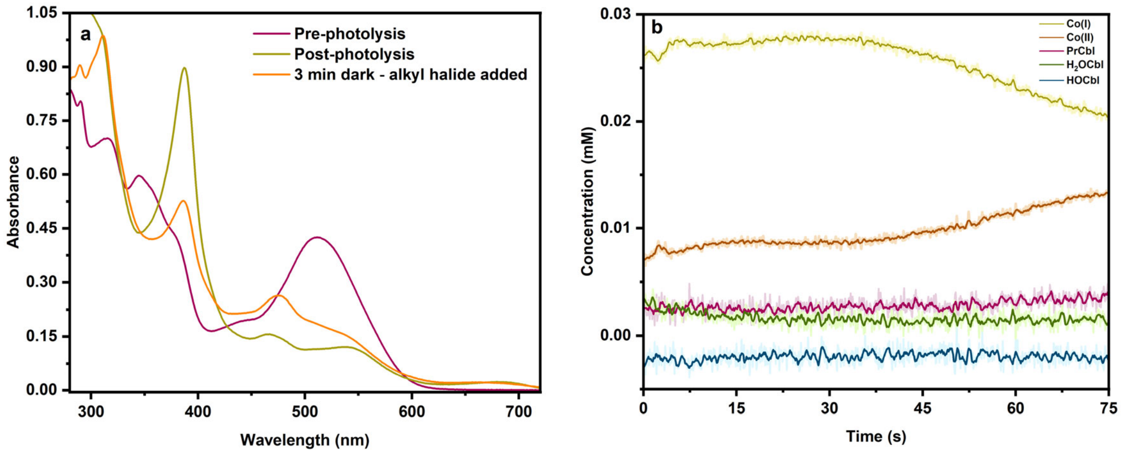


**Figure S43.** (a) Photolysis of PrCbl (0.03 mM) showing formation of Co(I) followed by addition of 25 equivalents of 1-bromopropane (0.75 mM), reacted for 3 minutes in the dark. (b) Fits of the first 75 seconds of reaction time. Fits are obtained as a linear combination of various references across the wavelength range 340-650 nm.

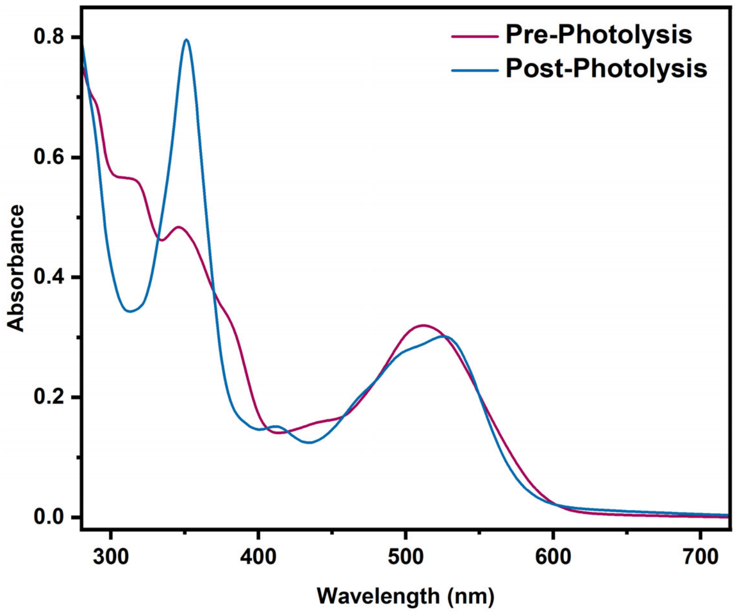


**Figure S44.** Formation of the $H_2OCbl$/HOCbl species from photolysis of PhHxCbl in water in the presence of excess 1-bromopropane (a PhHxCbl stock solution in water saturated with the alkyl halide).

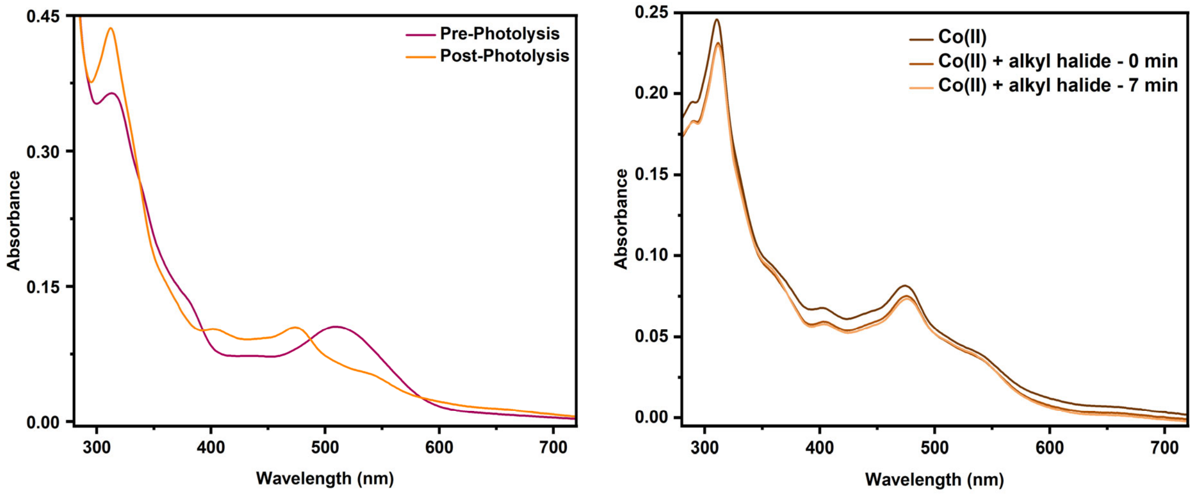


**Figure S45.** Photolysis of PrCbl in an aqueous solution saturated with 1-bromopropane in the presence of DMPO (left). Photolysis of PrCbl in the presence of DMPO followed by addition of excess 1-bromopropane (right). The formed Co(II) is non-reactive towards the alkyl halide.

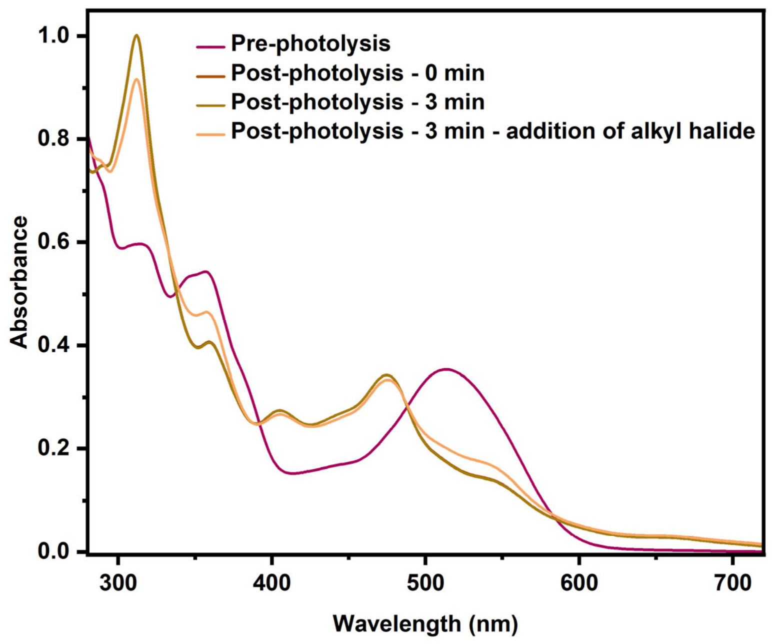


**Figure S46.** Photolysis of PhHxCbl in the presence of DMPO followed by addition of excess 1-bromopropane. The formed Co(II) is non-reactive towards the alkyl halide. NB. There was no change between 0 minutes and 3 minutes while the sample sat in the dark after photolysis.

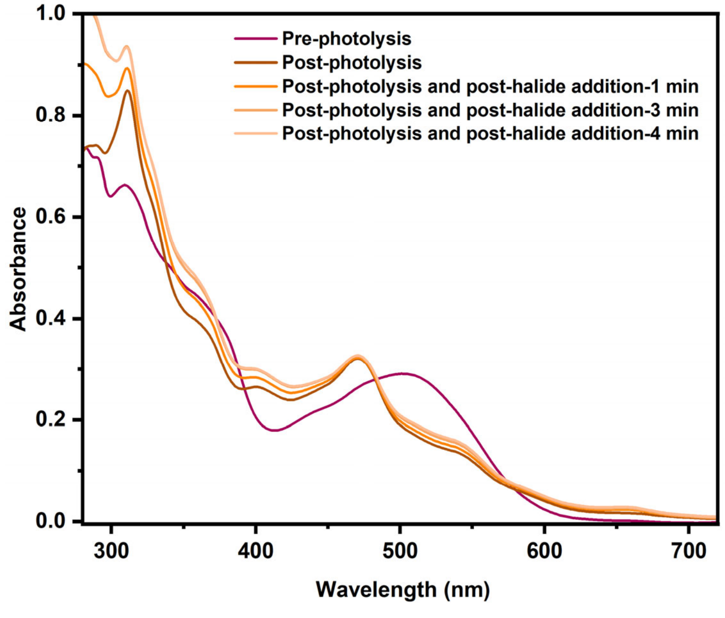


**Figure S47.** Photolysis of PhHxCbl in DMSO which formed Co(II) followed by addition of excess 1-bromopropane. The formed Co(II) is non-reactive towards the alkyl halide.

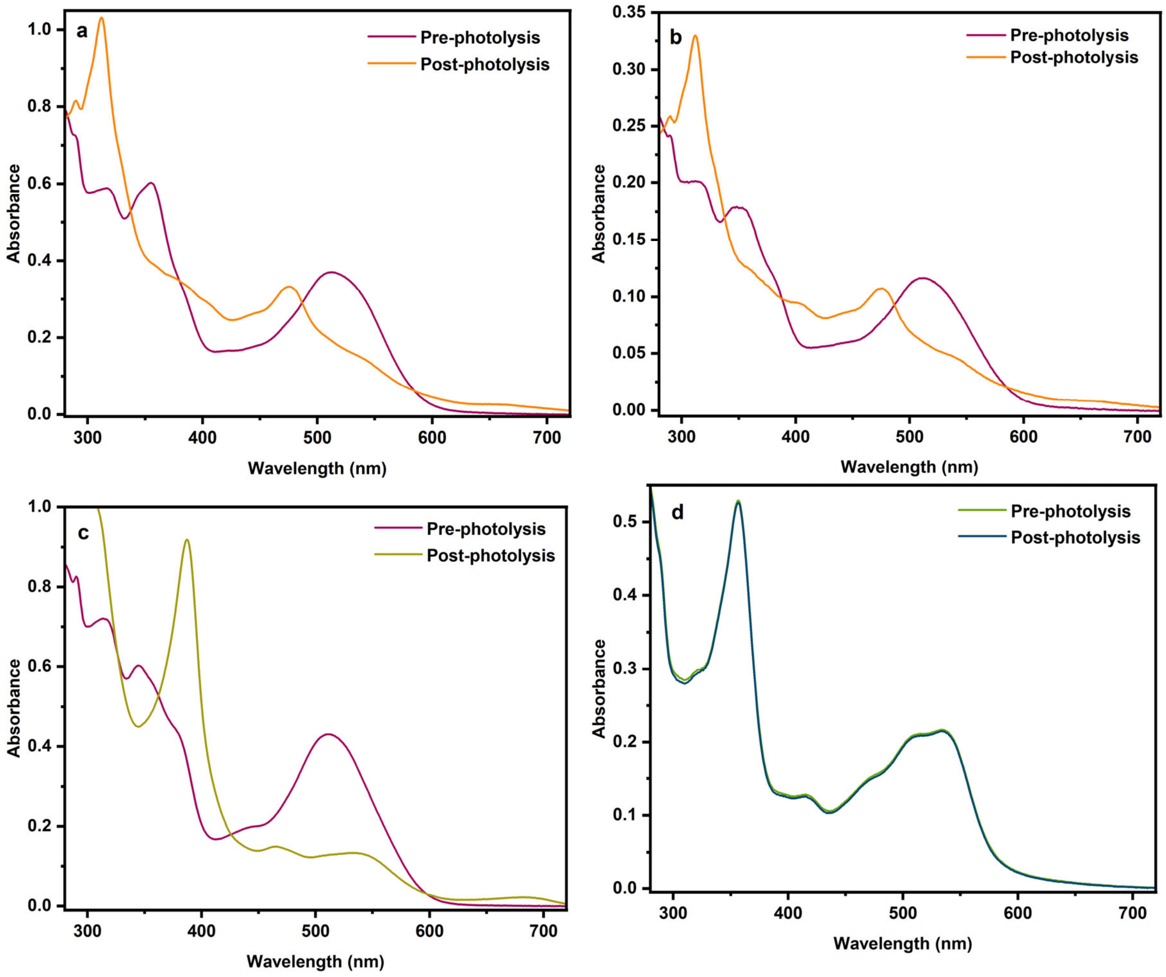


**Figure S48.** (a) Photolysis of 1:1 solution (0.06 mM) of PrCbl and $H_2OCbl$ in water which formed Co(II). (b) Photolysis of a more diluted aqueous solution (<0.03 mM) of PrCbl and $H_2OCbl$ which formed Co(II). (c) Photolysis of PrCbl in water showing formation of Co(I). (d) Photolysis of $H_2OCbl$ showing no change.

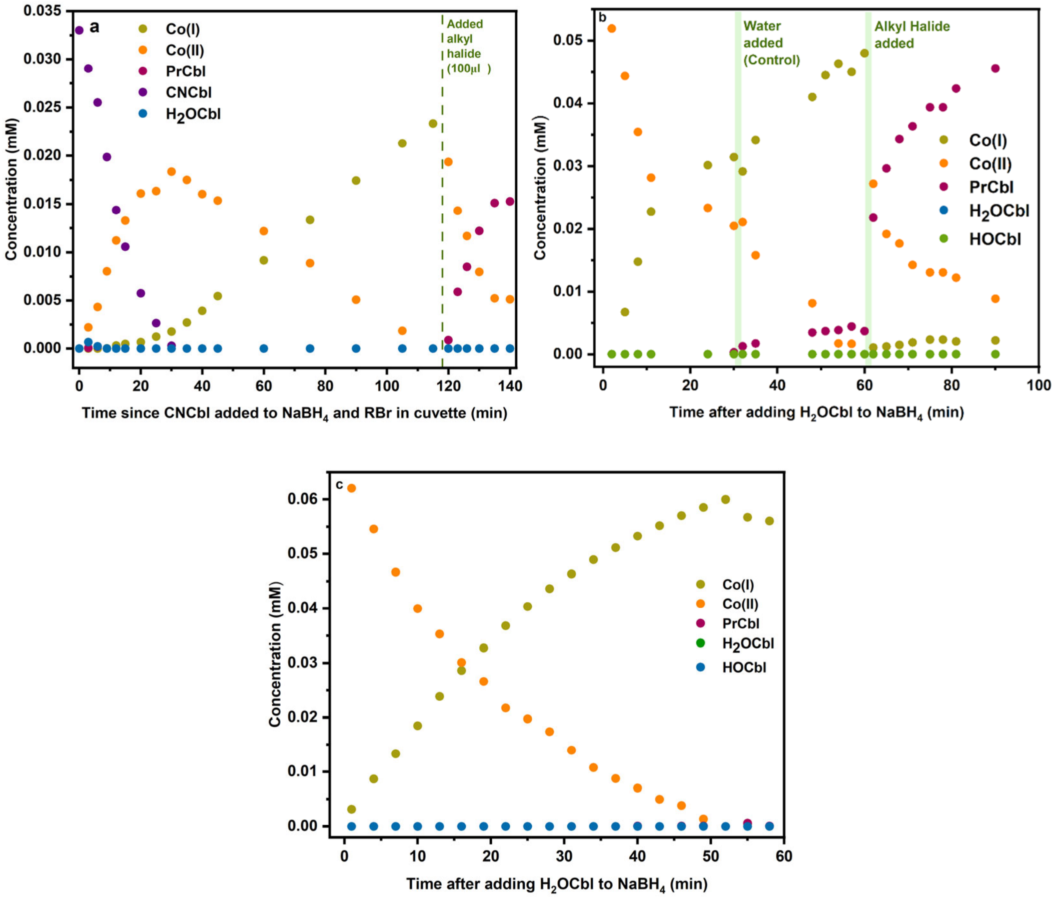


**Figure S49.** Fitted concentrations of different cobalamin species over time shows (a) gradual consumption of CNCbl in presence of $NaBH_4$ and formation of Co(II) that slowly gets consumed to generate Co(I) via $NaBH_4$. Upon adding 100 µL of 1-bromopropane to Co(I) shows initial formation of Co(II), which gets gradually consumed with a concomitant increase in PrCbl concentration. (b) Gradual reduction from $H_2OCbl$ to Co(I) using $NaBH_4$, with part-way addition of water as a control, followed by alkyl halide addition, finally resulting in the generation of PrCbl. (c) Immediate formation of Co(II) upon adding to $H_2OCbl$ $NaBH_4$, followed by the slow reduction to Co(I).

## *7. EPR Measurements:*

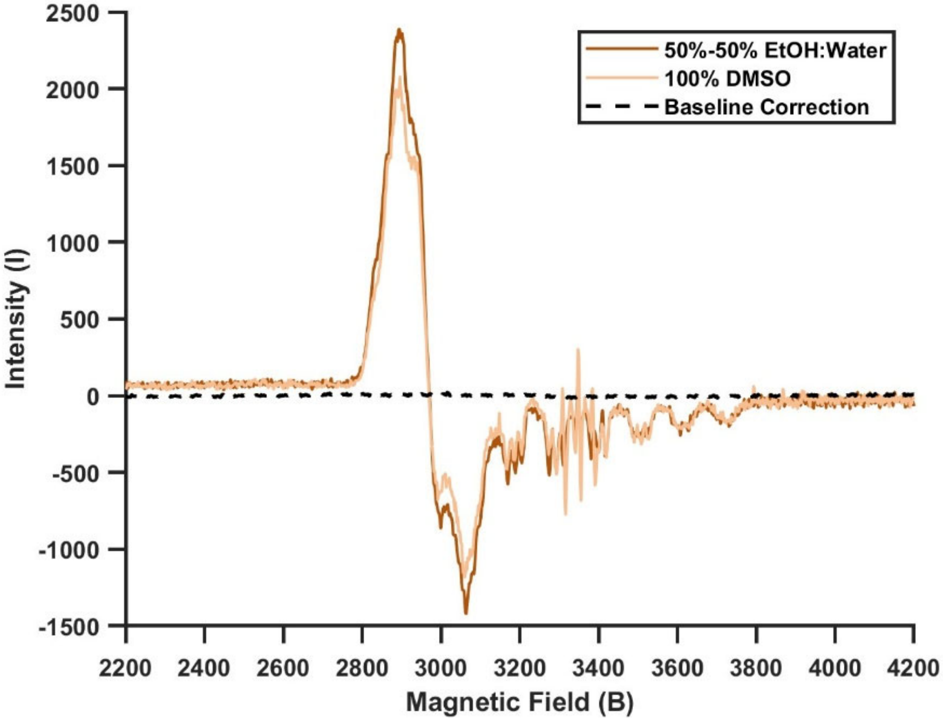


**Figure S50.** X-band (9.37 GHz) continuous wave EPR data measured at 10 K and 0.1 mW power for the PhHxCbl samples in 50%-50% ethanol:water and 100% DMSO. Samples were prepared at 0.6 mM in an anaerobic EPR tube and frozen in liquid nitrogen. A polynomial curve fit to a set of baseline data has been added, it draws a quadratic curve ($y=ax^2+bx+c$) that best approximates the data points. The receiver modulation amplitude is 10 G; relative receiver gain is 4.47e+003 and the rapid scan time constant is 81.92.

## 8. *GC-MS Analysis:*

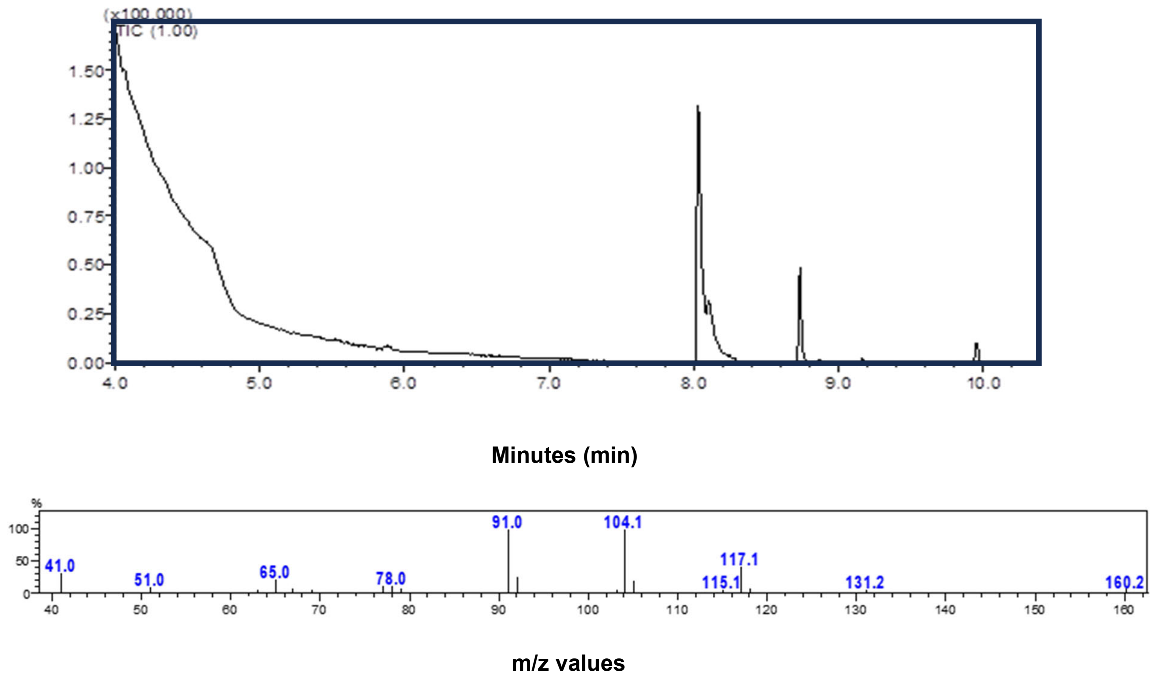


**Figure S51.** GC-MS data for organic product extraction from PhHxCbl photolyzed in DMSO. The product eluting at ~8.0 minutes on the chromatogram is identified as the alkene product, 6-phenyl-1-hexene, as verified by comparison with the standard. The corresponding fragmentation pattern (bottom) shows the parent peak at m/z = 160.2.

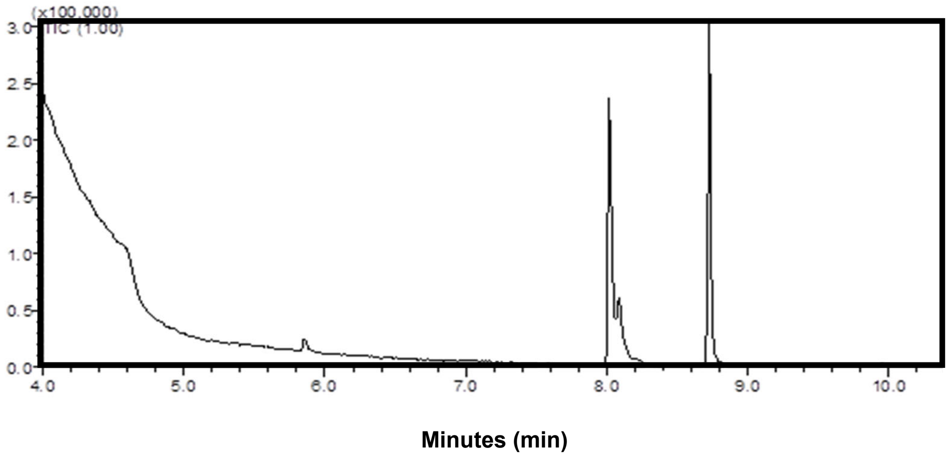


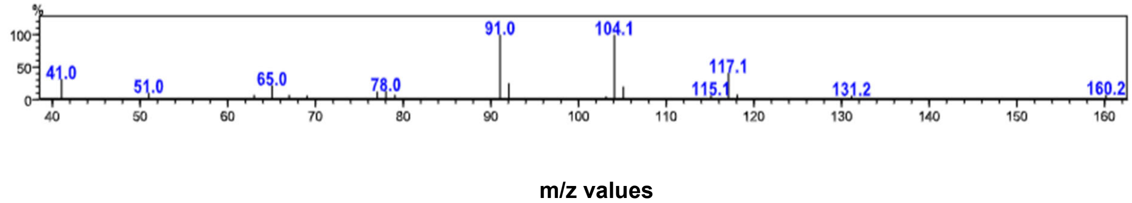


**Figure S52.** GC-MS data for organic product extraction from PhHxCbl photolyzed in DMSO in the presence of sodium bicarbonate (30 equivalents). The product eluting at ~8.0 minutes on the chromatogram is identified as the alkene product, 6-phenyl-1-hexene, as verified by comparison with the standard. The corresponding fragmentation pattern (bottom) shows the parent peak at m/z = 160.2.

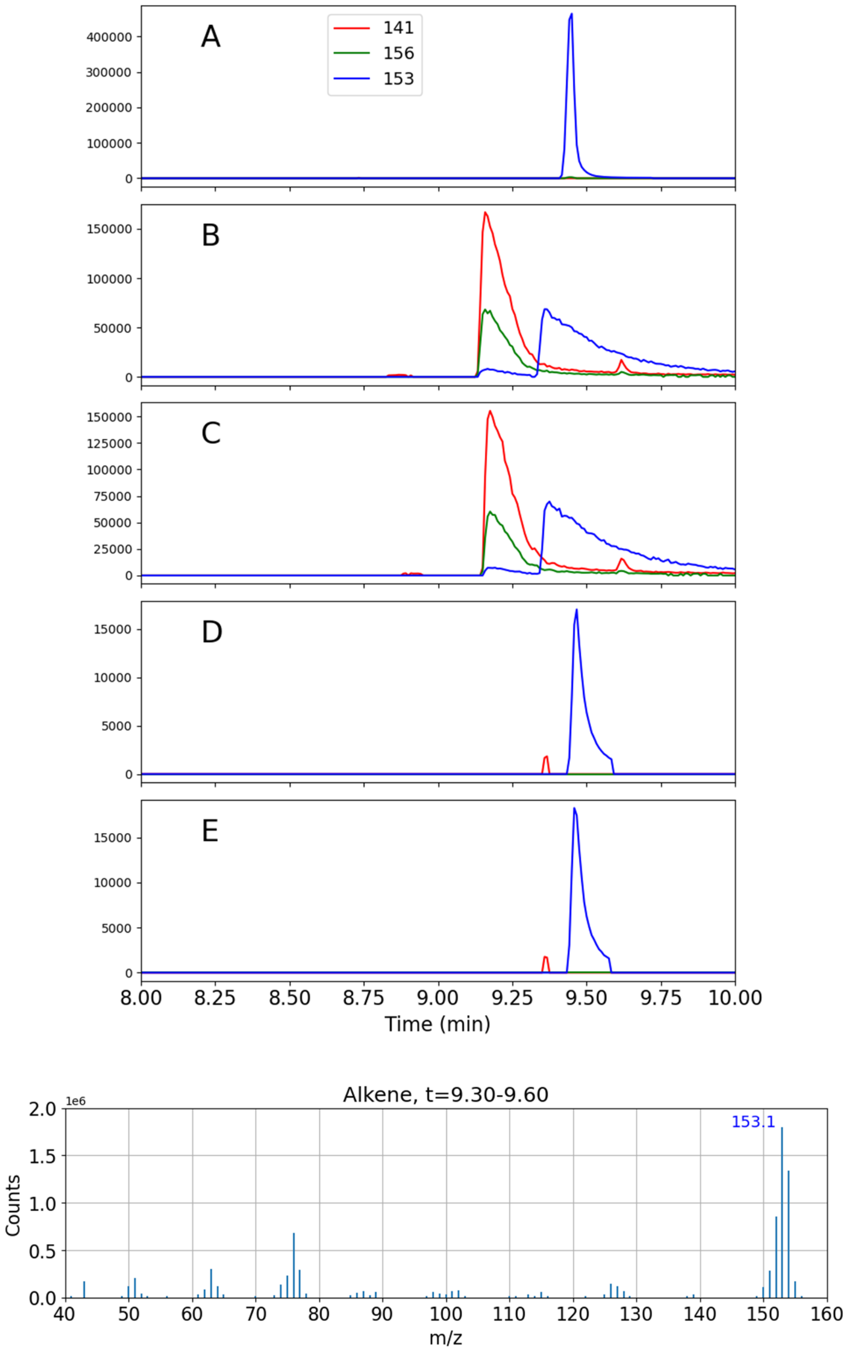


**Figure S53.** GC-MS data for organic product extraction from photolysis of NapEtCbl. (A) 1-vinylnaphthalene standard. (B) NapEtCbl photolyzed in DMSO. (C) NapEtCbl photolyzed in DMSO in the presence of $NaHCO_3$ (30 equivalents). (D) NapEtCbl photolyzed in water. (E) NapEtCbl photolyzed in water in the presence of $NaHCO_3$ (30 equivalents). The product eluting at ~9.45 minutes is identified as the alkene product, 1-vinylnaphthalene. The corresponding fragmentation pattern (bottom) shows the 1-vinylnaphthalene peak at m/z = 154.1. m/z=156 (alkane) is only seen in DMSO.

**Extracted ion count analysis for PhHxCbl in different solvents:**

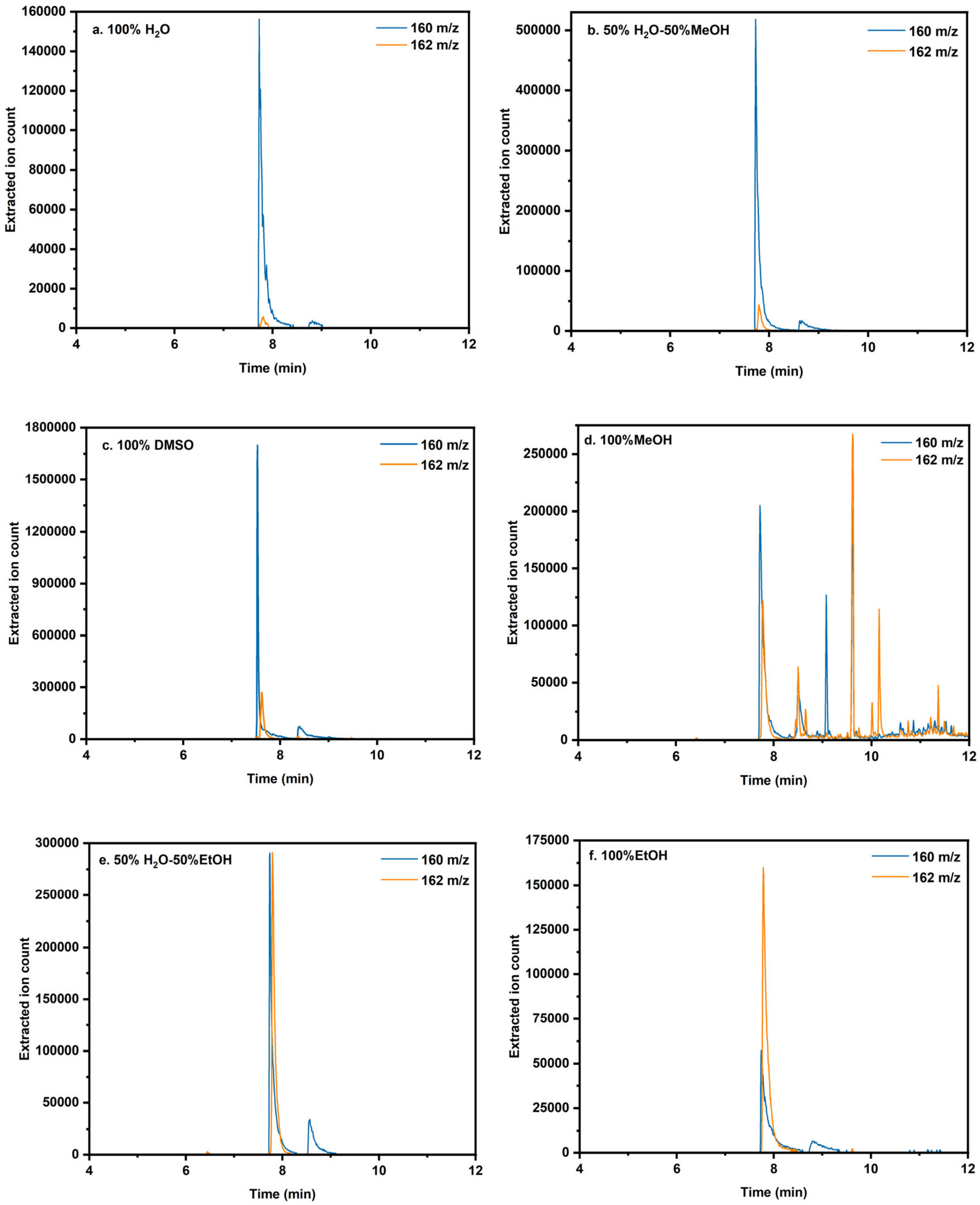

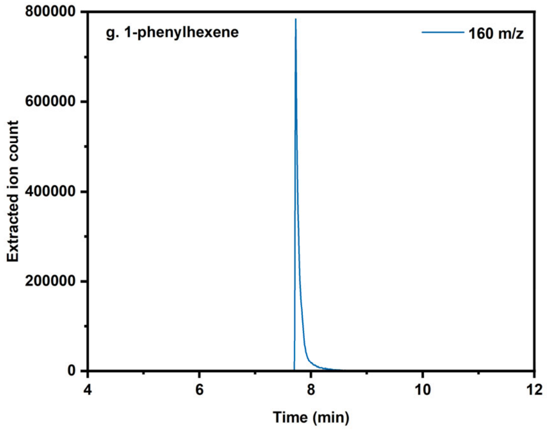


**Figure S54.** Extracted ion counts from GC-MS data for m/z = 160 and m/z = 162 for organic product extraction from photolysis of PhHxCbl in (a) 100% water, (b) 50% water - 50% methanol, (c) 100% DMSO, (d) 100% methanol, (e) 50% water - 50% ethanol, (f) 100% ethanol. (g) Extracted ion counts for the alkene standard, 6-phenyl-1-hexene.

To calculate the amount of alkene (m/z = 160) or alkane (m/z = 162) product produced for PhHxCbl, the extracted ion counts of each parent peak were summed between 7.5 to 8.2 minutes, the time range where complete elution of the standard was observed. The proportion of alkene produced was then calculated as alkene / (alkene + alkane).

For NapEtCbl, the extracted ion counts of each parent peak (alkene: m/z = 154, alkane: m/z = 156) were summed between 9.2 to 10.0 minutes.

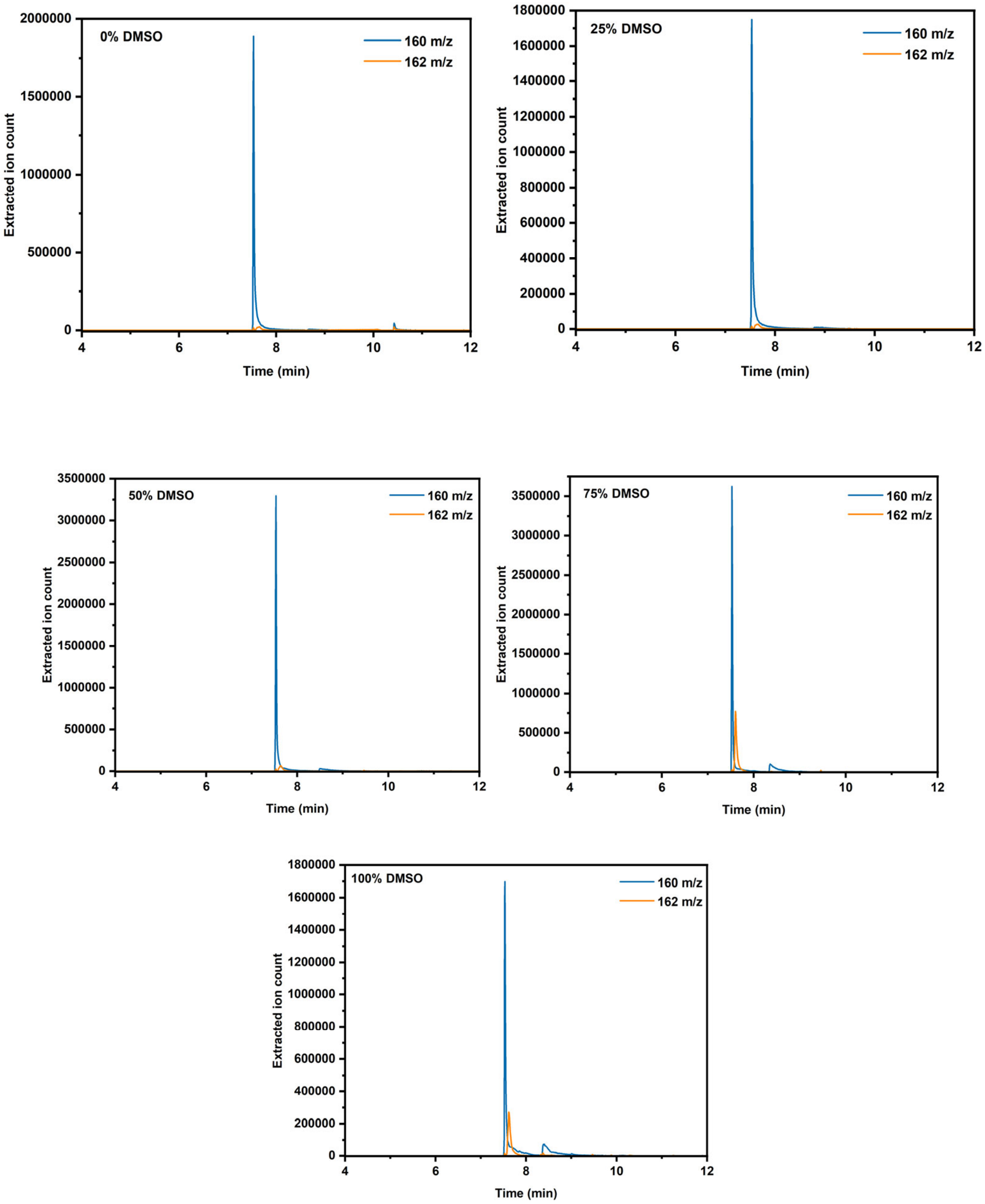


**Figure S55.** Extracted ion counts from GC-MS data for m/z = 160 and m/z = 162 for organic product extraction from photolysis of PhHxCbl in increasing DMSO percentage (v/v) in water. As % DMSO increases, the product mixture shifts slightly more towards the alkane product, but the alkene remains the major product.

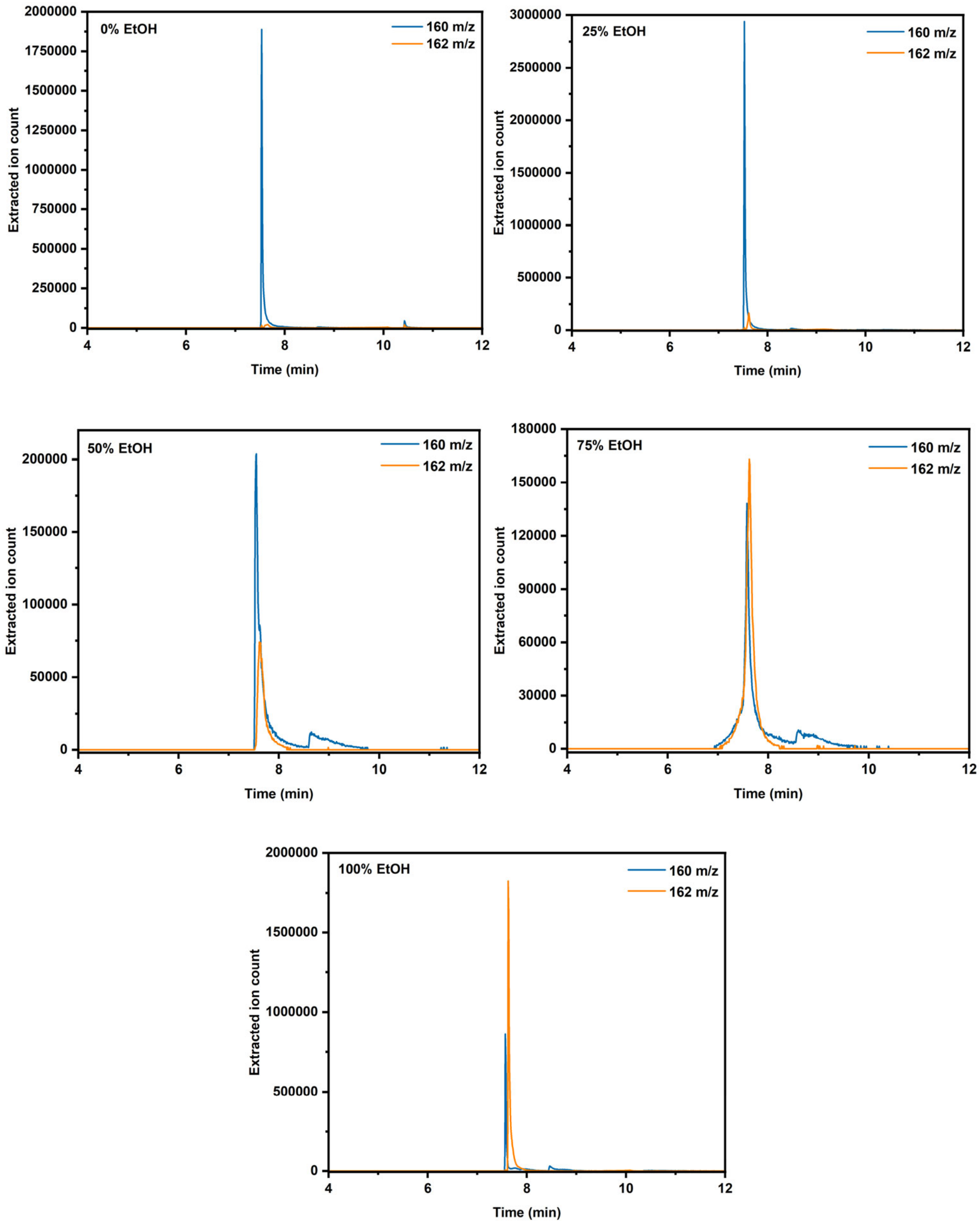


**Figure S56.** Extracted ion counts from GC-MS data for m/z = 160 and m/z = 162 for organic product extraction from photolysis of PhHxCbl in increasing EtOH percentage (v/v) in water. As % EtOH increases, the product mixture heavily shifts towards the alkane product.

**Table S4.** Ratio of alkene / (alkene + alkane) for PhHxCbl with increasing DMSO percentage (v/v) in water obtained via GC-MS mixture experiments.

| Solvent (v/v) | alkene / (alkene + alkane) |
|---|---|
| 100% water | 0.98 ± 0.001 |
| 75-25% water-DMSO | 0.99 ± 0.01 |
| 50-50% water-DMSO | 0.99 ± 0.01 |
| 25-75% water-DMSO | 0.94 ± 0.07 |
| 100% DMSO | 0.86 ± 0.00 |

**Table S5.** Ratio of alkene / (alkene + alkane) for PhHxCbl with increase in ethanol percentage (v/v) in water obtained via GC-MS mixture experiments.

| Solvent (v/v) | alkene / (alkene + alkane) |
|---|---|
| 100% water | 0.98 ± 0.001 |
| 75-25% water-EtOH | 0.95 |
| 50-50% water-EtOH | 0.82 |
| 25-75% water-EtOH | 0.62 |
| 100% EtOH | 0.44 ± 0.015 |

**Table S6.** Ratio of alkene / (alkene + alkane) for PhHxCbl and NapEtCbl in water and DMSO, with and without the presence of sodium bicarbonate (30 equivalents).

| Photolyzed Sample | alkene / (alkene + alkane) |
|---|---|
| PhHxCbl in water | 0.98 ± 0.001 |
| PhHxCbl in water with $NaHCO_3$ | 0.99 ± 0.008 |
| PhHxCbl in DMSO | 0.86 ± 0.008 |
| PhHxCbl in DMSO with $NaHCO_3$ | 0.88 ± 0.03 |
| NapEtCbl in water | a |
| NapEtCbl in water with $NaHCO_3$ | a |
| NapEtCbl in DMSO | b |
| NapEtCbl in DMSO with $NaHCO_3$ | b |

a. Only alkene was detected
b. Both alkane (m/z=141 & 156) and alkene were detected

## 9. *Reduction mechanism:*

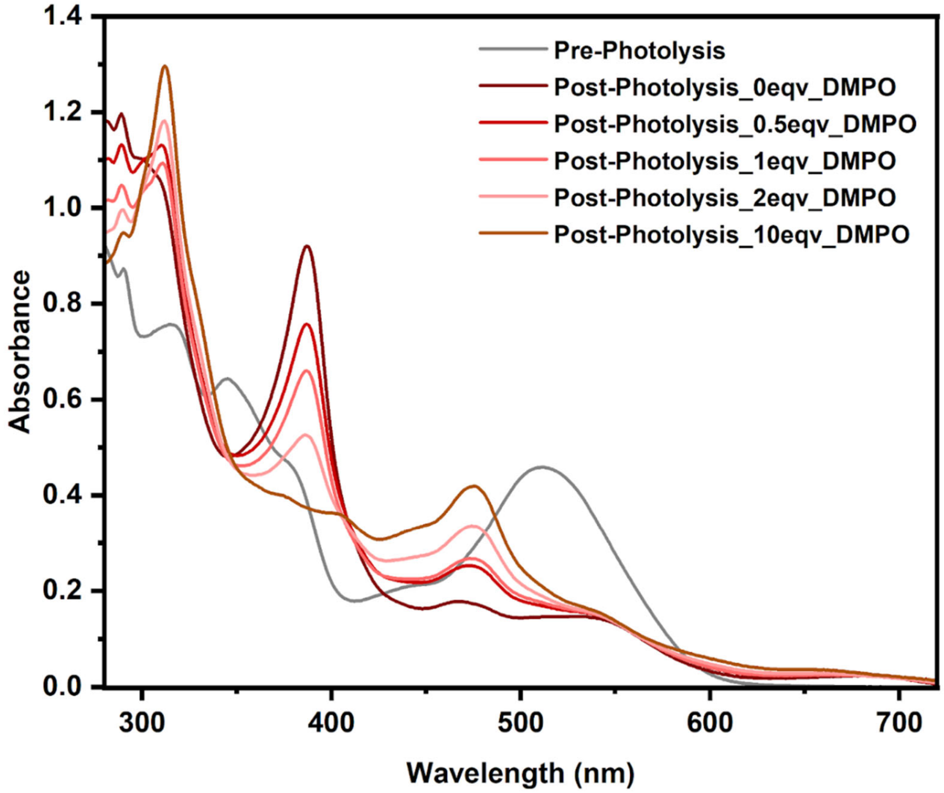


**Figure S57.** UV-Vis absorption spectra of PrCbl in water before and after photolysis for 2 minutes in the presence of different equivalents of DMPO showing decrease in Co(I) formation accompanied by increase in Co(II) formation.

**Table S7.** Fits obtained for PrCbl photolysis in water with different equivalents of DMPO added (equivalents are relative to PrCbl).

| Sample | %Co(I) | %Co(II) | %R-Co(III) | $H_2OCbl$ | HOCbl |
|---|---|---|---|---|---|
| Pre-photolysis 0 equivalents of DMPO | 3 | 8 | 89 | 0 | 0 |
| Post-photolysis 0 equivalents of DMPO | 85 | 7 | 8 | 0 | 0 |
| Post-photolysis 0.5 equivalents of DMPO | 56 | 37 | 7 | 0 | 0 |
| Post-photolysis 1 equivalents of DMPO | 46 | 46 | 8 | 0 | 0 |
| Post-photolysis 2 equivalents of DMPO | 26 | 69 | 5 | 0 | 0 |
| Post-photolysis 10 equivalents of DMPO | 5 | 93 | 2 | 0 | 0 |

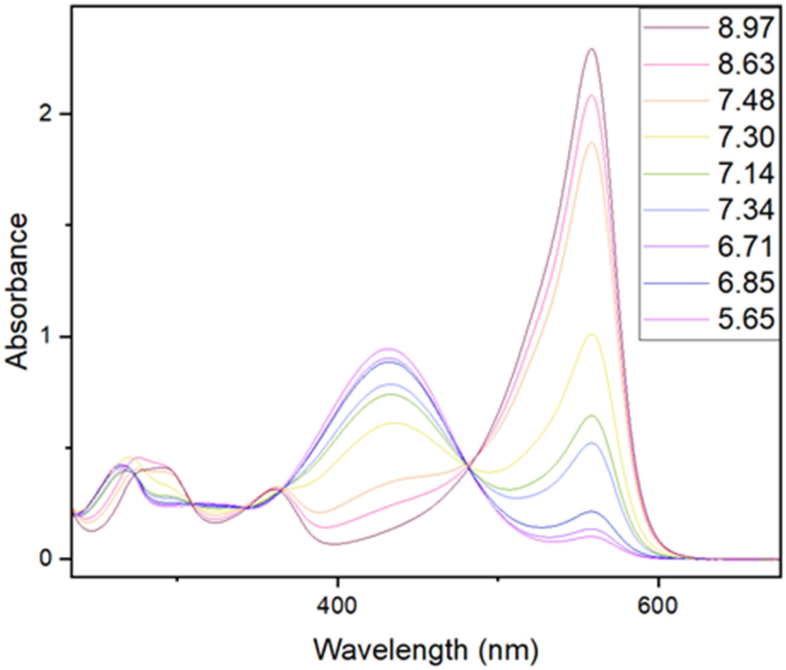


**Figure S58.** Phenol red (0.04 mM solutions in water) UV-Vis spectra at different pH values as shown in the figure legend.

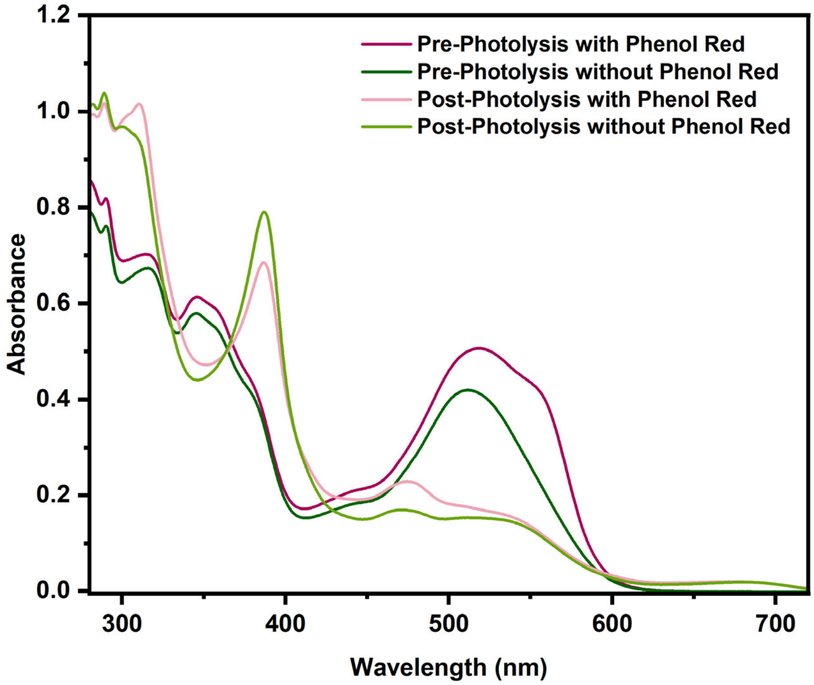


**Figure S59.** UV-Vis spectra for photolysis with (pink) and without (green) phenol red dissolved in aqueous solution of PrCbl.

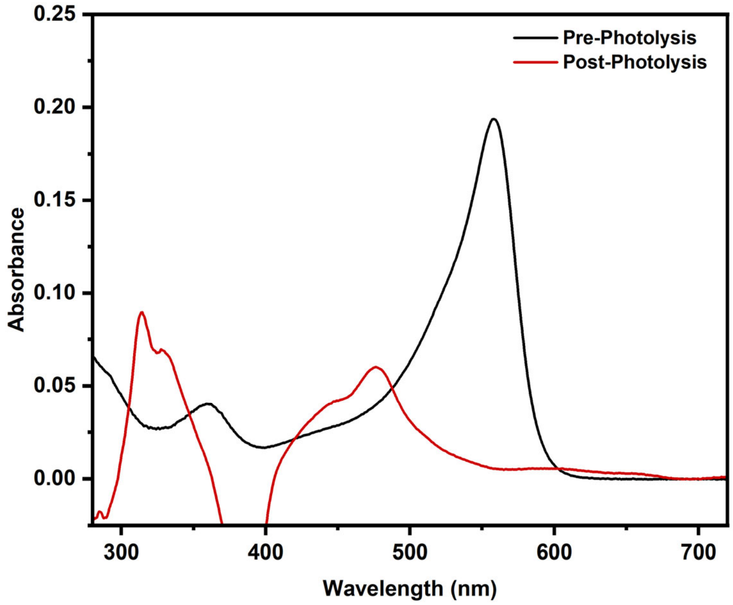


**Figure S60.** Residual absorbance from phenol red. Spectra plotted are spectrum with phenol red minus spectrum without phenol red. Negative feature at ~390 nm reflects a small (~10%) conversion of Co(I) back to Co(II) through reduction of phenol red.

**Table S8.** Ratio of alkene / (alkene + alkane) and ratio between fragments m/z 163 over m/z 162 for PhHxCbl in both non-deuterated and deuterated solvents obtained via GC-MS mixture experiments.

| Sample | Alkene/ (alkane + alkene) | m/z fragment 163/162 | Deuteration |
|---|---|---|---|
| EtOH ($H_2O$ workup) | 0.43 | 0.13 | -- |
| Ethanol-*d*$_1$ ($H_2O$ workup) | 0.32 | 0.13 | 0% |
| Ethanol-*d*$_6$ ($D_2O$ workup) | 0.95 | 0.87 | 43% |
| Ethanol-*d*$_6$ ($H_2O$ workup) | 0.95 | 0.80 | 40% |
| Methanol-*d*$_4$ ($H_2O$ workup) | 0.91 | 0.20 | 7% |

**Note:** These experiments were carried out by photolyzing PhHxCbl in the respective solvents (2 ml) as listed in the table above, followed by 1:1 dilution with either $H_2O$ or $D_2O$ (as specified in the table) and then finally performing a liquid-liquid extraction using 1 ml of hexanes, after which the samples were loaded in the GC-MS instrument for analysis. The proteoalkane 163/162=0.13 as expected from the natural abundance of $^{13}C$. The increase in this ratio for deuterated solvents reflects partial incorporation of D into the product.

# *10.References:*


1. Wiley, T. E.; Miller, N. A.; Miller, W. R.; Sofferman, D. L.; Lodowski, P.; Toda, M. J.; Jaworska, M.; Kozlowski, P. M.; Sension, R. J. Off to the Races: Comparison of Excited State Dynamics in Vitamin B12 Derivatives Hydroxocobalamin and Aquocobalamin. *J. Phys. Chem. A* **2018**, *122*, 6693-6703.
2. Wiley, T. E.; Miller, W. R.; Miller, N. A.; Sension, R. J.; Lodowski, P.; Jaworska, M.; Kozlowski, P. M. Photostability of Hydroxocobalamin: Ultrafast Excited State Dynamics and Computational Studies. *J. Phys. Chem. Lett.* **2016**, *7*, 143-147.
3. Shiang, J. J.; Walker, L. A.; Anderson, N. A.; Cole, A. G.; Sension, R. J. Time-Resolved Spectroscopic Studies of B12 Coenzymes: The Photolysis of Methylcobalamin Is Wavelength Dependent. *J. Phys. Chem. B* **1999**, *103*, 10532-10539.
4. Cole, A. G.; Yoder, L. M.; Shiang, J. J.; Anderson, N. A.; Walker, L. A.; Banaszak Holl, M. M.; Sension, R. J. Time-Resolved Spectroscopic Studies of $B_{12}$ Coenzymes: A Comparison of the Primary Photolysis Mechanism in Methyl-, Ethyl-, *n*-Propyl-, and 5′-Deoxyadenosylcobalamin. *J. Am. Chem. Soc.* **2002**, *124*, 434-441.
5. Sension, R. J.; McClain, T. P.; Lamb, R. M.; Alonso-Mori, R.; Lima, F. A.; Ardana-Lamas, F.; Biednov, M.; Chollet, M.; Chung, T.; Deb, A.; Dewan, P. A., Jr.; Gee, L. B.; Huang Ze En, J.; Jiang, Y.; Khakhulin, D.; Li, J.; Michocki, L. B.; Miller, N. A.; Otte, F.; Uemura, Y.; van Driel, T. B.; Penner-Hahn, J. E. Watching Excited State Dynamics with Optical and X-ray Probes: The Excited State Dynamics of Aquocobalamin and Hydroxocobalamin. *J. Am. Chem. Soc.* **2023**, *145*, 14070-14086.
